# Amplification sensing manipulated by a sumanene-based supramolecular polymer as a dynamic allosteric effector

**DOI:** 10.1038/s41598-024-63304-4

**Published:** 2024-05-31

**Authors:** Hiroaki Mizuno, Hironobu Nakazawa, Akihisa Miyagawa, Yumi Yakiyama, Hidehiro Sakurai, Gaku Fukuhara

**Affiliations:** 1 Department of Chemistry, Tokyo Institute of Technology, 2-12-1 Ookayama, Meguro-Ku, Tokyo, 152-8551 Japan; 2 Division of Applied Chemistry, Graduate School of Engineering, Osaka University, Suita, Osaka 565-0871 Japan; 3 Department of Chemistry, Faculty of Pure and Applied Sciences, University of Tsukuba, Tsukuba, Ibaraki 305-8577 Japan; 4 Division of Applied Chemistry, Graduate School of Engineering and Innovative Catalysis Science Division, Institute for Open and Transdisciplinary Research Initiatives (ICS-OTRI), Osaka University, Suita, Osaka 565-0871 Japan

**Keywords:** Sumanene, Supramolecular polymer, Allosterism, Amplification sensing, Supramolecular polymers, Supramolecular chemistry

## Abstract

The synthesis of signal-amplifying chemosensors induced by various triggers is a major challenge for multidisciplinary sciences. In this study, a signal-amplification system that was flexibly manipulated by a dynamic allosteric effector (trigger) was developed. Herein, the focus was on using the behavior of supramolecular polymerization to control the degree of polymerization by changing the concentration of a functional monomer. It was assumed that this control was facilitated by a gradually changing/dynamic allosteric effector. A curved-π buckybowl sumanene and a sumanene-based chemosensor (SC) were employed as the allosteric effector and the molecular binder, respectively. The hetero-supramolecular polymer, (SC·(sumanene)_n_), facilitated the manipulation of the degree of signal-amplification; this was accomplished by changing the sumanene monomer concentration, which resulted in up to a 62.5-fold amplification of a steroid. The current results and the concept proposed herein provide an alternate method to conventional chemosensors and signal-amplification systems.

## Introduction

Chemical sensors (chemosensors), such as artificial receptors and supramolecular synthetic hosts, have rapidly developed in recent years as a multidisciplinary science, which incorporates mainly supramolecular and analytical chemistry^1–17^. Recently, smart chemosensors have been used for real-time apoptosis detection^18–20^, as well as basic molecular recognition. The range of applications of chemosensors is based on the lock-and-key principle (enzyme–substrate model) proposed by Emil Fischer^21^. However, further advancements are required to address the overwhelming of conventional chemosensors.

**Figure 1:**
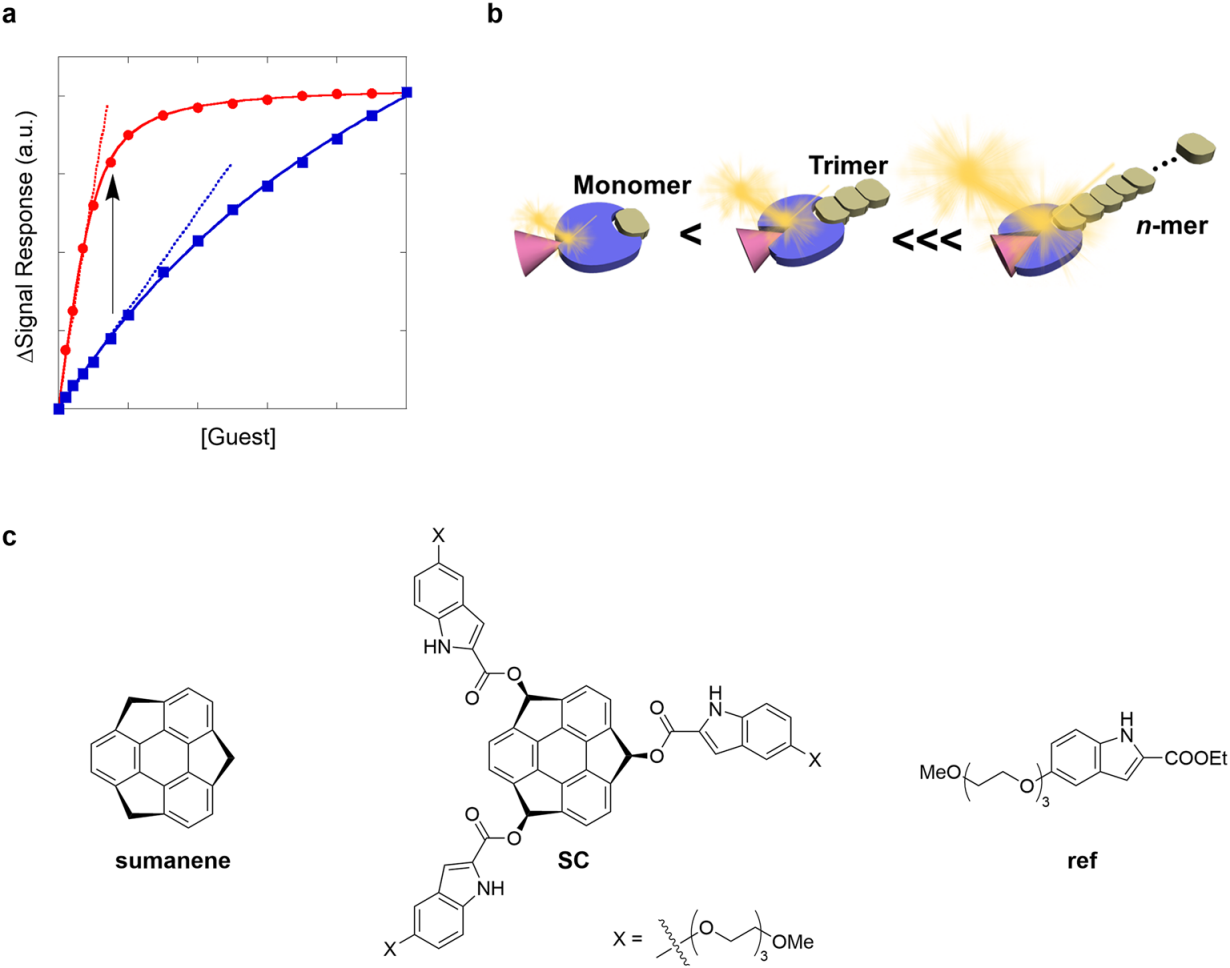
Concept and design guidelines of the chemosensors. (**a**) Model titration curves (lower (blue) and higher (red) binding constants), assuming the 1:1 stoichiometric complexation represents each nonlinear least-squares binding isotherm. The black arrow indicates signal amplification. (**b**) Schematic of the concept for the dynamic allosteric effector that can flexibly manipulate binding equilibria via supramolecular polymerization. The red and blue pieces show a guest and chemosensor (artificial receptor), respectively. (**c**) Chemical structures of sumanene as a monomer for supramolecular polymers, SC as a chemosensor, and ref as a reference compound, respectively.

Amplifying the signal is an effective solution for chemosensor overload because it minimizes the disadvantages of the lock-and-key model^22–24^, resulting in enhanced sensitivity. This has been demonstrated in Fig. 1a, where the signals (blue curve) against the guest concentration exhibited a typical nonlinear least-square binding isotherm when the complexation type was 1:1; the blue slope represents sensitivity^25^. The enhancement of a binding constant (*K*), triggered by an amplifying mechanism, also amplified the signals (red curve) in the initial-to-middle section, resulting in increased sensitivity (red slope). Biological systems can amplify *K* through methods such as allosterism^26–32^. Hemoglobin is a well-known example of an allosteric system^33^. Once an O_2_ molecule binds to hemoglobin, a conformational change is induced in the protein, enabling further addition of O_2_ molecules. Homotropic allosterism is an allosteric system wherein the target molecule simultaneously functions as an effector (hemoglobin). By contrast, heterotropic allosterism is an allosteric system that requires a different effector to enhance the binding of the target molecule.

Biological systems have shown that some contrivance can be applied to an allosteric effector. This resulted in exploring a nature-surpassed dynamic effector that can control and then suppress/promote a binding equilibration; that is, dynamic control of signal amplification. This effector can exploit supramolecular polymerization. Supramolecular polymers consist of a functional monomer that spontaneously stacks on each other through noncovalent interactions, such as π–π, hydrogen bonding, and electrostatic interactions^34–42^. Porphyrins^43^ and perylene bisimides^44^ have been widely used as π-plane monomers. Therefore, it was assumed that the degree of polymerization (DP) can be manipulated by changing the monomer concentration. This will likely result in a gradual and dynamic change in the monomer characteristics as an allosteric effector (monomer, dimer, trimer, ···, *n*-mer) (Fig. 1b).

In this study, a novel, nature-surpassed signal-amplification system was discovered. Within this system, dynamic changes in the allosteric effector occurred via supramolecular polymerization adjustments, which were for the first time achieved by using curved-π buckybowl sumanene^45^ (Fig. 1c) as the monomer. A recent study showed that pristine sumanene spontaneously forms supramolecular polymers in solutions in an isodesmic manner^46^—*K*_n_ (nucleation) = *K*_e_ (elongation). Therefore, a sumanene-based chemosensor (SC, Fig. 1c) was constructed based on the guideline shown in Fig. 1b. Pristine sumanene gradually stacks on the convex face of the chemosensor to form hetero-supramolecular polymers (SC·(sumanene)_n_). The likelihood of the stacking-sumanene moieties in the hetero-supramolecular polymers perturbing the electronic properties of the molecular recognition sites in SC was high. Here, we report an unprecedented signal-amplification system based on the hetero-supramolecular polymers composed of SC as a molecular binder and sumanene as a dynamic allosteric effector. In this case, the signal amplifications were induced from fluorescence changes upon the complexation of guest molecules. This concept resulted in a powerful, widely applicable chemosensor capable of manipulating signal amplification.

## Results and discussion

### Photophysical properties of SC

**Figure 2:**
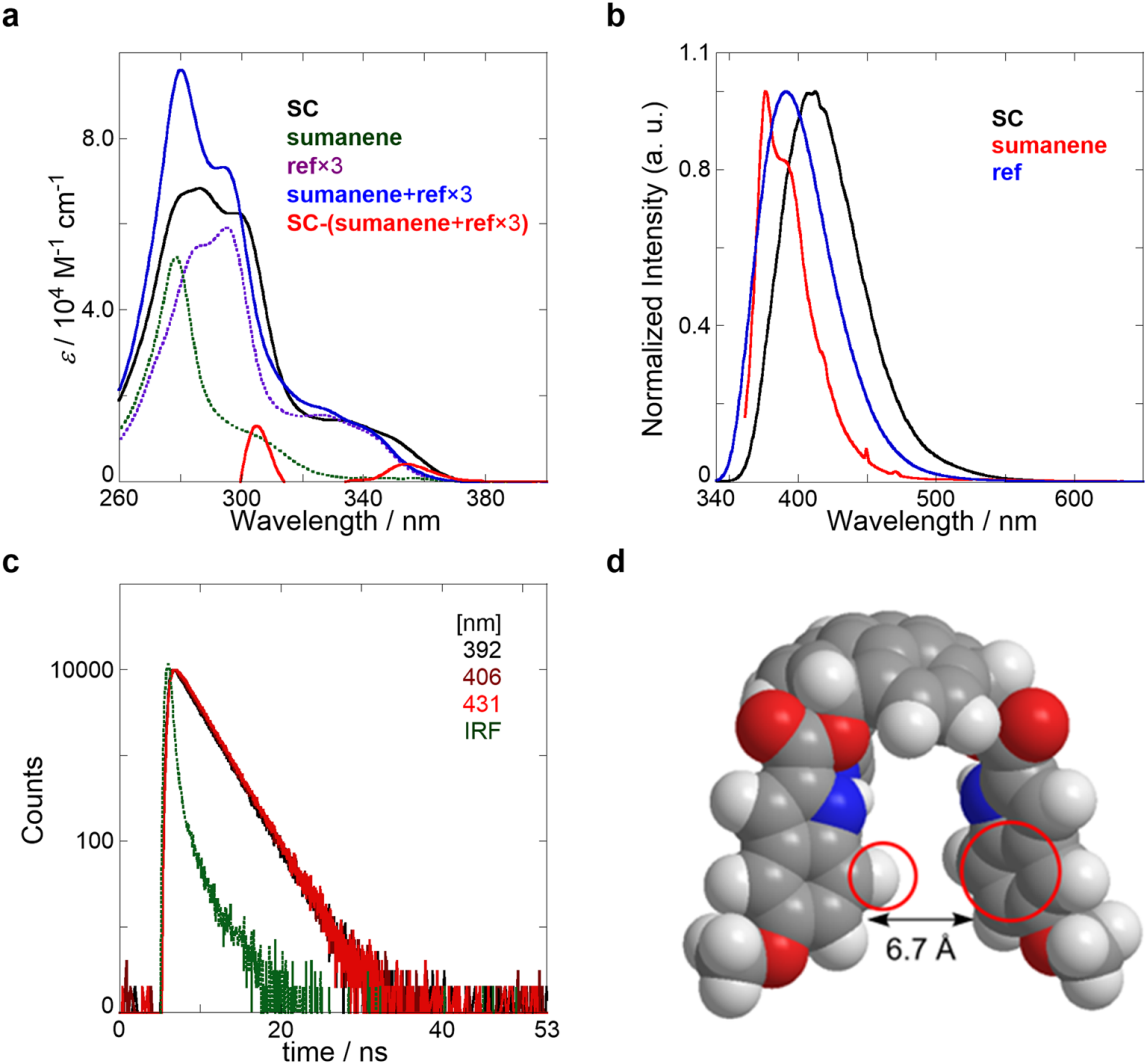
Photophysical properties of SC. (**a**) UV/vis absorption spectra of SC (8.3 μM, black solid line), sumanene (9.8 μM, green dotted line), and ref (15.6 μM, purple dotted line, three times) in CH_2_Cl_2_ at 25 °C. The red solid line represents the subtraction spectrum. (**b**) Fluorescence spectra of SC (black, λ_ex_: 330 nm), sumanene (red, λ_ex_: 351 nm), and ref (blue, λ_ex_: 330 nm) in CH_2_Cl_2_ at 25 °C, measured in a 1 cm cell. (**c**) Fluorescence lifetime decays (λ_ex_: 340 nm) of SC monitored at 392 (black), 406 (brown), and 431 nm (red) in CH_2_Cl_2_ at room temperature, measured in a 1 cm cell; the green dotted line represents instrument response function (IRF). (**d**) Quantum chemistry calculation-based optimized structure of **SC**.

The UV/vis absorption and fluorescence spectra, and fluorescence lifetime decays of SC in dichloromethane (CH_2_Cl_2_) are shown in Fig. 2 and Fig. S12 in Supplementary Information (SI). The maximum peak in the UV/vis absorption spectra of SC was observed at approximately 280 nm (Fig. 2a), which was similar to the sum of sumanene and the indole reference compound (ref, Fig. 1c). However, new absorption bands at approximately 310 and 360 nm were observed after comparing the molar extinction coefficients of SC with those of sumanene and ref, suggesting that a π-conjugation extended from the sumanene core to the indole chromophore (see Fig. 6 (top)). An emission peak at 412 nm was observed in the fluorescence spectrum of SC (Fig. 2b). Because of the appreciable bathochromic shift in SC compared with those of sumanene and ref, this peak may have originated from the π-extended indole-sumanene conjugation. The fluorescence decay profiles (Fig. 2c) monitored at 392, 406, and 431 nm (λ_ex_: 340 nm) were fitted to a sum of reasonable exponential functions to produce the first short-lived species (0.4 ns) at the shorter wavelength region, a major fluorescence species (2.6 ns) presented in the entire region, and the second short-lived species (0.3–0.4 ns) at the longer wavelength region (Table S1 in SI). The major excited species (2.6 ns) was assigned to the fluorescent SC monomer. A titration of SC using triethylamine as an organic base (Figs. S13–S14 and Table S2–S3 in SI) showed the fluorescence quenching mainly involved the first short-lived species; this was assigned to a fluorescent anion species where protons dissociated from the indole moiety. A rise component with a negative *A* factor (relative abundance) was observed in the second short-lived species (Fig. 2c, Figs. S12c,d (SI), and Table S1 (SI)). By contrast, ref mainly showed the major monomeric species (3.2–3.4 ns) without the rise (Fig. S15 and Table S4 in SI). Density functional theory (DFT) calculations (function/basis set: ωB97X-D/6-311G(d,p)) of SC were performed to identify the rise component (Fig. 2d). Three indole side chains aligned in a T-shape manner with a distance of 6.7 Å. The results of the DFT calculations suggested that the second short-lived species is originated from the frustrated (second) excimer formed via the intramolecular, T-shaped overlap of the indole side chains; this was observed in previous studies^47,48^. This interesting photochemical property was formed by the fixation of the fluorescent moieties on the sumanene scaffold.

### Sensing behavior of SC

**Figure 3:**
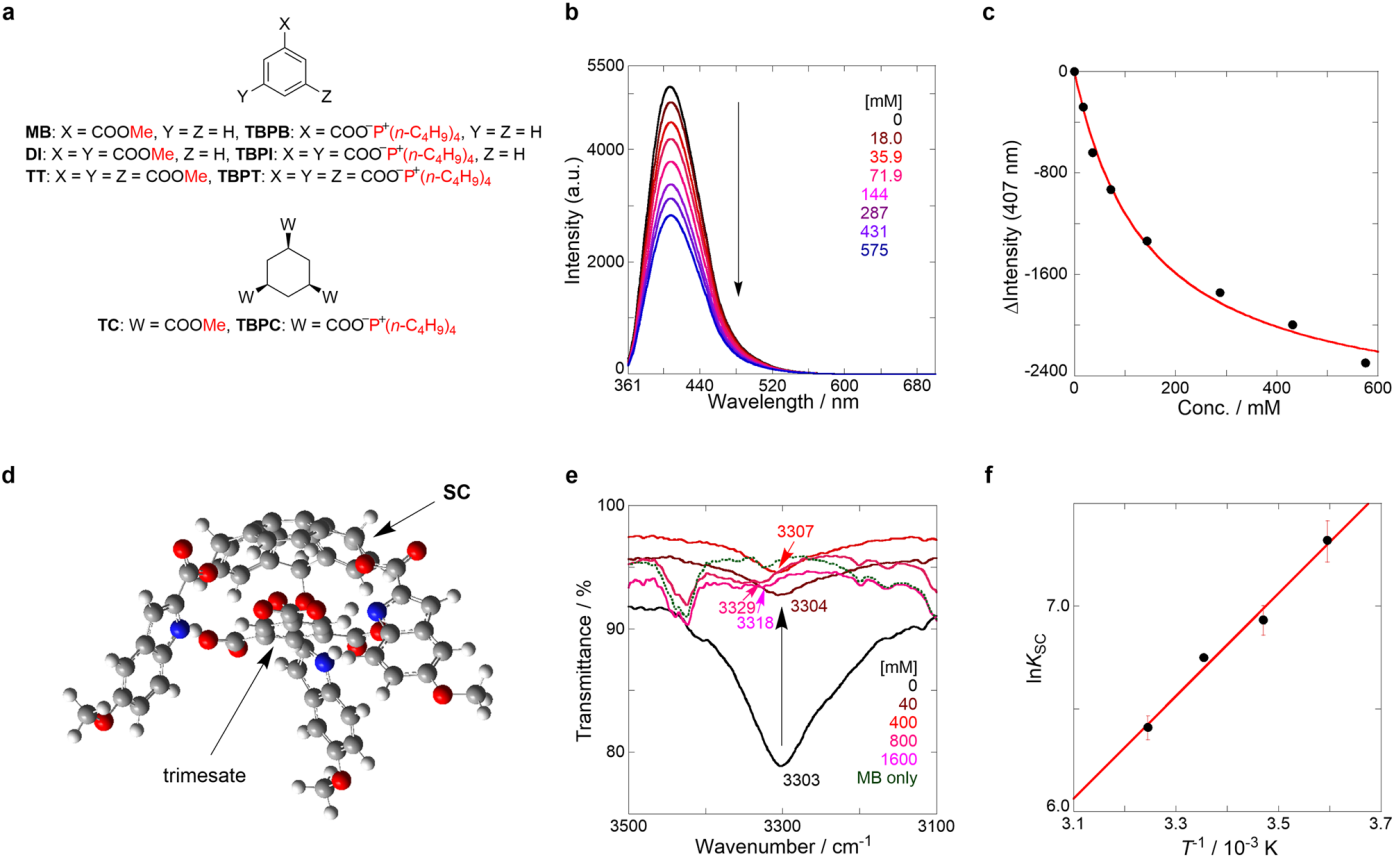
Sensing behavior of SC. (**a**) Chemical structures of model guests. (**b**) Fluorescence spectra (λ_ex_: 351 nm) of SC (421 μM; black) showing the gradual addition of MB (18.0–575 mM; from brown to blue) in CH_2_Cl_2_ at 25 °C, measured in a 1 mm cell; the excitation wavelength at which comparable absorbances were obtained was selected. (**c**) Nonlinear least-squares fitting line (assuming the 1:1 stoichiometry with SC and MB monitored at 407 nm) to determine the binding constant at 25 °C. (**d**) Optimized supramolecular complex structure of SC ⊂ trimesate (C_6_H_3_(COO^−^)_3_); counter cations were omitted for clarity. (**e**) IR spectra of SC (4.2 mM, black) showing the gradual addition of MB (40 mM–1.6 M, brown to pink) in CH_2_Cl_2_ at room temperature, where the green dotted line represents MB (1.6 M) in CH_2_Cl_2_. (**f**) van’t Hoff plot of the binding constants obtained from the complexation of TBPB with SC in CH_2_Cl_2_ (*r* = 0.992).

A series of benzene and cyclohexane derivatives were used as model guests for a proof of concept (Fig. 3a). Thereafter, their sensing behaviors were investigated in CH_2_Cl_2_. Upon the gradual addition of MB, a wide range broadened peak was observed in the long wavelength region (> 355 nm) of the UV/vis absorption spectra for the titration of SC ⊂ MB (Fig. S17a in SI). Efficient quenching was only observed when a new band emerged, and peak shifts were absent in the corresponding fluorescence spectra (Fig. 3b and Fig. S17b (SI)). Using this spectral change, *K*_SC_ was estimated as 7 ± 1 M^−1^, assuming the formation of a 1:1 complexation (Fig. 3c) wherein: (1) the stoichiometric analysis in Fig. S20 in SI was discussed using the following anion system, and (2) the following 1:1 complex structure was provided in advance (Fig. 3d). Subsequently, the anion sensing shown in Figs. S18–S19 in SI was discussed. The UV/vis absorption spectra of SC ⊂ TBPB exhibited a similar broadened wavelength (> 355 nm); however, a hypochromic effect was observed at approximately 320–355 nm. During fluorescence titration, the peak maxima bathochromically shifted with appreciable quenching. The spectral differences between neutral MB and anionic TBPB were likely responsible for the different ground-state complexations based on the neutral- or anion-SC. Furthermore, the *K*_SC_ value of anion-SC, assuming the complexation type was 1:1, was enhanced to 850 ± 10 M^−1^. Therefore, the spectroscopic behaviors were affected by the strength of the anion complexation.

The interaction between SC and MB was further investigated using IR spectroscopy. The broad peak at 3303 cm^−1^, which was derived from the N–H stretching vibration in the indole ring, decreased in intensity as the higher wavenumber shifted to 3329 cm^−1^ with increasing MB concentration (Fig. 3e). The DFT calculation also supports the 1:1 complex structure as SC ⊂ MB (Fig. S20-2 in SI), as was the case with TBPB. Additionally, the ref compound can bind MB (*K*_ref_ = 4 ± 1 M^−1^) and TBPB (*K*_ref_ = 4 ± 1 M^−1^) with sufficient fluorescence quenching; this was also observed in SC (Fig. S16 in SI). These results suggest that the hydrogen bonds formed via the carbonyl moiety of the guests and the dissociated N–H protons on the indole rings in SC are main recognition forces. This was consistent with the fluorescence quenching behavior observed after adding triethylamine (vide supra). The *K*_SC_ value of SC ⊂ TBPB was enhanced by a factor of 213 compared with *K*_ref_ of ref ⊂ TBPB, which was likely owing to a cooperative complexation through the octopus-like three indoles on the sumanene scaffold. Therefore, it was important to elucidate the thermodynamic parameters before applying the temperature-dependent van’t Hoff analysis of SC ⊂ TBPB at four temperatures from 5 to 35 °C (Fig. 3f and Figs. S18–S19 (SI)). The van’t Hoff plot showed a straight line, indicating that the same complexation mechanism can be found in this temperature range (same heat capacity); Δ*H*° and *Τ*Δ*S*° were − 20.8 and − 4.2 kJ mol^−1^, respectively. The thermodynamic parameters exhibited enthalpy-driven complexation. Nevertheless, the observed entropy loss was relatively low despite the immobilization by three recognition sites, which was accounted for by the classical chelate effect^49^. Therefore, the high enthalpy gain and low entropy loss (the common cooperative manner) resulted in a higher and enhanced *K*_SC_ (Δ*G*° = -16.6 kJ mol^−1^) based on the distinctive structural specificity.

The sensing results of the eight guests by SC are listed in Table 1 and Figs. S21–S23 (SI). First, the *K*_SC_ values of SC against methyl esters were in the range of 7–28 M^−1^; small but gradual increases in *K*_SC_ were observed as the number of carboxylic groups increased. The *K*_SC_ values for TT and TC were similar, indicating that the contribution of the benzene ring (π–π interaction) in supramolecular complexation was minimal. This reinforced the hydrogen bonding interactions as the main driving force. Subsequently, the *K*_SC_ values of SC against anions were between 850 and 1940 M^−1^. These values exhibited a factor of 44–121 enhancement compared with the corresponding methyl esters. The marginal deviation in *K*_SC_ as the number of carboxylates increased from two to three was likely owing to the bulkiness of the TBP cation (steric hindrance). Similar *K*_SC_ values for TBPT and TBPC were observed, showing that the main complexation was derived from hydrogen bonding interactions and not π–π interactions.

**Table 1:** Binding constants (*K*) between guest molecules and ref, SC, or SC·(sumanene)_n_ in CH_2_Cl_2_ at 25 °C.

Guest	*K*/M^−1^			
	ref (*K*_ref_)	SC (*K*_SC_)	SC·(sumanene)_n_ (*K*_SMP_)	*K*_SMP_/*K*_SC_
MB	4 ± 1	7 ± 1	79 ± 6	11.3
DI	*a*	16 ± 2	46 ± 16	2.9
TT	*a*	20 ± 2	78 ± 32	3.9
TC	*a*	28 ± 8	102 ± 28	3.6
TBPB	4 ± 1	850 ± 10	2120 ± 310	2.5
TBPI	*a*	1940 ± 140	3140 ± 680	1.6
TBPT	*a*	1400 ± 90	1470 ± 100	1.1
TBPC	*a*	1240 ± 80	1310 ± 110	1.1
testosterone	*a*	34 ± 3	170 ± 17	5.0
corticosterone	*a*	440 ± 10	530 ± 50	1.2
allylestrenol	*a*	4 ± 0.3	250 ± 20	62.5

[ref] = 1.35–1.36 mM, [SC] = 396–480 μM, [SC·(sumanene)_n_] = 394–450 μM SC + 8.64–9.29 mM sumanene; DP = 3.2–3.3

^a^Not determined.

### Hetero-supramolecular polymerization consisting of SC and sumanene

**Figure 4:**
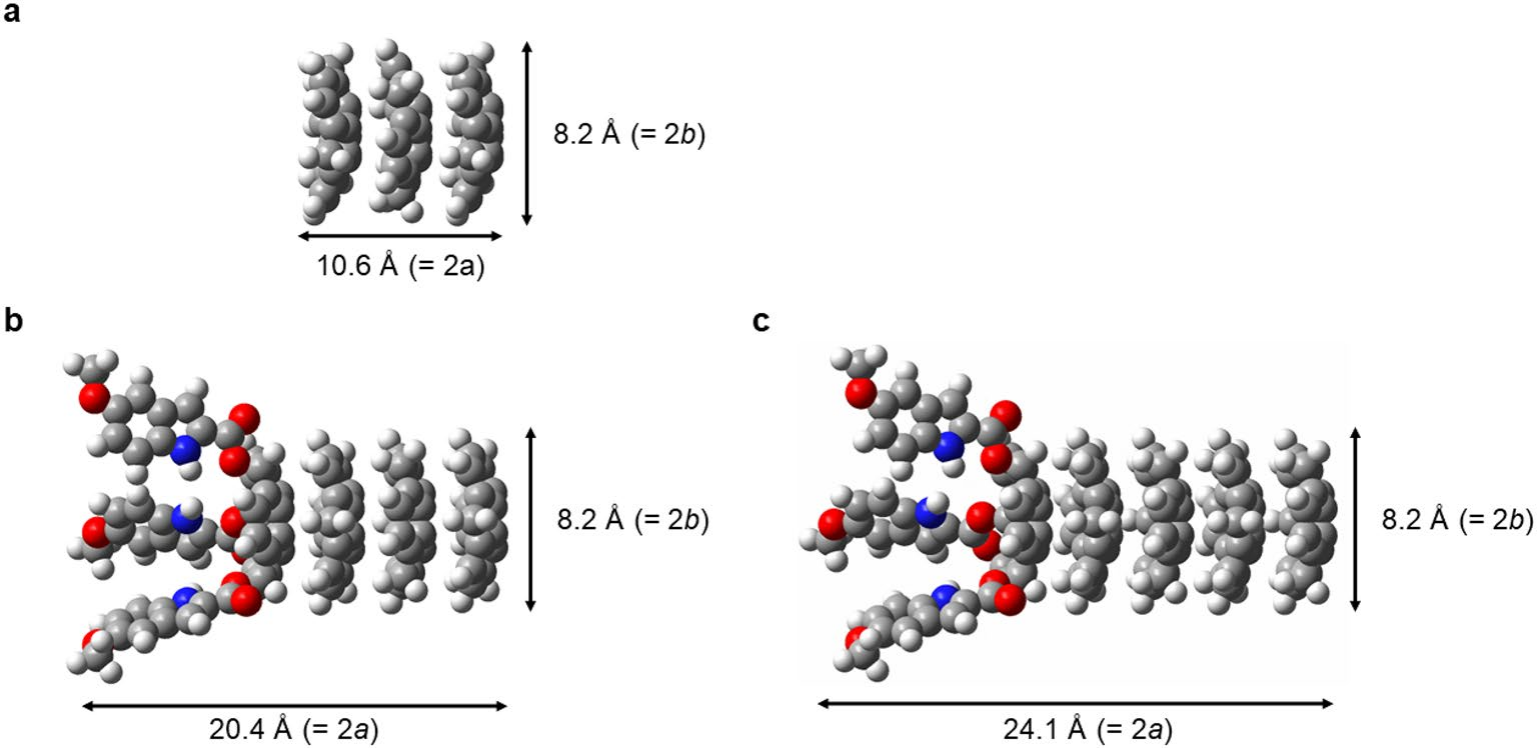
Optimized structures of (**a**) (sumanene)_3_, (**b**) SC·(sumanene)_3_, and (**c**) SC·(sumanene)_4_; *a* is long axis of ellipsoid and *b* is short axis of ellipsoid.

Similar to a previous study on homo-supramolecular polymerization^46^, the formation of hetero-supramolecular polymers using SC and sumanene in CH_2_Cl_2_ was investigated. No new absorption bands were observed in the UV spectra as sumanene was titrated against 128 μM of SC (Fig. S25 in SI); these UV spectra were similar to the concentration-dependent UV spectra of sumanene. Nevertheless, the hetero-supramolecular polymerization behavior was elucidated by calculating the molar extinction coefficient of the sumanene skeleton in SC, *ε*_sumanene,(calcd.)_. This provided the basis for the $\overline{\varepsilon }$ value of the SC·(sumanene)_n_ hetero-supramolecular polymer, which was used to estimate *α*_agg_ (Fig. S25, Table S5, and their relevant discussion in SI). Assuming that the model was isodesmic based on the similar curvature surfaces of SC (depth as 0.90 Å) and sumanene (depth as 0.89 Å) estimated using the DFT calculations, a *K*_i_ value of 770 ± 120 M^−1^ was determined after analyzing the molar extinction coefficient at 363 nm. Heteromer formation was further supported by the diffusion coefficient (*D*) obtained via NMR diffusion ordered spectroscopy (DOSY). The *D* value of a CD_2_Cl_2_ mixture of SC (447 μM) and sumanene (9.09 mM) was 7.37 × 10^–10^ m^2^/s (Figs. S26–S29 in SI). This value was subjected to the ellipsoid approximation model; a 4–5-mer was estimated (Table S6 and its relevant discussion in SI). The obtained *D* values clearly support the formation of the heteromer, not monomer. By contrast, the number-average DP value of the heteromer calculated using the assumption of the isodesmic model was 3.3, which was similar to the DOSY experiment (see structures in Fig. 4). Here, we adopted that SC functions as a chain capper, since all the indole moieties in SC stably align in an endo-form manner (see Fig. 2d), which hampers a random copolymerization in the middle of SC insertion. The further DFT calculation of trihydroxysumanene as a starting material of SC also supports that the endo-form is more stable in 18.4 kJ mol^−1^ than the exo-form, reinforcing the validity of the heteromer structure.

### Amplification-sensing behavior using the hetero-supramolecular polymer

**Figure 5:**
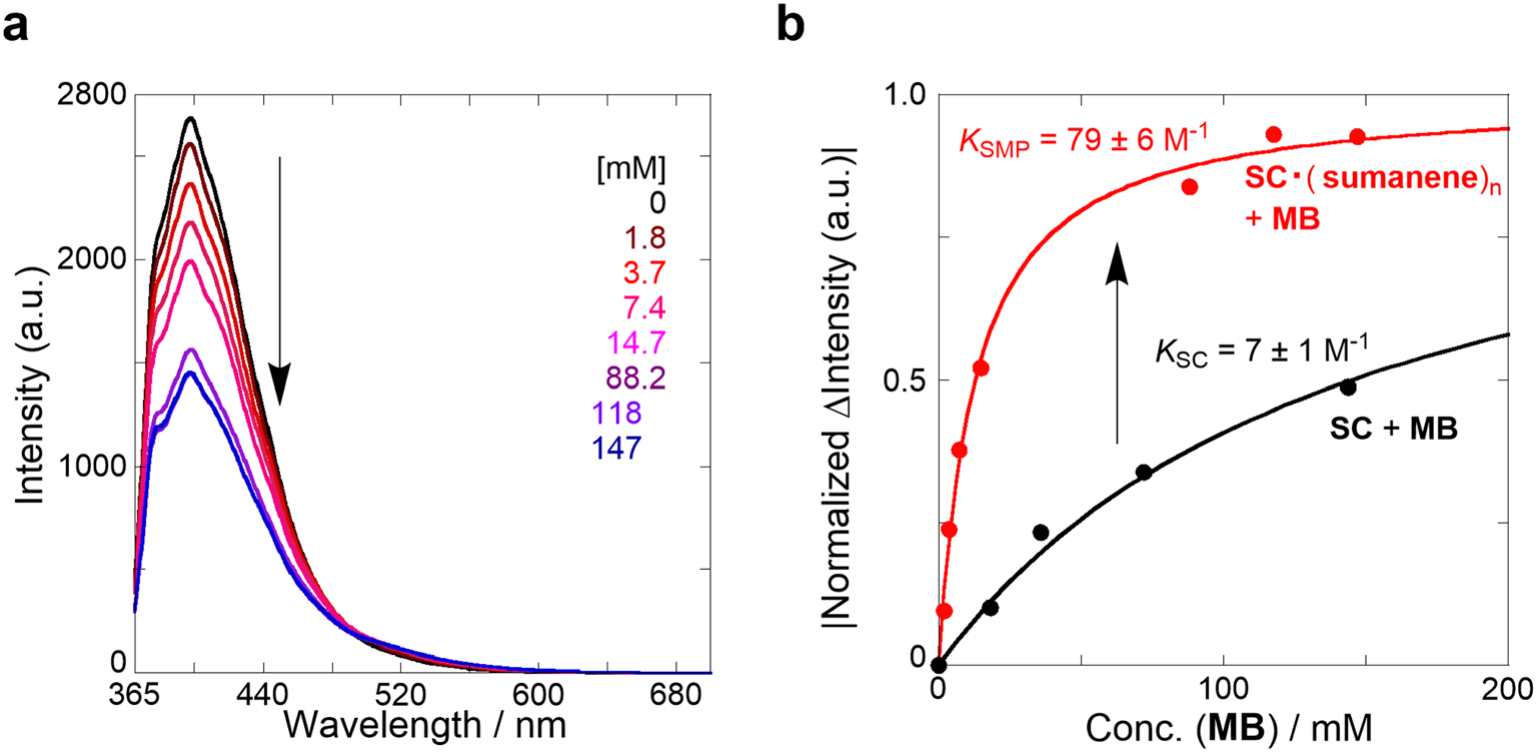
Sensing behavior of SC·(sumanene)_n_ hetero-supramolecular polymer. (**a**) Fluorescence spectra (λ_ex_: 355 nm) of SC (445 μM) with sumanene (8.87 mM, DP = 3.2, black) following the addition of MB (1.8–147 mM, from brown to blue) in CH_2_Cl_2_ at 25 °C, measured in a 1 mm cell; the excitation wavelength at which comparable absorbances were obtained was selected. (**b**) Normalized binding isotherms of SC·(sumanene)_n_ (red) and SC (black) following the addition of MB at 25 °C.

Broad peaks were observed in the long wavelength region of the UV/vis absorption spectra of SC·(sumanene)_n_ ⊂ MB in CH_2_Cl_2_ (Fig. S34a in SI), which was similar to the peaks in the long wavelength region of the SC ⊂ MB spectra. A fluorescence quench was observed in the fluorescence spectra of SC·(sumanene)_n_ ⊂ MB (Fig. 5a); this quench was similar to that observed in the SC ⊂ MB spectra. A new emission band was observed at the longer wavelength region (approximately 500 nm) in the normalized fluorescence spectra (Fig. S34b in SI), indicating that supramolecular complexation of SC·(sumanene)_n_ ⊂ MB occurred. The fluorescence spectral changes at 399 nm were fitted (Fig. 5b, red), assuming that the hetero-supramolecular polymer and the guest molecule form a 1:1 complex owing to the same recognition moiety (the SC starburst in the complex). The obtained apparent binding constant (*K*_SMP_) was 79 ± 6 M^−1^, which was 11.3-fold higher than the *K*_SC_ of SC ⊂ MB (7 ± 1 M^−1^).

Table 1 shows the *K*_SMP_ and *K*_SC_ values of the eight guest molecules and the sensing behaviors of the hetero-supramolecular polymer and SC system. Large amplification ratios of 2.9–11.3 for esters with smaller *K*_SC_ were observed, whereas amplification ratios of 1.1–2.5 for anions with relatively large *K*_SC_ (Figs. S35–S48 in SI). Therefore, signal-amplification sensing with sumanene supramolecular polymers may be useful for enhancing a low signal of a target molecule with a small *K* in the SC system.

**Figure 6:**
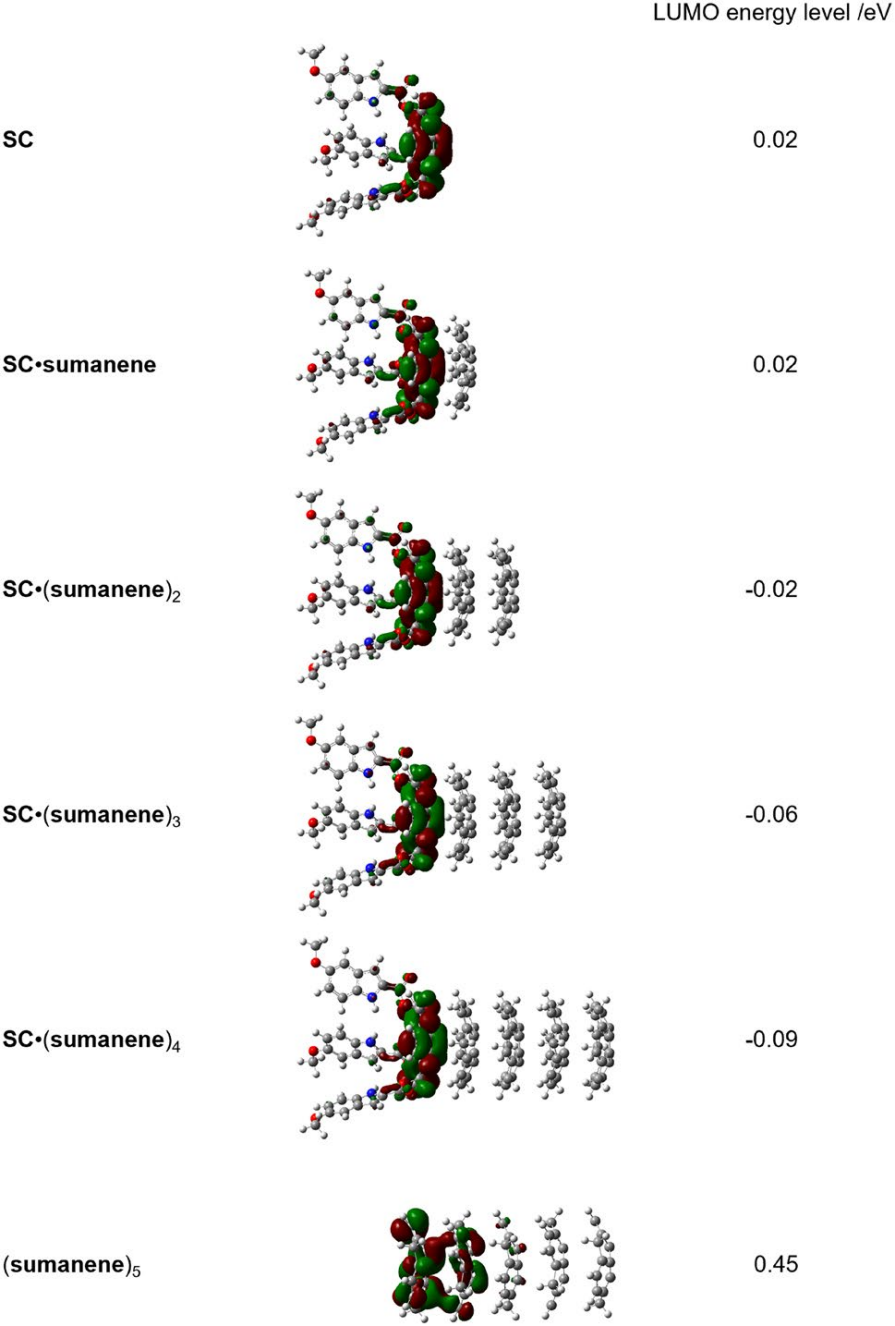
LUMO and the corresponding energy levels calculated from the optimized structures of SC, SC·(sumanene)_n_ (n = 1–4), and (sumanene)_5_.

To elucidate the origin of the signal-amplification, DFT calculations (function/basis set: ωB97X-D/6-311G(d,p)) of SC, sumanene, and SC·(sumanene)_n_ were performed (Fig. 6). The LUMO energy of SC was 0.02 eV; this value remained constant even when a sumanene molecule stacked on SC (dimer formation). By contrast, stacking two or more sumanene molecules on SC resulted in the gradual reduction of LUMO energies to between -0.02 (SC·(sumanene)_2_) and -0.09 eV (SC·(sumanene)_4_). Furthermore, the LUMO energy of (sumanene)_5_ was positive (0.45 eV). Therefore, the likelihood of the negative LUMO energy values being more electron-receptive was high^50^. This improved the acceptor properties of the SC binding site in the heteromer. Because the LUMO orbitals of SC·(sumanene)_n_ extended from the sumanene core in SC to the indole binding site, the formation of SC·(sumanene)_n_ influenced the electronic acceptor properties of the indole moiety—the origin of the signal-amplification. In addition, natural population analysis showed that the formation of hetero-supramolecular polymers caused the electron transfer from sumanene to SC, resulting in an anionic SC core in the charge-transfer (CT) complex (Table S7 in SI). Therefore, the signals for the anions were not amplified because of electrostatic repulsion in the hetero-supramolecular polymer system; however, amplification was observed against the esters.

**Figure 7:**
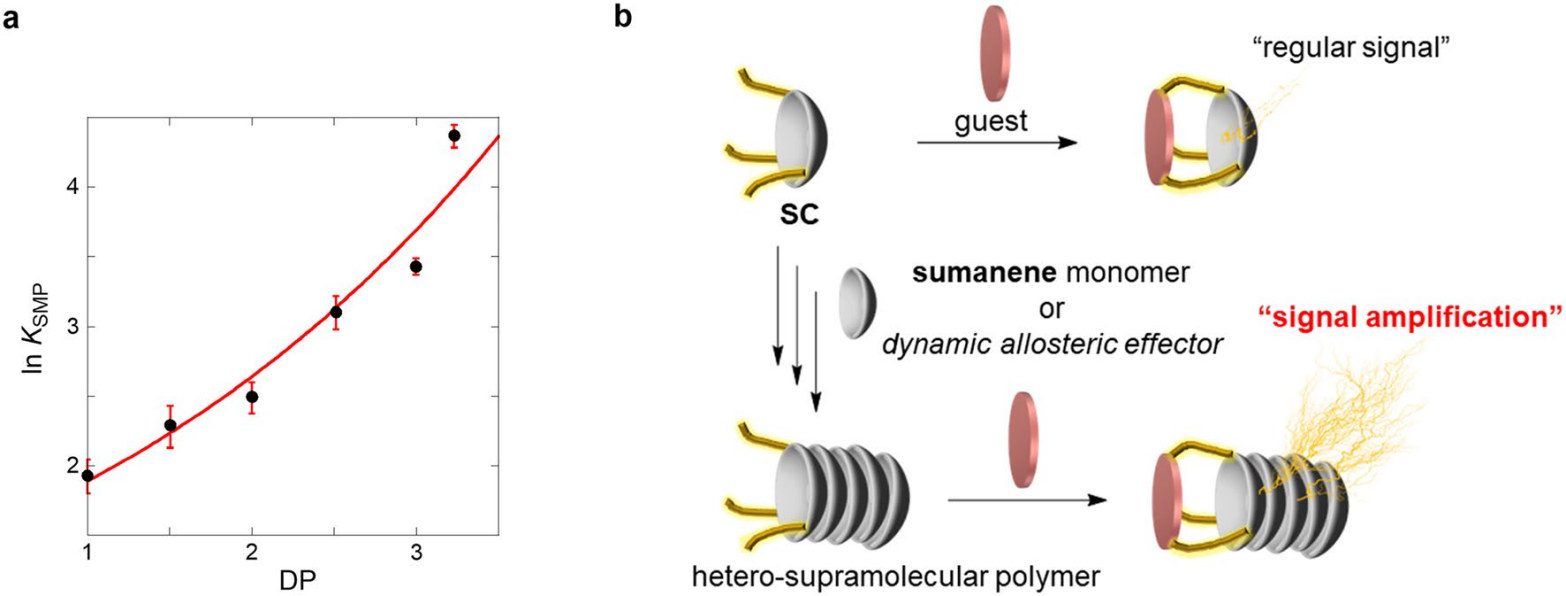
Dynamic allosteric behavior of SC·(sumanene)_n_ hetero-supramolecular polymer. (**a**) Plots of ln *K*_SMP_ between SC·(sumanene)_n_ (n = 0 ~) and MB at 25 °C as a function of DP; the red line represents a fitting regression line (*y* = 1.3498 e^0.33547x^, *r* = 0.970). (**b**) Conceptual illustration of “regular signal” (the common method) *vs.* “signal amplification” via a dynamic allosteric effector (this study).

Importantly, the experimental and theoretical findings showed that the number of stacks of sumanene played a critical role in signal amplification. The functionality of the conceptual mechanism shown in Fig. 1b was assessed by sensing MB with varying concentrations of the sumanene monomer in SC (Figs. S30–S33 in SI). The ln *K*_SMP_ value (= Δ*G*°) as a function of DP increased exponentially (Fig. 7a); therefore, the behavior of sumanene was that of a *dynamic allosteric effector*. The conceptual illustration observed in this study (signal amplification) is shown in Fig. 7b.

### Steroid sensing: general validity and application using the hetero-supramolecular polymer

**Figure 8:**
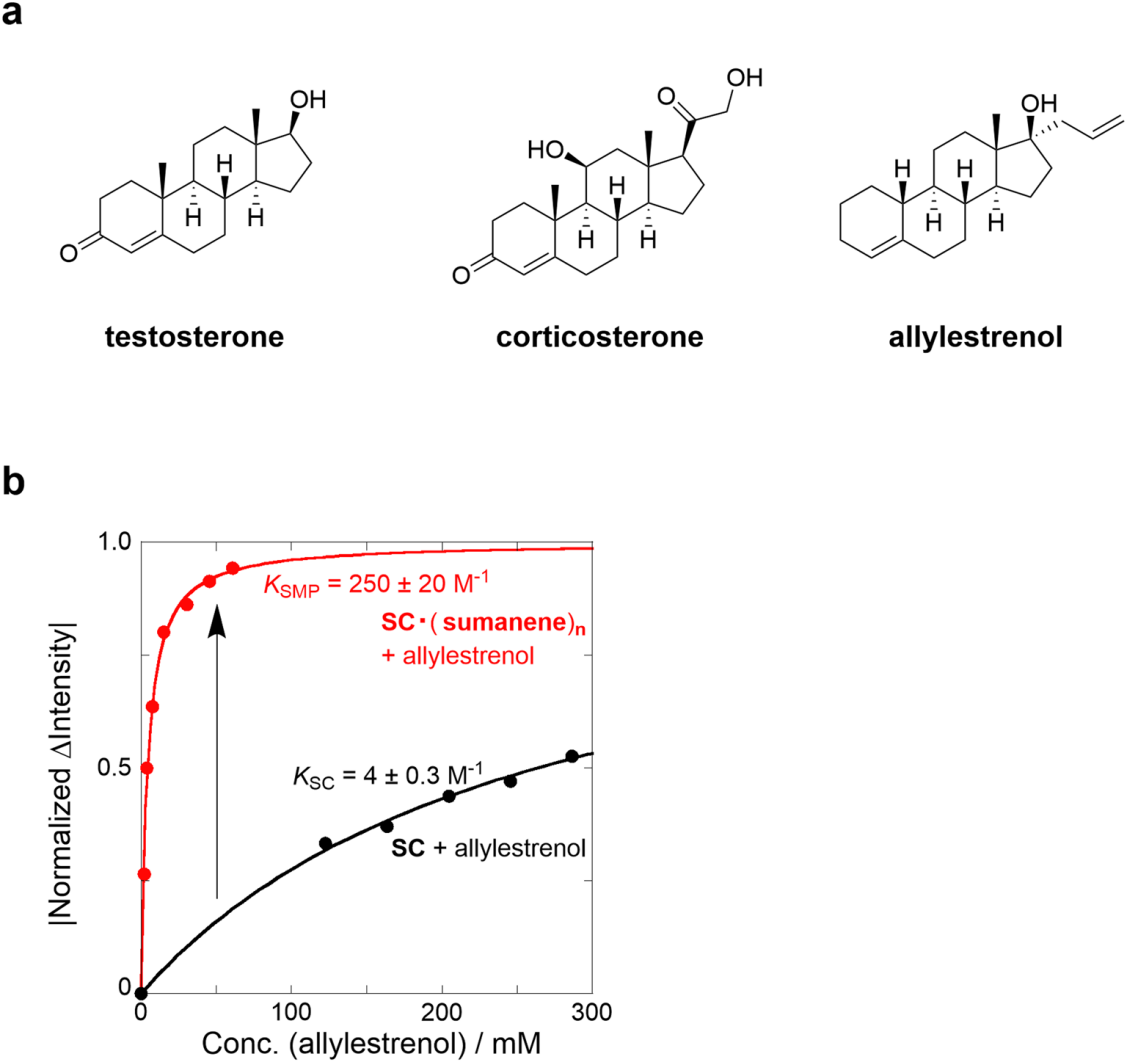
Steroid sensing behavior of SC·(sumanene)_n_ hetero-supramolecular polymer. (**a**) Chemical structures of steroids. (**b**) Normalized binding isotherms of SC·(sumanene)_n_ (red) and SC (black) upon the addition of allylestrenol at 25 °C.

To further generalize the current signal-amplification method and demonstrate its applicability in biologically important materials, steroids such as testosterone, corticosterone, and allylestrenol were selected as target molecules with lower donor characteristics (Fig. 8a). The fluorescence spectral changes of SC·(sumanene)_n_ ⊂ allylstrenol exhibited distinctive fluorescence quenching similar to that of other sensing systems (Figs. S49–S53 in SI). A similar 1:1 fitting for the fluorescence changes at 409 nm resulted in a *K*_SMP_ value of 250 ± 20 M^−1^, which was a 62.5-fold higher signal-amplification than *K*_SC_ of SC ⊂ allylstrenol as 4 ± 0.3 M^−1^ (Fig. 8b). A lower *K*_SC_ value in the SC system corresponded to a higher amplification of the *K*_SMP_ in the hetero-supramolecular polymer system. It was concluded that the signal amplification by the sumanene-based hetero-supramolecular polymer was 62.5-fold higher than that of the other sensing systems.

## Conclusion

A novel signal-amplification system was developed, wherein the curved-π sumanene monomer for supramolecular polymerization functioned as a dynamic allosteric effector. This monomer effector altered the DP to flexibly manipulate the electronic properties at the binding site (positive heterotopic allosterism), achieving an amplification that was up to 62.5-fold higher than other systems at sensing the biologically important steroid, allylestrenol. The sensing method and the conceptual guideline proposed herein facilitate the sensing of diverse guests that are difficult to signally recognize using the conventional lock-and-key type chemosensors.

## Methods

### Complexation studies

A stock solution of SC was prepared by dissolving SC powder in CH_2_Cl_2,_ followed by sonication for 1 min. Unused stock solutions were stored in the freezer. Stored samples were warmed to room temperature and then sonicated for 1 min before use. The concentration was calculated from the absorbance of the solution. The CH_2_Cl_2_ mixture of SC and sumanene was prepared by mixing ca. 600 μM SC solution and ca. 2–34 mM sumanene solution in a ratio of 8:3. The CH_2_Cl_2_ solutions of the guest molecules were prepared at 50 mM to 8 M concentrations; thereafter, these solutions were titrated into SC or hetero-supramolecular polymer solutions using a micro-syringe injection. A preparation concentration of SC was used for the nonlinear least-squares fitting to determine the binding constants. IR spectra were measured on the plate by adding a few drops of CH_2_Cl_2_ solution to the sample and then drying.

### Computational studies

All chemosensors and supramolecular complexes, except for trihydroxysumanene, were structurally optimized by the DFT calculations with the ωB97X-D/6-311G(d,p) level, and the most stable structures were used for discussions; the calculations for trihydroxysumanene were performed with the M06-2X/6-311++G(d,p) level, according to the results of a similar analog, monohydoxysumanene^51^. Triethylene glycol chains in SC were changed to methoxy groups for simplicity.

### Supplementary Information

Supplementary Information.

## Data Availability

All results and supporting data are available within the main text and Supplementary Information. They can also be requested from the corresponding author.
