# Supplementary material for: Amplification sensing manipulated by a sumanene-based supramolecular polymer as a dynamic allosteric effector
Source: Sci Rep. 2024 May 31;14:12534. doi: 10.1038/s41598-024-63304-4 (PMC11143208; doi:10.1038/s41598-024-63304-4)
Supplement: Supplementary file 1 — Supplementary Information. [file 41598_2024_63304_MOESM1_ESM.docx]

*Supplementary Information*

***for***

Amplification Sensing Manipulated by a Sumanene-Based Supramolecular Polymer as a Dynamic Allosteric Effector

**Hiroaki Mizuno,^†^ Hironobu Nakazawa,^‡^ Akihisa Miyagawa,^§^ Yumi Yakiyama,^‡,¶^ Hidehiro Sakurai^*,‡,¶^ and Gaku Fukuhara^*,†^**

^†^ Department of Chemistry, Tokyo Institute of Technology, 2-12-1 Ookayama, Meguro-ku, Tokyo 152-8551, Japan

^‡^ Division of Applied Chemistry, Graduate School of Engineering, Osaka University, Suita, Osaka 565-0871, Japan

^§^ Department of Chemistry, Faculty of Pure and Applied Sciences, University of Tsukuba, Tsukuba, Ibaraki 305-8577, Japan

^¶^ Division of Applied Chemistry, Graduate School of Engineering and Innovative Catalysis Science Division, Institute for Open and Transdisciplinary Research Initiatives (ICS-OTRI), Osaka University, Suita, Osaka 565-0871, Japan

E-mail: hsakurai@chem.eng.osaka-u.ac.jp (H.S.); gaku@chem.titech.ac.jp (G.F.)

**Experimental Section**

**Materials.** All commercial reagents and solvents were used without further purification. Fluorescence-free grade CH_2_Cl_2_ was used as received for spectroscopy. **Sumanene** and model guests (**MB**, **DI**, **TT**, **TC**, **TBPB**, and **TBPI**) were synthesized, according to the literature.^[[1]](#footnote-2)^

**Instruments.** Melting points were measured with a Büchi apparatus. High-resolution mass spectrometry (HR-MS) was recoded on a micrOTOF Ⅱ. ^1^H (400 and 500 MHz) and ^13^C NMR (100 and 125 MHz) spectra were measured on an ECX400, ECX500, or ECZ400S. UV/vis absorption spectra were measured by a JASCO V-650 equipped with temperature controllers. Fluorescence spectra were measured by a JASCO FP-8500 equipped with a temperature controller. Fluorescence lifetime decays were measured by a Hamamatsu Quantaurus-Tau single photon counting apparatus fitted with an LED light source. IR spectra were measured by a JASCO FT/IR-4700 instrument. All spectroscopic measurements except for IR measurements were measured in a quartz cell of 1 mm or 1 cm optical pass length.

**Fluorescence measurements.** In order to prevent a self-quenching effect, a 1 mm quartz cell was set at 55° against the excitation light (**Figure S1**).

**
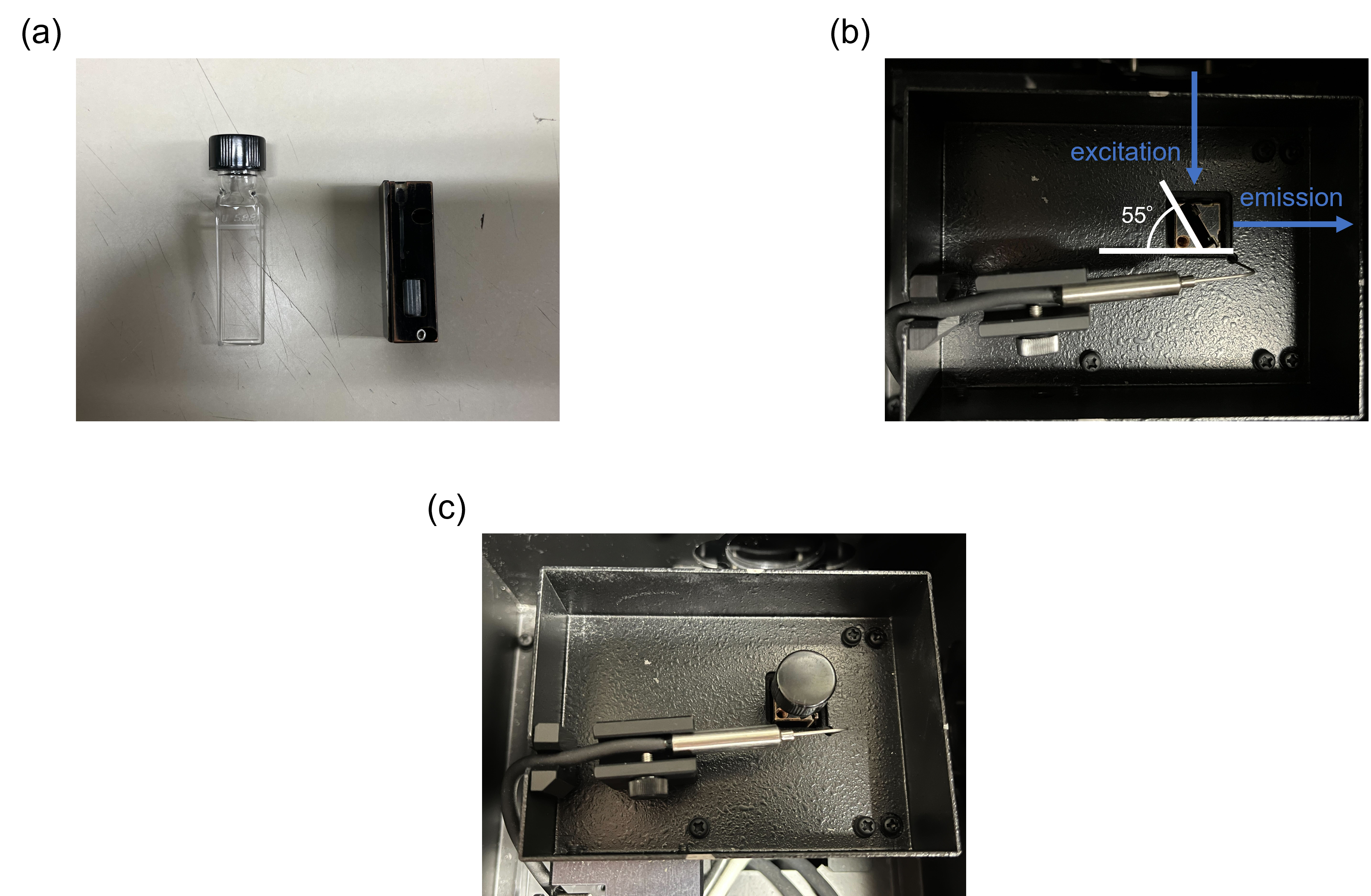
**

**Figure S1.** Images of (a) a 1 mm cell and an adopter for the quartz cell and (b,c) set up for the fluorescence measurements using a 1 mm cell adopter.

**Synthesis and Characterization of SC, ref, TBPT, and TBPC**

**
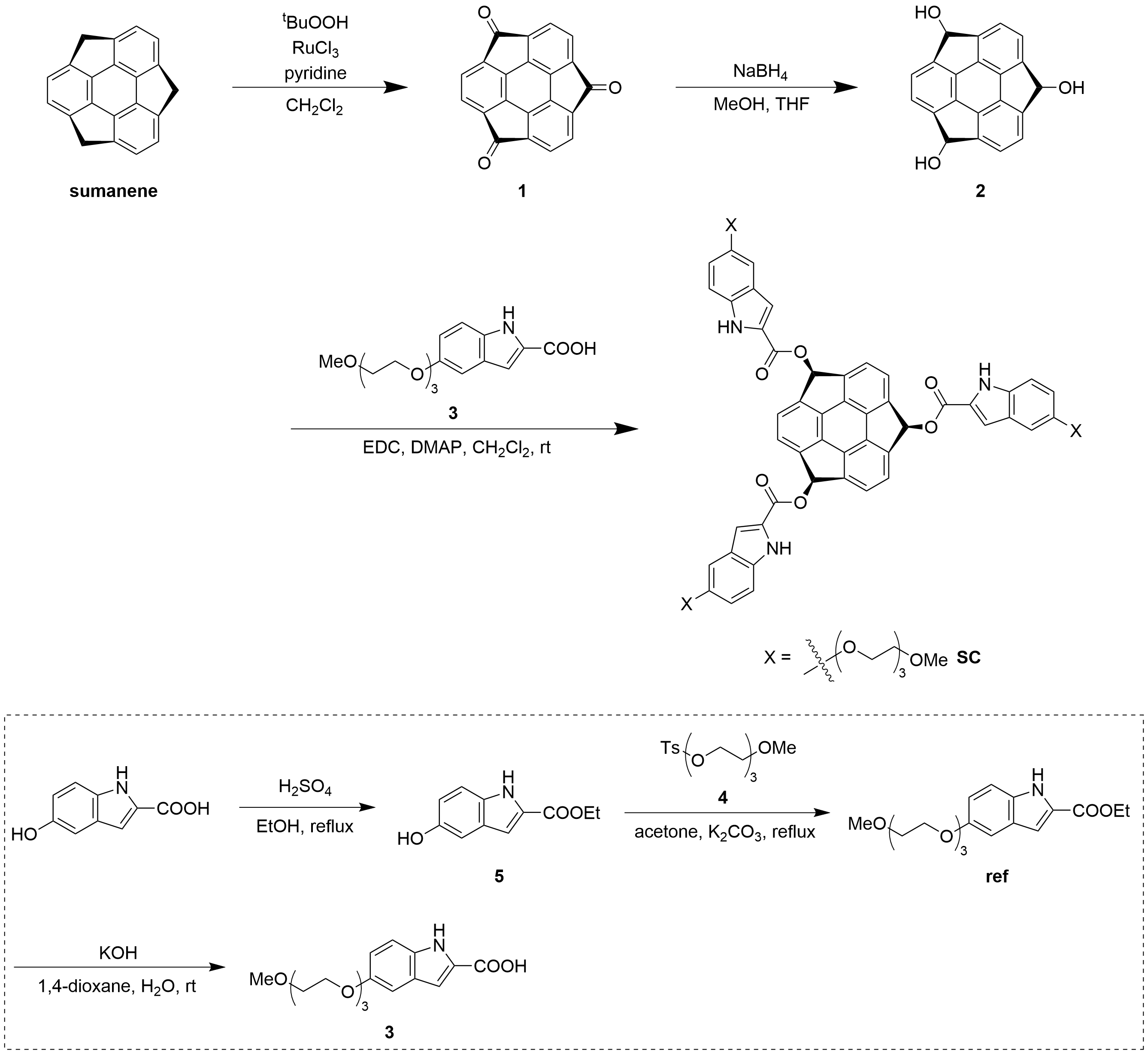
**

**Figure S2.** Synthetic scheme of **SC** and **ref**.


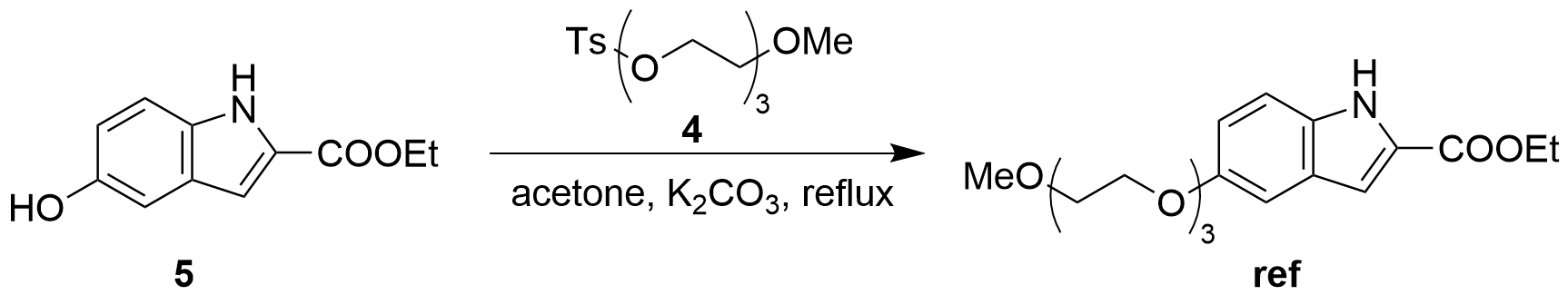


**Ethyl 5-(2-(2-(2-methoxyethoxy)ethoxy)ethoxy)-1*H*-indole-2-carboxylate (ref).** **4**^[[2]](#footnote-3)^ (210 mg, 661 μmol), **5**^[[3]](#footnote-4)^ (200 mg, 973 μmol), and K_2_CO_3_ (886 mg, 6.41 mmol) were dissolved in acetone (25 mL) under N_2_ atmosphere and refluxed for 4 d. The mixture was extracted with brine and ethyl acetate (EtOAc). The organic layer was dried over anhydrous Na_2_SO_4_ and filtered. After evaporating, the resultant was purified with silica gel flash column chromatography (2:3 EtOAc/hexane) to obtain **ref** as pale yellow oil (218 mg, 621 μmol) in 94% yield. HR-MS (ESI, TOF): *m*/*z* 374.1583 ([M+Na]^+^, C_18_H_25_NNaO_6_, calcd. 374.1574); ^1^H NMR (CDCl_3_, 400 MHz, rt) δ_H_ 8.99 (s, 1H), 7.31 (d, *J* = 0.9 Hz, 1H), 7.13 (d, *J* = 1.8 Hz, 1H), 7.08 (d, *J* = 2.3 Hz, 1H), 7.02 (dd, *J* = 2.2, 9.2 Hz, 1H), 4.40 (q, *J* = 7.3 Hz, 2H), 4.16 (t, *J* = 4.8 Hz, 2H), 3.88 (t, *J* = 4.8 Hz, 2H), 3.77-3.75 (m, 2H), 3.71-3.65 (m, 4H), 3.57-3.54 (m, 2H), 3.38 (s, 3H), 1.41 (t, *J* = 7.3 Hz, 3H); ^13^C NMR (CDCl_3_, 100 MHz, rt) δ_c_ 161.9, 153.9, 132.3, 127.9, 127.8, 117.5, 112.7, 108.2, 103.9, 72.0, 70.8, 70.7, 70.6, 69.9, 68.0, 61.0, 59.1, 14.4.


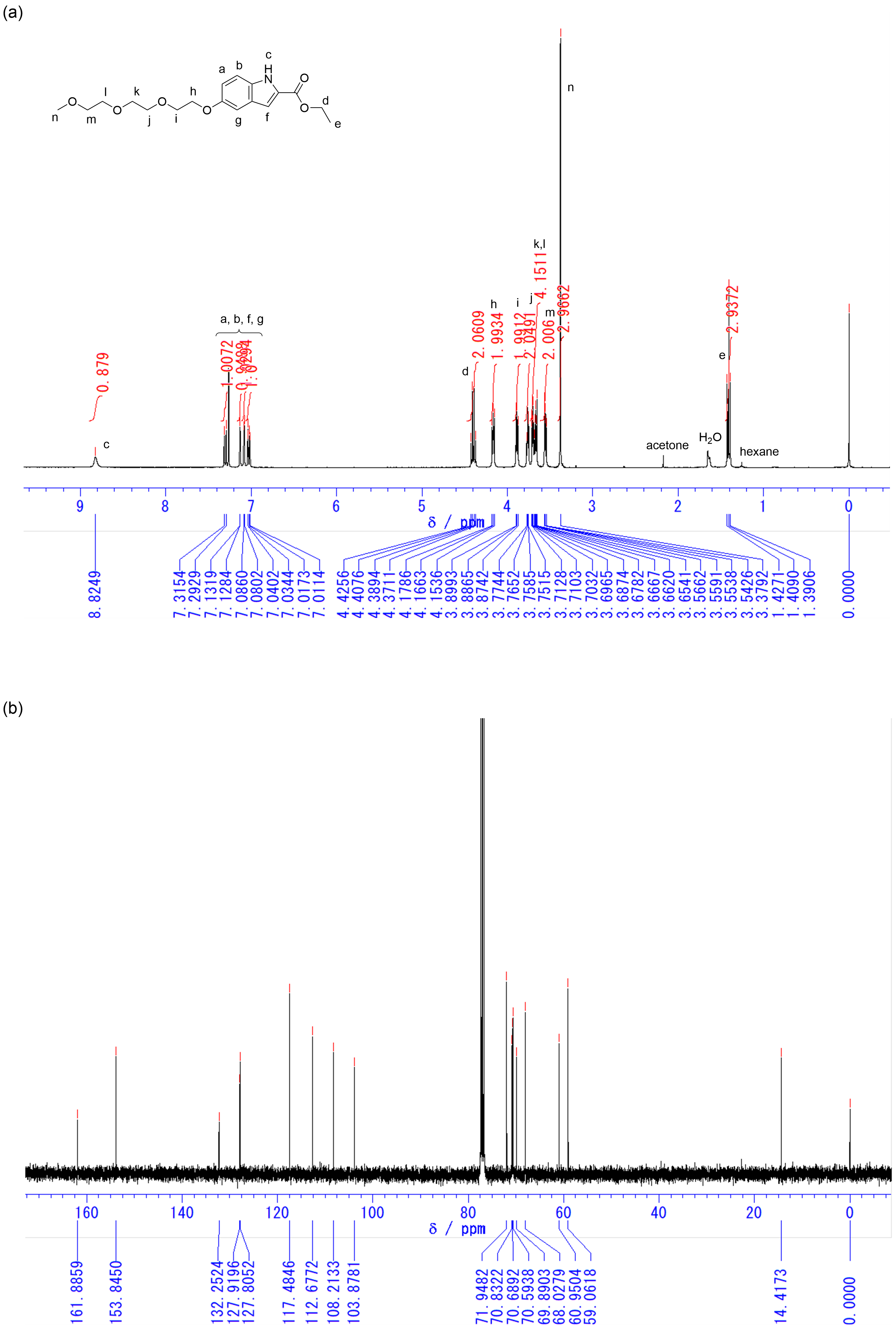


**Figure S3.** (a) ^1^H and (b) ^13^C NMR spectra of **ref** in CDCl_3_ at room temperature.


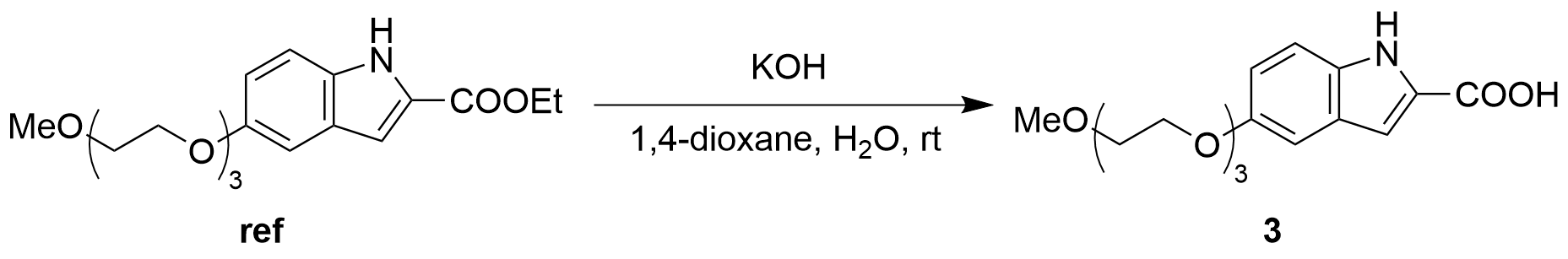


**5-(2-(2-(2-Methoxyethoxy)ethoxy)ethoxy)-1*H*-indole-2-carboxylic acid (3).** The **ref** compound (218 mg, 621 μmol) was dissolved in 1,4-dioxane (7 mL). An aqueous solution of 1.1 M KOH (2.1 mL) was added to the solution and stirred for 46.5 h. After the reaction was completed, 1 M HCl aq. (4 mL) was added to the solution to acidify and then stirred for 2.5 h. The mixture was extracted with brine and CH_2_Cl_2_. The organic layer was dried over anhydrous MgSO_4_ and filtered. The filtrate was evaporated to obtain **3** as a pale yellow solid (215 mg, 664 μmol) in a quantitative yield. HR-MS (ESI, TOF): *m*/*z* 374.1270 ([M+Na]^+^, C_16_H_21_NNaO_6_, calcd. 346.1261); ^1^H NMR (CDCl_3_, 400 MHz, rt) δ_H_ 8.99 (br, 1H), 7.30 (d, *J* = 8.9 Hz, 1H), 7.22 (s, 1H), 7.06-7.02 (m, 2H), 4.16 (t, *J* = 5.0 Hz, 2H), 3.90 (t, *J* = 4.9 Hz, 2H), 3.79-3.77 (m, 2H), 3.73-3.67 (m, 4H), 3.58-3.56 (m, 2H), 3.39 (s, 3H); ^13^C NMR (CDCl_3_, 100 MHz, rt) δ_c_ 165.8, 153.9, 132.8, 127.8, 126.8, 118.3, 112.9, 110.0, 103.7, 71.9, 70.8, 70.7, 70.5, 69.9, 67.9, 59.0; m.p. 73 °C (dec.).


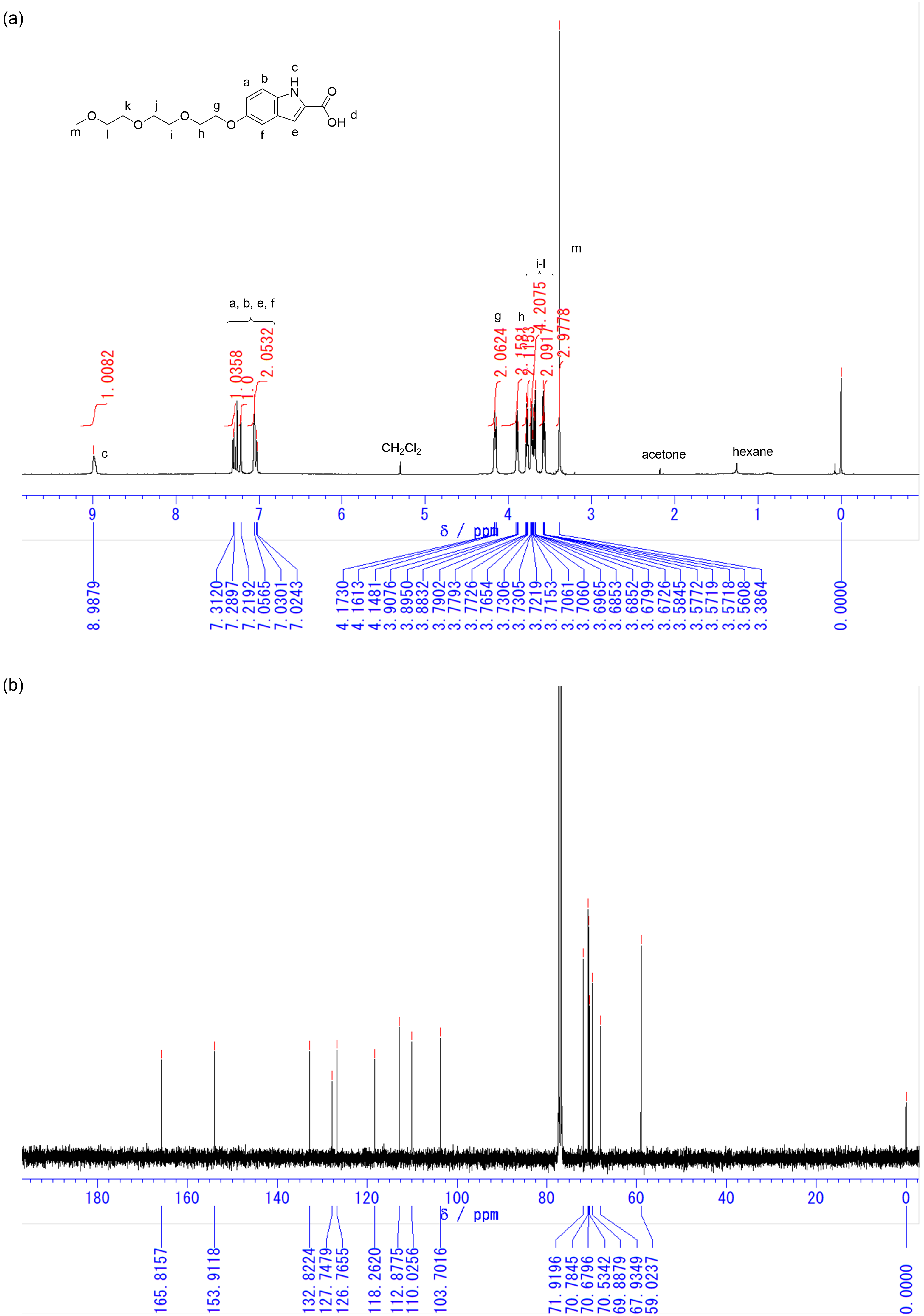


**Figure S4.** (a) ^1^H and (b) ^13^C NMR spectra of **3** in CDCl_3_ at room temperature.


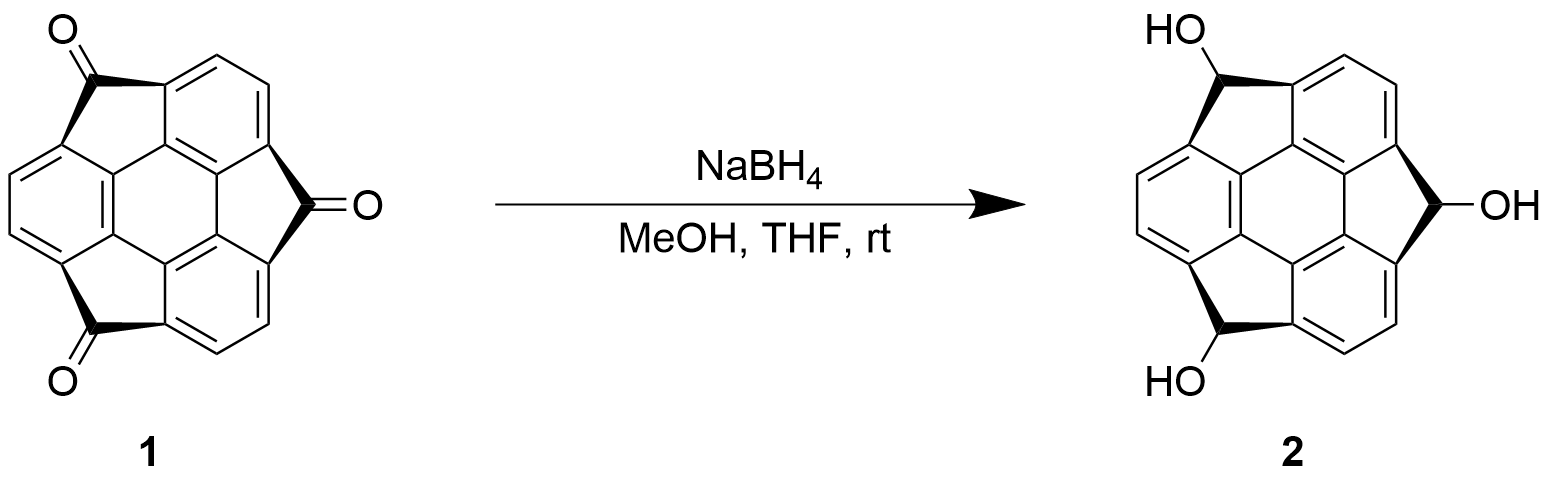


**Sumanenetriol (2). 1**^[[4]](#footnote-5)^ (30.4 mg, 99.3 μmol) was dissolved in dry CH_3_OH (11 mL) and dry THF (11 mL) under N_2_ atmosphere. NaBH_4_ (76 mg, 2.04 mmol) was added to the solution and then stirred for 5 min at room temperature. After evaporating, H_2_O, CHCl_3_, and 1 M HCl aq. were added and extracted. The organic layer was dried over Na_2_SO_4_ and filtered. After evaporating, the resultant was purified with preparative TLC (1:1 EtOAc/CHCl_3_) to obtain **2** as an orange solid (6.9 mg, 22.1 μmol) in 22% yield. HR-MS (EI): *m*/*z* 312.0784 ([M]^+^, C_21_H_12_O_3_, calcd. 312.0786); ^1^H NMR (CDCl_3_, 400 MHz, 40 °C) δ_H_ 7.15 (s, 6H), 6.80 (d, *J* = 10.3 Hz, 3H), 1.45 (d, *J* = 10.2 Hz, 3H); ^13^C NMR (CDCl_3_, 100 MHz, 40 °C) δ_c_ 157.0, 148.8, 121.7, 82.7; m.p. 286 °C (dec.).


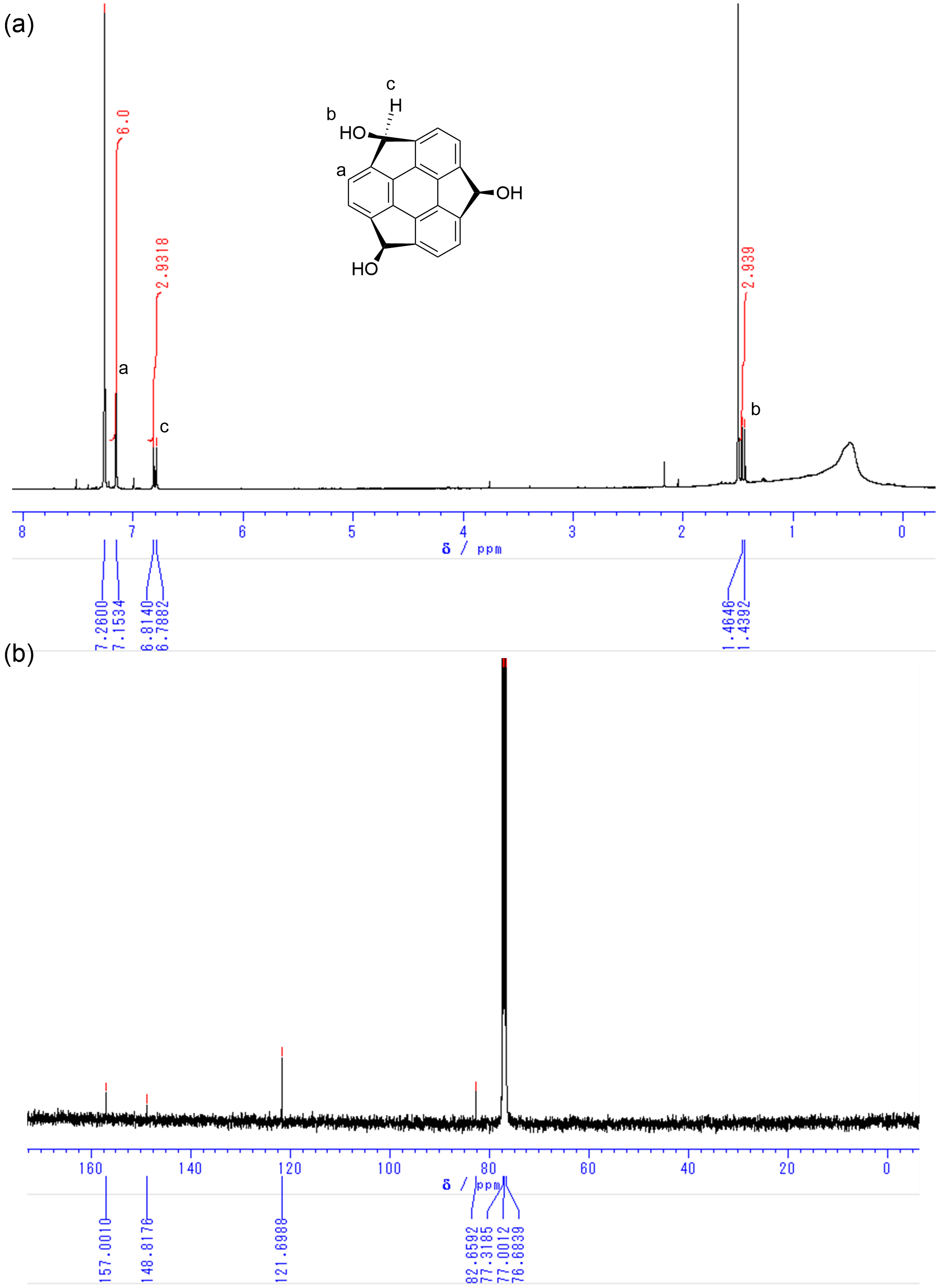


**Figure S5.** (a) ^1^H and (b) ^13^C NMR spectra of **2** in CDCl_3_ at 40 °C.


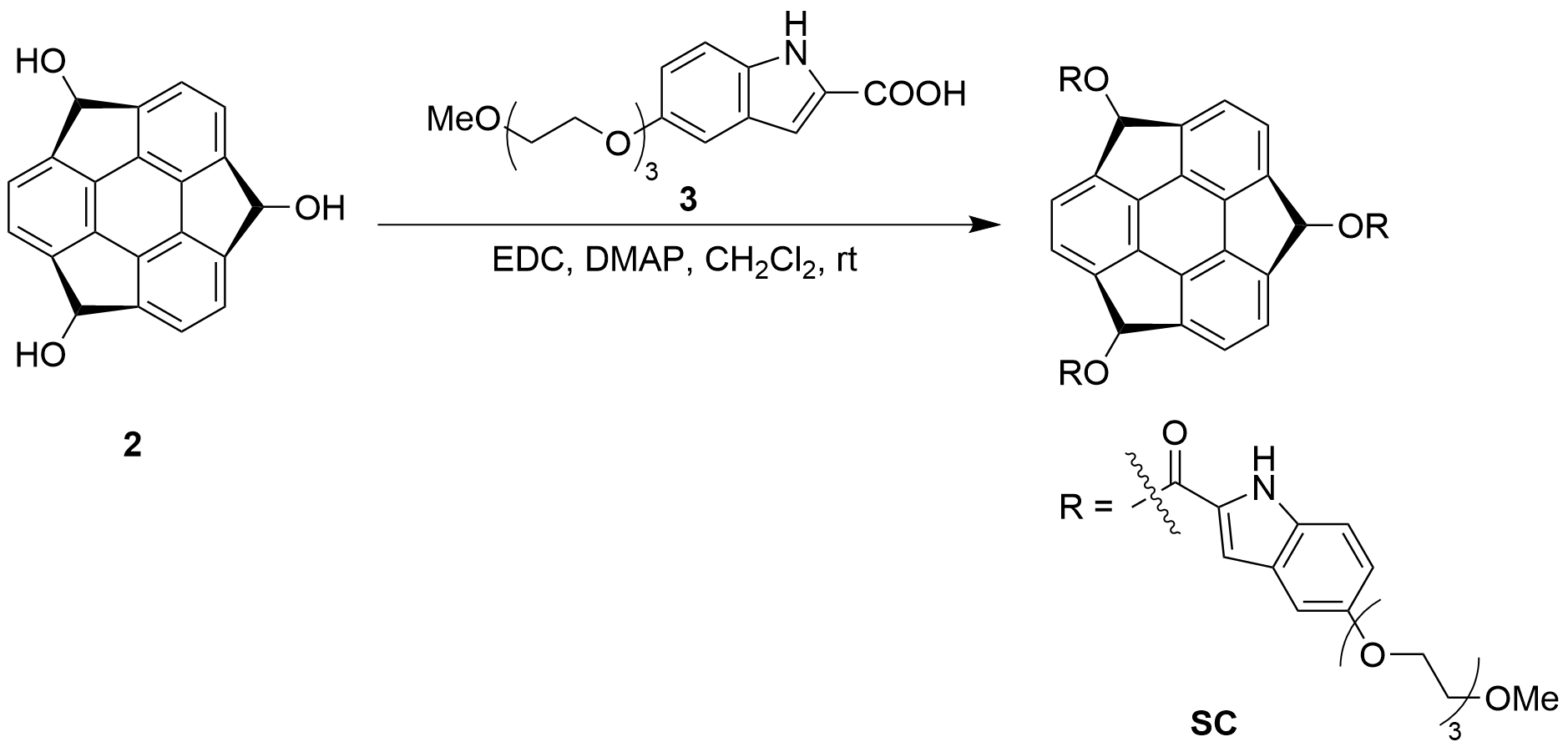


**SC.** **2** (11.0 mg, 35.2 μmol), **3** (171 mg, 528 μmol), 1-ethyl-3-(3-(dimethylamino)propyl)carbodiimide hydrochloride (EDC) (111 mg, 577 μmol), and *N*,*N*-dimethyl-4-aminopyridine (DMAP) (7.4 mg, 66.3 μmol) were dissolved in CH_2_Cl_2_ (2 mL) under N_2_ atmosphere and then stirred for 2 d at room temperature. The mixture was extracted with brine and CH_2_Cl_2_. The organic layer was dried over anhydrous Na_2_SO_4_ and filtered. After evaporating, the resultant mixture was purified with silica gel flash column chromatography (200:3 CH_2_Cl_2_/CH_3_OH). The residue was further purified by recrystallization (acetone/hexane or acetone/cyclohexane) to obtain **SC** as a white solid (5.94 mg, 52.6 μmol) in 14% yield. HR-MS (ESI, TOF): *m*/*z* 1250.4489 ([M+Na]^+^, C_69_H_69_N_3_NaO_18_, calcd. 1250.4468); ^1^H NMR (CDCl_3_, 400 MHz, rt) δ_H_ 8.74 (br, 3H), 8.02 (s, 3H), 7.26-7.24 (m, 3H), 7.13 (s, 6H), 7.07 (s, 3H), 7.02-6.99 (m, 6H), 4.11 (t, *J* = 4.8 Hz, 6H), 3.84 (t, *J* = 4.7 Hz, 6H), 3.74-3.71 (m, 6H), 3.68-3.62 (m, 12H), 3.53-3.51 (m, 6H), 3.34 (s, 9H); ^13^C NMR (CDCl_3_, 100 MHz, rt) δ_c_ 161.5, 154.0, 152.8, 149.2, 132.6, 127.6, 126.4, 122.9, 118.3, 112.8, 109.6, 103.7, 82.9, 71.9, 70.8, 70.6, 70.6, 69.8, 67.9, 59.0; m.p. 166 °C (dec.).


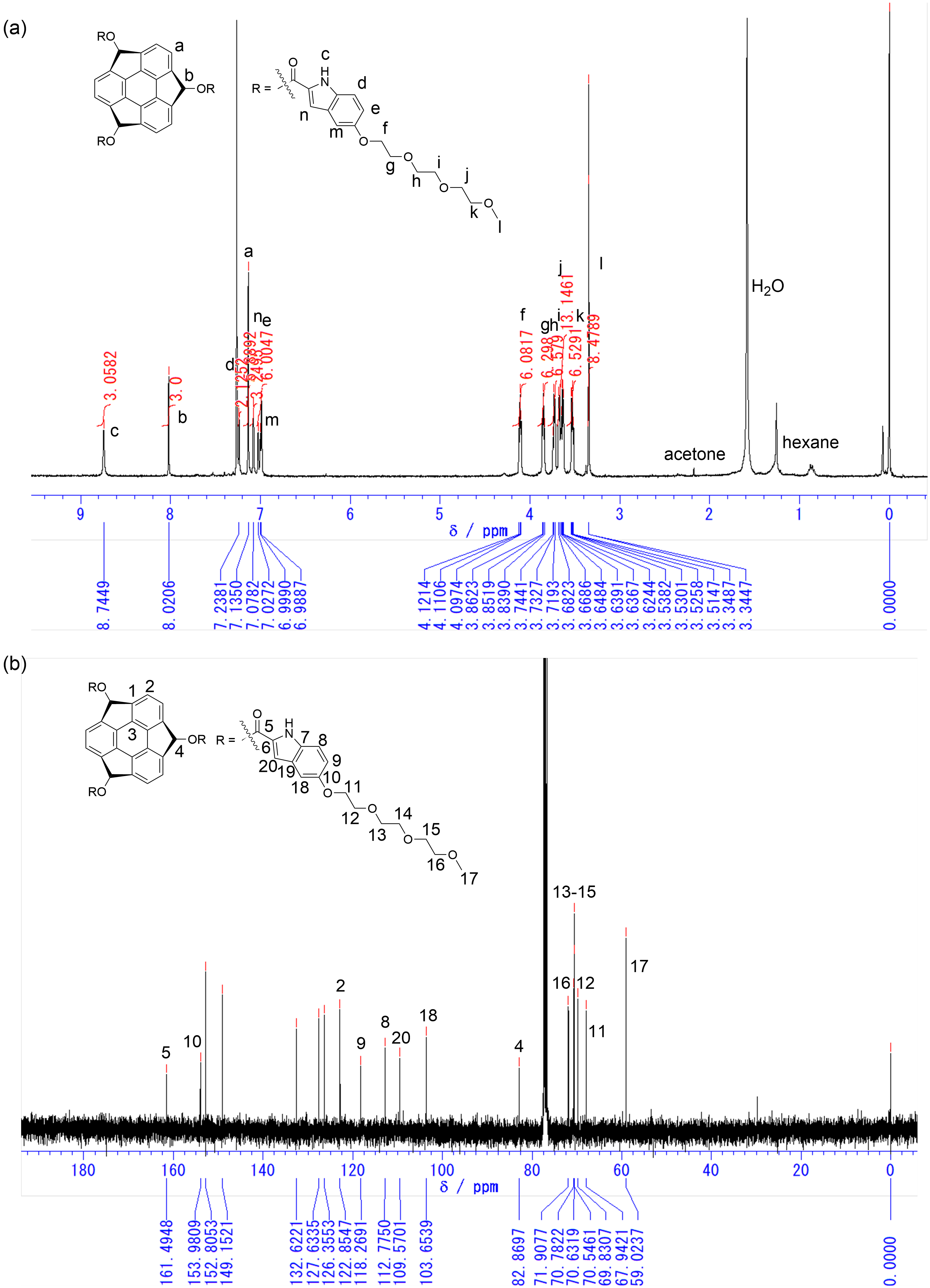


**Figure S6.** (a) ^1^H and (b) ^13^C NMR spectra of **SC** in CDCl_3_ at room temperature.


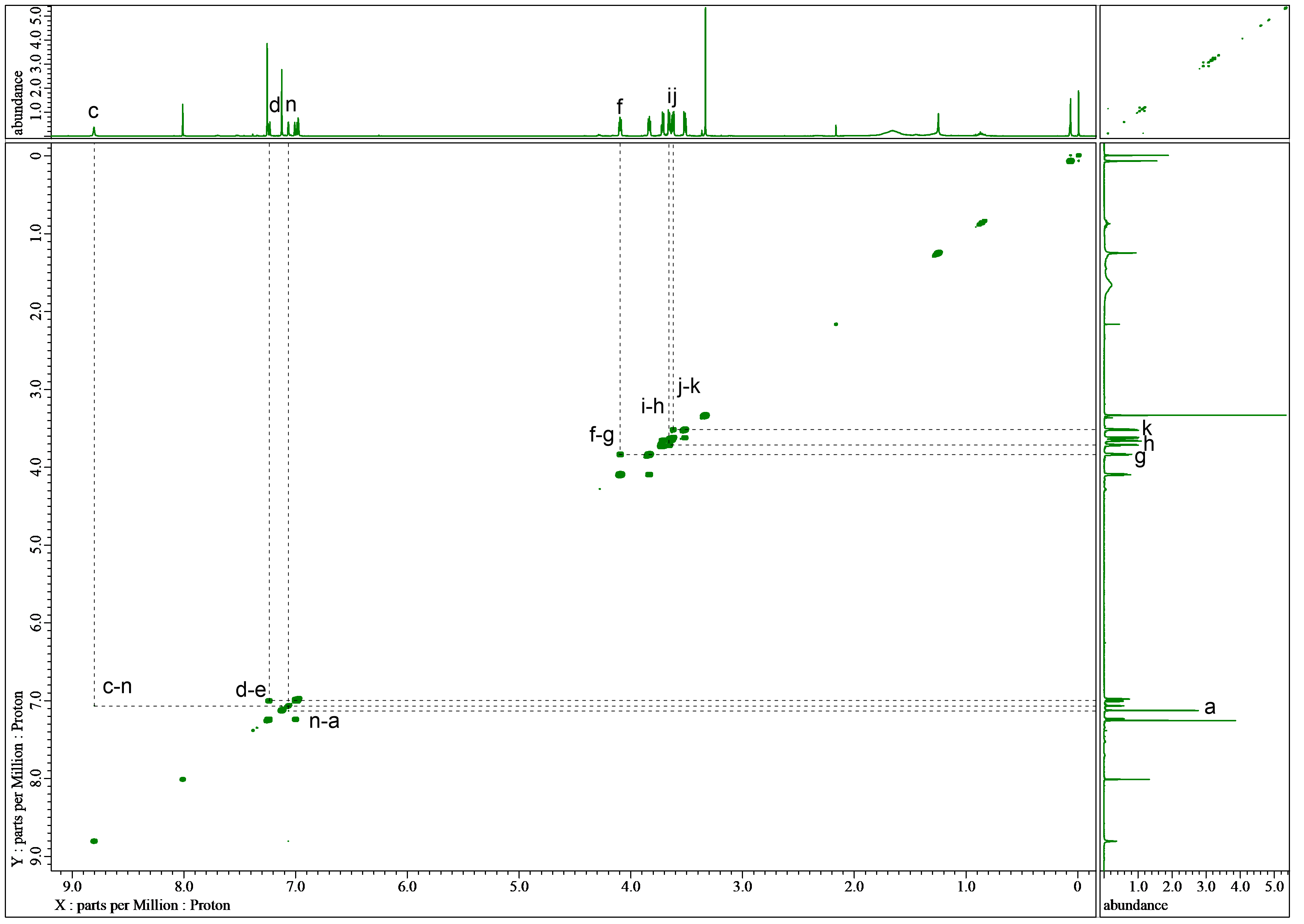


**Figure S7.** ^1^H-^1^H COSY spectrum of **SC** in CDCl_3_ at room temperature.


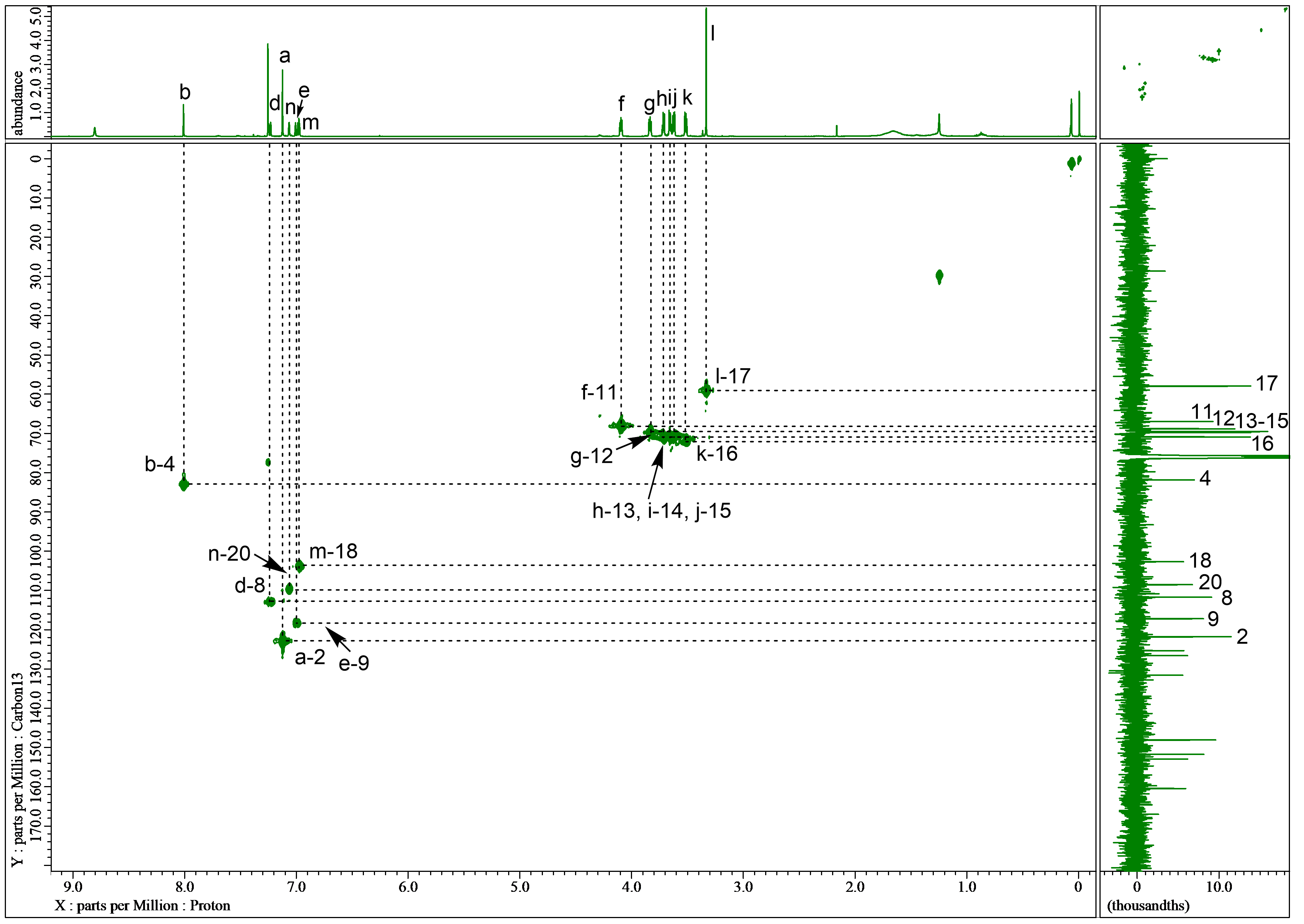


**Figure S8.** HMQC spectrum of **SC** in CDCl_3_ at room temperature.


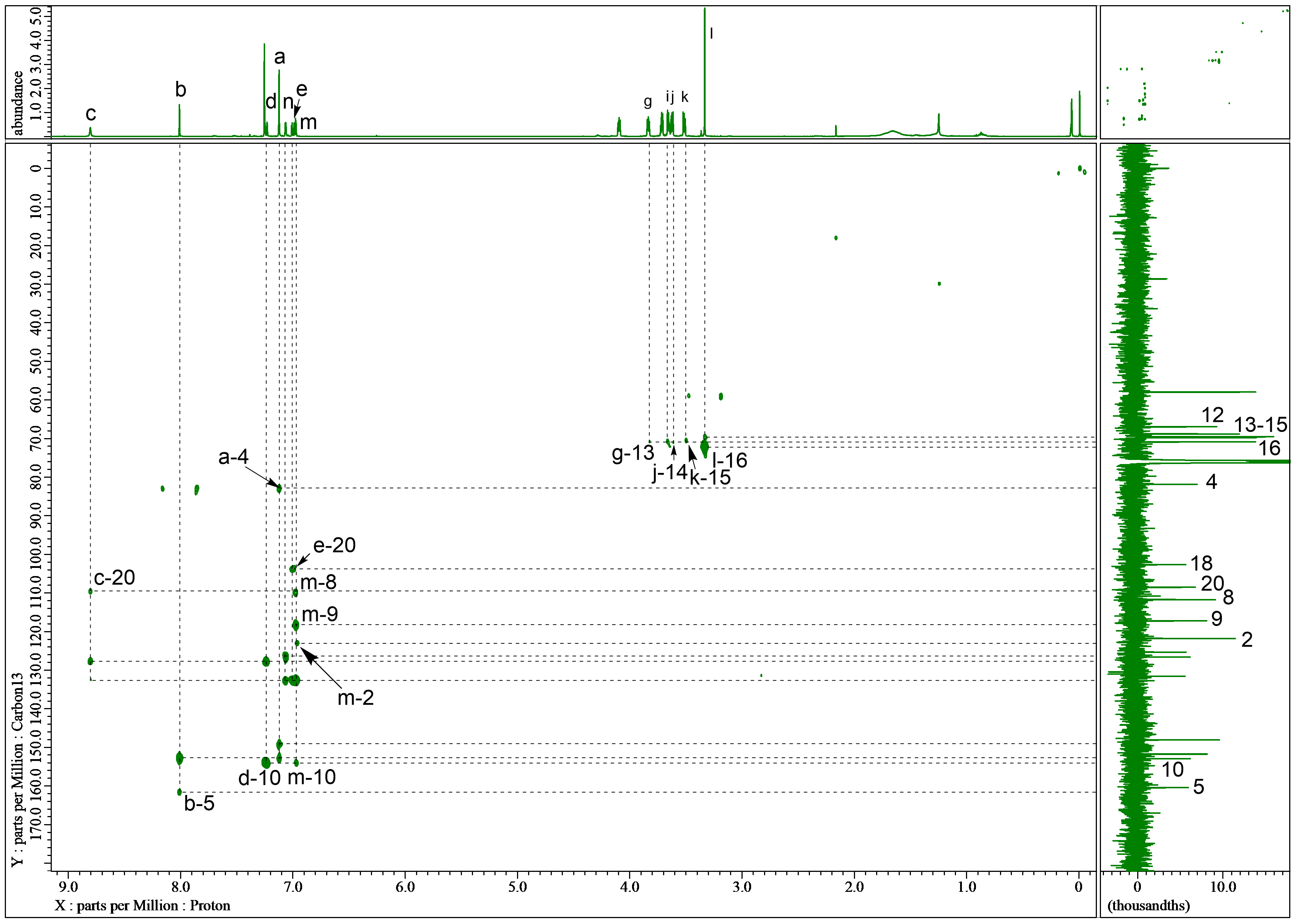


**Figure S9.** HMBC spectrum of **SC** in CDCl_3_ at room temperature.


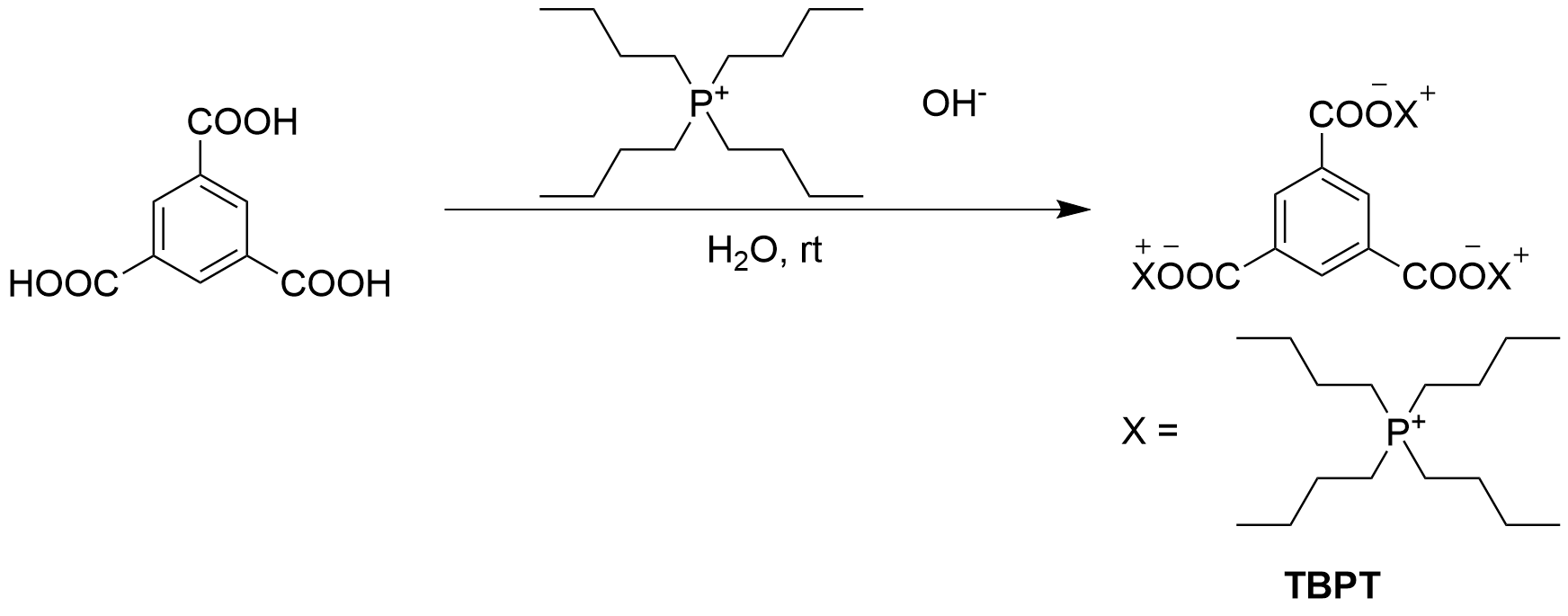


**Tetrabutylphosphonium trimesate** (**TBPT**)**.** Trimesic acid (201 mg, 955 μmol) was dissolved in tetrabutylphosphonium hydroxide in H_2_O (40 wt%, 1.9 mL, 2.90 mmol) and then stirred for 20.5 h at room temperature. The mixture was freeze-dried to obtain **TBPT** as a white solid (862 mg, 875 μmol) in 92% yield. HR-MS (ESI, TOF): *m*/*z* 259.2544 ([TBP]^+^, C_16_H_36_P, calcd. 259.2549), 209.0086 ([C_6_H_3_(COO)_3_2H]^-^, C_9_H_5_O_6_, calcd. 209.0092); ^1^H NMR (DMSO-*d*_6_, 400 MHz, rt) δ_H_ 8.11 (s, 3H), 2.22-2.14 (m, 24H), 1.47-1.37 (m, 48H), 0.93-0.86 (m, 36H); ^13^C NMR (DMSO-*d*_6_, 100 MHz, rt) δ_C_ 170.2, 139.1, 130.2, 23.5, 23.4, 23.3, 22.7, 22.7, 17.6, 17.1, 13.3; m.p. was not obtained due to the highly deliquescency.


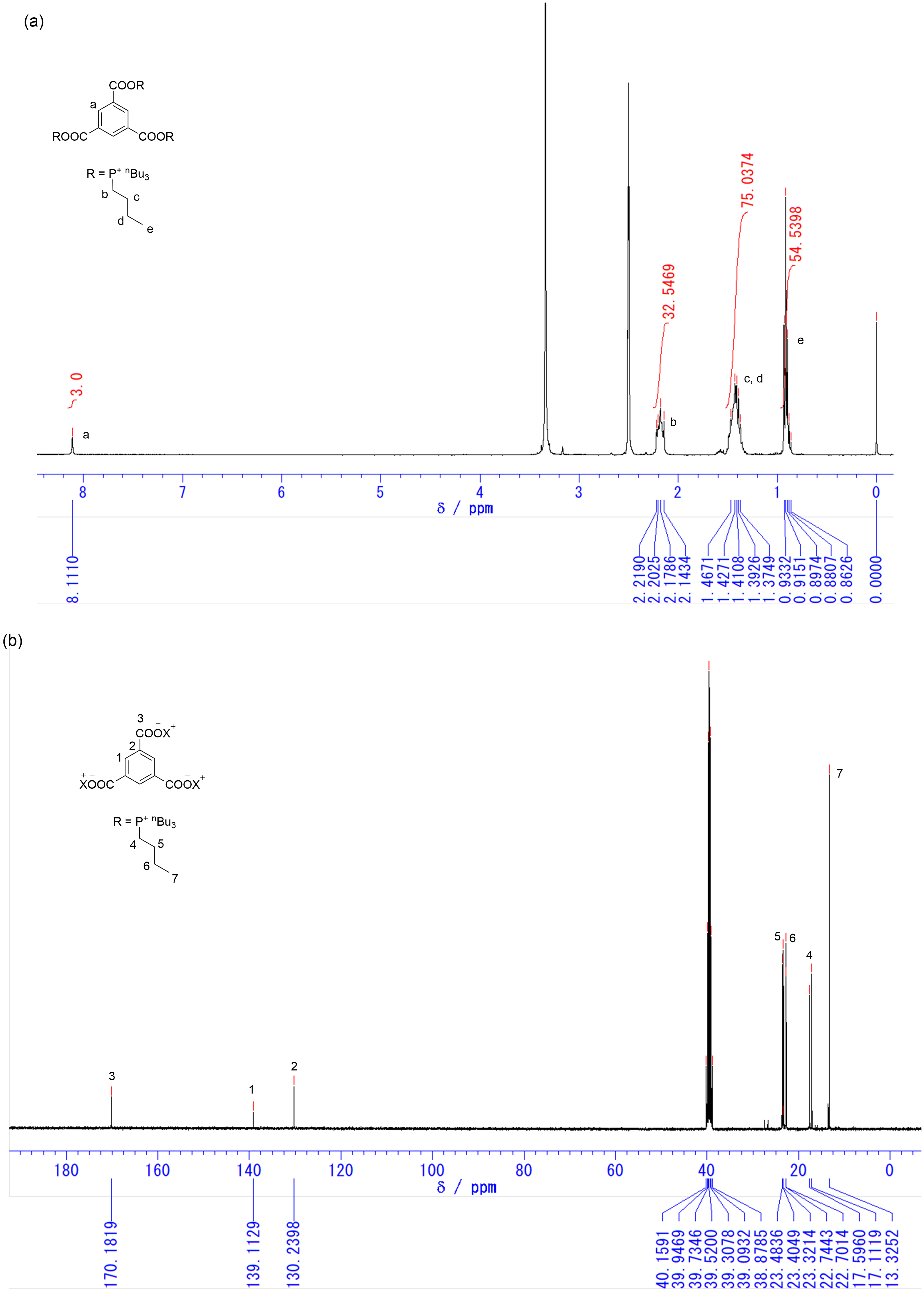


**Figure S10.** (a) ^1^H and (b) ^13^C NMR spectra of **TBPT** in DMSO-*d*_6_ at room temperature.


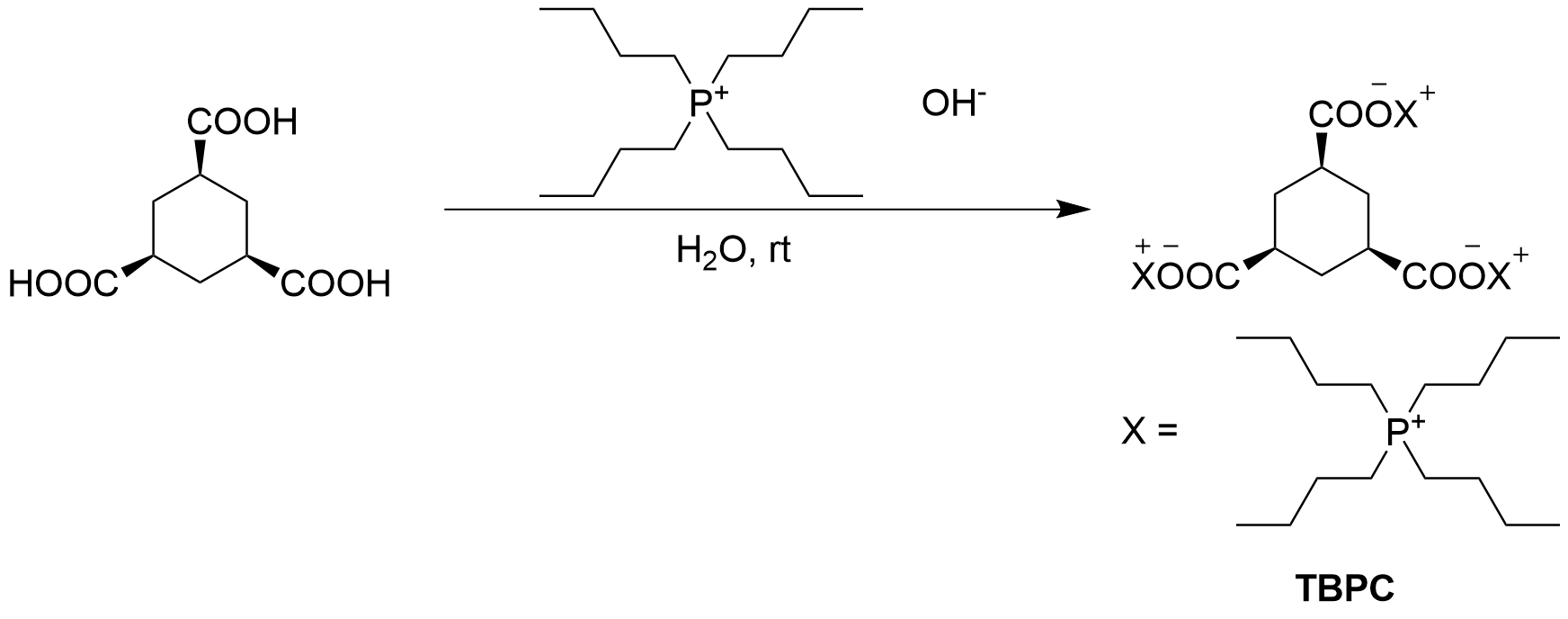


**Tetrabutylphosphonium *cis*,*cis*-cyclohexane-1,3,5-tricarboxylate (TBPC).** *cis*,*cis*-Cyclohexane-1,3,5-tricarboxylic acid (201 mg, 932 μmol) was dissolved in tetrabutylphosphonium hydroxide in H_2_O (40 wt%, 1.9 mL, 2.90 mmol) and stirred for 24 h at room temperature. The mixture was freeze-dried to obtain **TBPC** as colorless oil (1.00 g, 1.01 mmol) in a quantitative yield. HR-MS (ESI, TOF): *m*/*z* 259.2552 ([TBP]^+^, C_16_H_36_P, calcd. 259.2549), 215.0560 ([C_6_H_9_(COO)_3_2H]^-^, C_9_H_11_O_6_, calcd. 215.0560); ^1^H NMR (CDCl_3_, 400 MHz, rt) δ_H_ 2.41 (quint, *J* = 7.5 Hz, 24H), 2.13-2.10 (m, 6H), 1.71-1.64 (m, 3H), 0.95 (t, *J* = 6.1 Hz, 36H); ^13^C NMR (CDCl_3_, 100 MHz, rt) δc 182.0, 47.5, 35.0, 24.1, 23.9, 18.9, 18.4, 13.6.


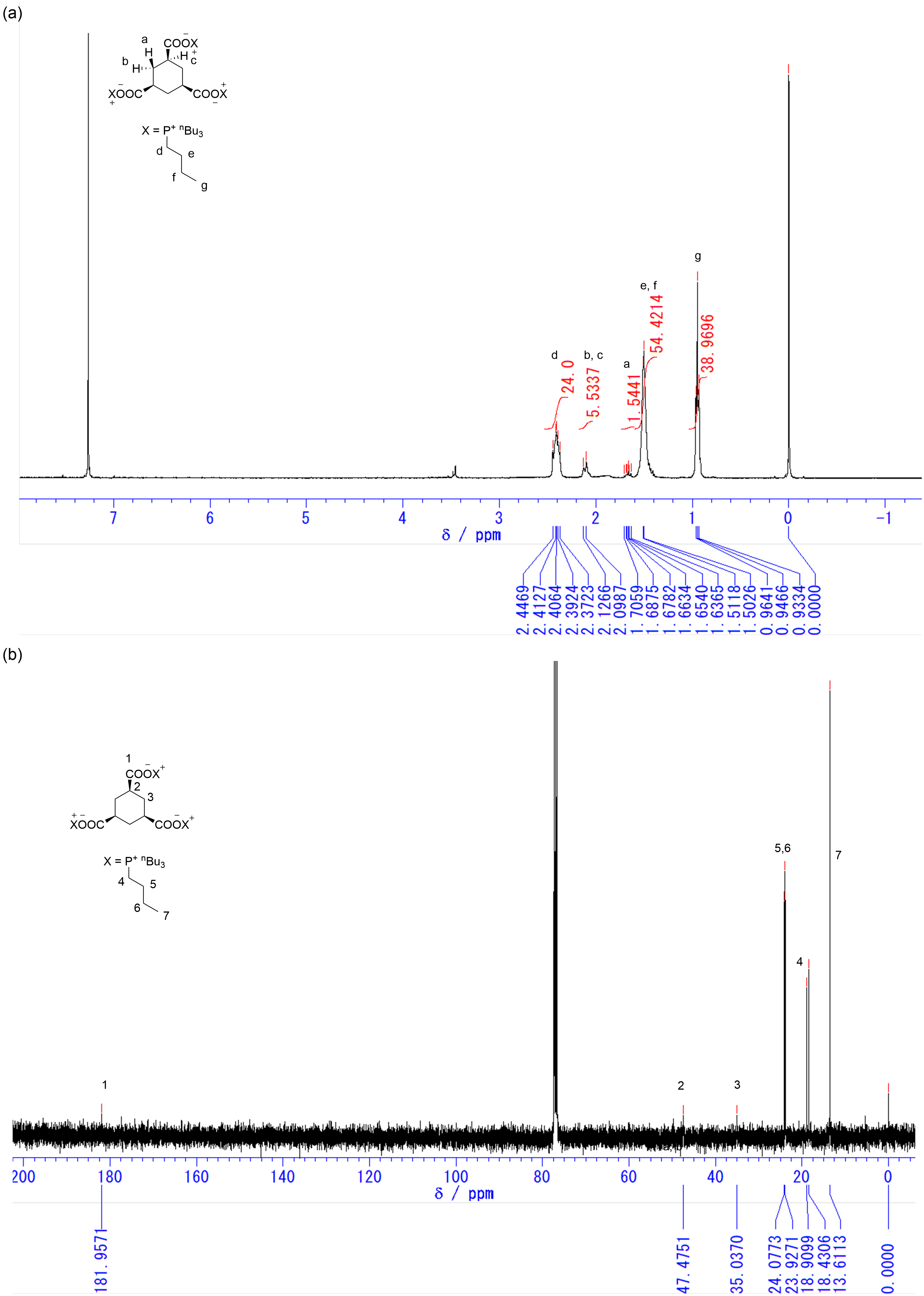


**Figure S11.** (a) ^1^H and (b) ^13^C NMR spectra of **TBPC** in CDCl_3_ at room temperature.

**Photophysical Properties of SC**


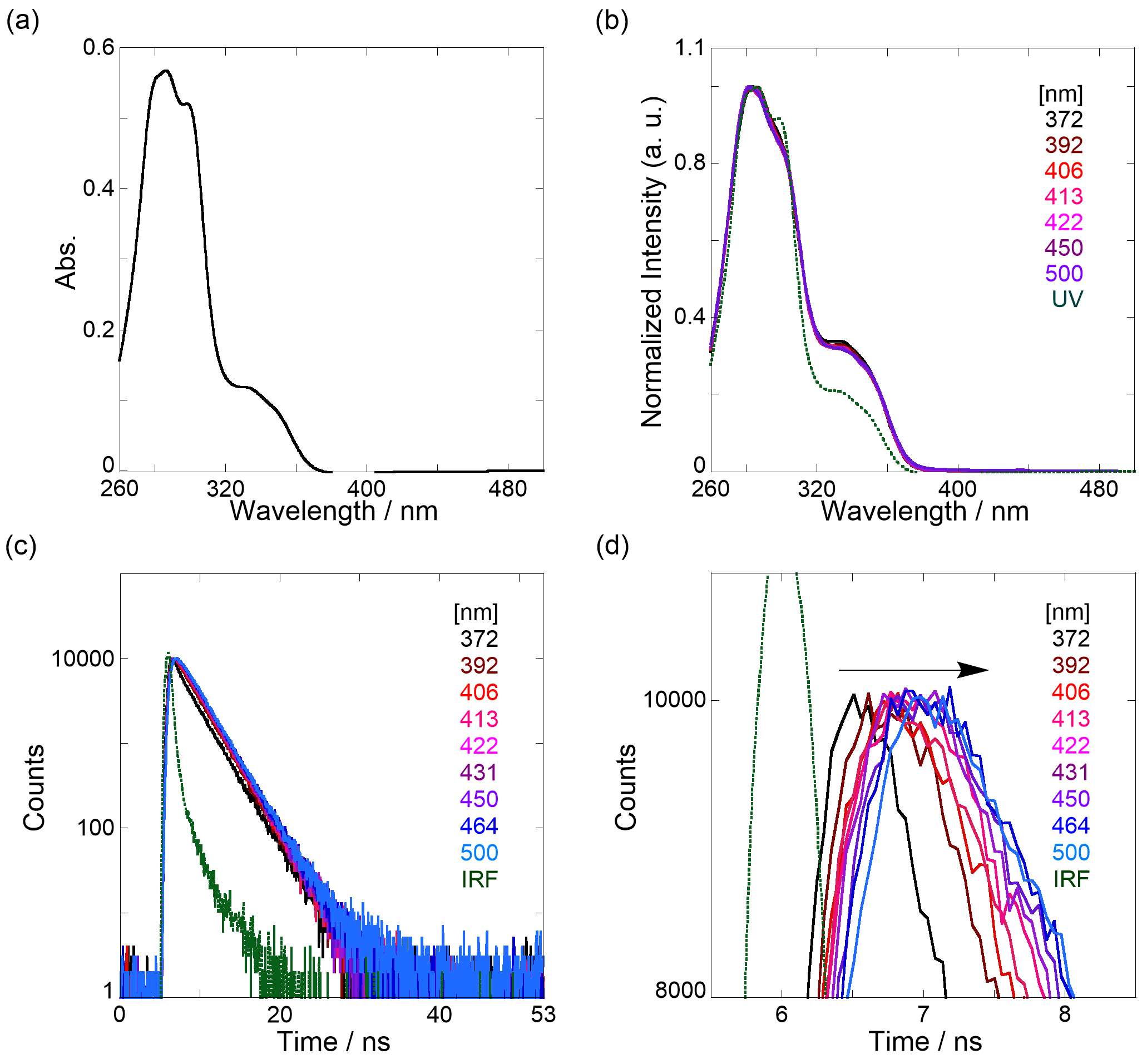


**Figure S12.** (a) UV/vis absorption and (b) excitation spectra of **SC** (8.3 μM) monitored at 372, 392, 406, 413, 422, 450, and 500 nm (from black to violet) in CH_2_Cl_2_, measured in a 1 cm cell; the green dotted line in (b) represents a normalized UV spectrum of (a). (c) Fluorescence lifetime decays (λ_ex_: 340 nm) of **SC** monitored at 372, 392, 406, 413, 422, 431, 450, 464, and 500 nm (from black to sky blue) in CH_2_Cl_2_ at room temperature, measured in a 1 cm cell. (d) Enlarged profiles of (c); the green dotted lines in (c,d) show instrument response function (IRF).

| **Table S1.** Fluorescence lifetimes of **SC** (8.3 μM) in CH_2_Cl_2_*^a^* | | | | | | | | |
| --- | --- | --- | --- | --- | --- | --- | --- | --- |
| λ_em_ / nm | *n*^b^ | *τ*_1_ / ns | *A*_1_ | *τ*_2_ / ns | *A*_2_ | *τ*_3_ / ns | *A*_3_ | χ^2^ |
| 372 | 2 | 0.4 | 0.15 | 2.6 | 0.85 |  |  | 1.0 |
| 392 | 2 | 0.4 | 0.03 | 2.6 | 0.97 |  |  | 1.0 |
| 406 | 1 |  |  | 2.6 |  |  |  | 1.0 |
| 413 | 2 |  |  | 2.6 | 1.02 | 0.4 | -0.02 | 0.9 |
| 422 | 2 |  |  | 2.6 | 1.03 | 0.4 | -0.03 | 1.0 |
| 431 | 2 |  |  | 2.6 | 1.05 | 0.4 | -0.05 | 1.2 |
| 450 | 2 |  |  | 2.6 | 1.07 | 0.3 | -0.07 | 1.0 |
| 464 | 2 |  |  | 2.6 | 1.08 | 0.3 | -0.08 | 1.0 |
| 500 | 2 |  |  | 2.6 | 1.09 | 0.3 | -0.09 | 1.0 |
| *^a^*Fluorescence lifetime (*τ*_i_) and relative abundance (*A*_i_) of each component. *^b^*Number of components. | | | | | | | | |

**Photophysical Properties of SC with Triethylamine**


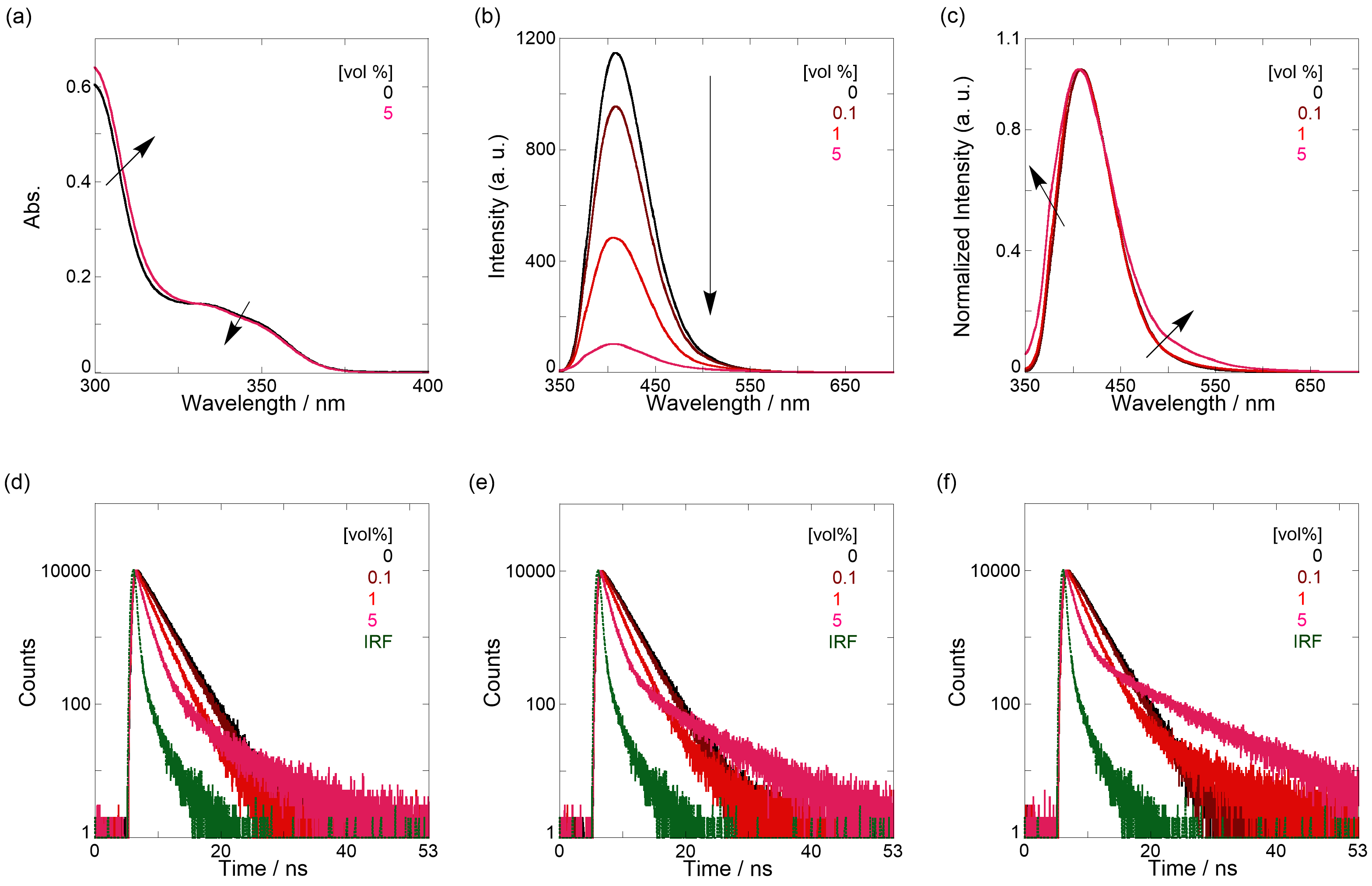


**Figure S13.** (a) UV/vis absorption, (b) fluorescence, (c) normalized fluorescence spectra (λ_ex_: 340 nm), and (d–f) fluorescence lifetime decays (λ_ex_: 340 nm) of **SC** (8.9 μM, black) monitored at (d) 392, (e) 406, and (f) 431 nm in CH_2_Cl_2_ following the gradual addition of triethylamine (0.1-5 vol%, brown to magenta) at (a–c) 25 °C and (d–f) room temperature, measured in a 1 cm cell, where the green dotted lines in (d–f) represent IRF.

| **Table S2.** Fluorescence lifetimes of **SC** (8.9 μM) in CH_2_Cl_2_ following the gradual addition of triethylamine (TEA) (0.1–5 vol%)*^a^* | | | | | | | | | | | | |
| --- | --- | --- | --- | --- | --- | --- | --- | --- | --- | --- | --- | --- |
| λ_em_/nm | TEA/vol% | *τ*_1_/ns | *τ*_2_/ns | *τ*_3_/ns | *τ*_4_/ns | *τ*_5_/ns | *A*_1_ | *A*_2_ | *A*_3_ | *A*_4_ | *A*_5_ | χ^2^ |
| 392 | 0 | 0.3 | 2.6 |  |  |  | 0.04 | 0.96 |  |  |  | 1.0 |
| 406 |  |  | 2.6 |  |  |  |  |  |  |  |  | 1.3 |
| 431 |  |  | 2.6 | 0.5 |  |  |  | 1.04 | -0.04 |  |  | 1.3 |
| 392 | 0.1 | 0.3 | 2.5 |  |  |  | 0.05 | 0.95 |  |  |  | 1.7 |
| 406 |  | 0.2 | 2.5 |  |  |  | 0.01 | 0.99 |  |  |  | 2.0 |
| 431 |  |  | 2.5 | 0.4 |  |  |  | 1.02 | -0.02 |  |  | 1.7 |
| 392 | 1 | 0.1 | 2.1 |  | 1.3 |  | 0.07 | 0.24 |  | 0.69 |  | 1.9 |
| 406 |  | 0.1 | 3.7 |  | 1.8 |  | 0.04 | 0.88 |  | 0.08 |  | 1.8 |
| 431 |  |  |  |  | 1.8 | 6.6 |  |  |  | 0.95 | 0.05 | 1.7 |
| 392 | 5 | 0.3 |  |  | 1.2 | 6.1 | 0.23 |  |  | 0.69 | 0.08 | 1.5 |
| 406 |  | 0.2 |  |  | 1.1 | 7.2 | 0.17 |  |  | 0.69 | 0.14 | 1.4 |
| 431 |  | 0.3 |  |  | 1.0 | 8.8 | 0.15 |  |  | 0.59 | 0.26 | 1.5 |
| *^a^*Fluorescence lifetime (*τ*_i_) and relative abundance (*A*_i_) of each component, analyzed by deconvolution fitting. | | | | | | | | | | | | |


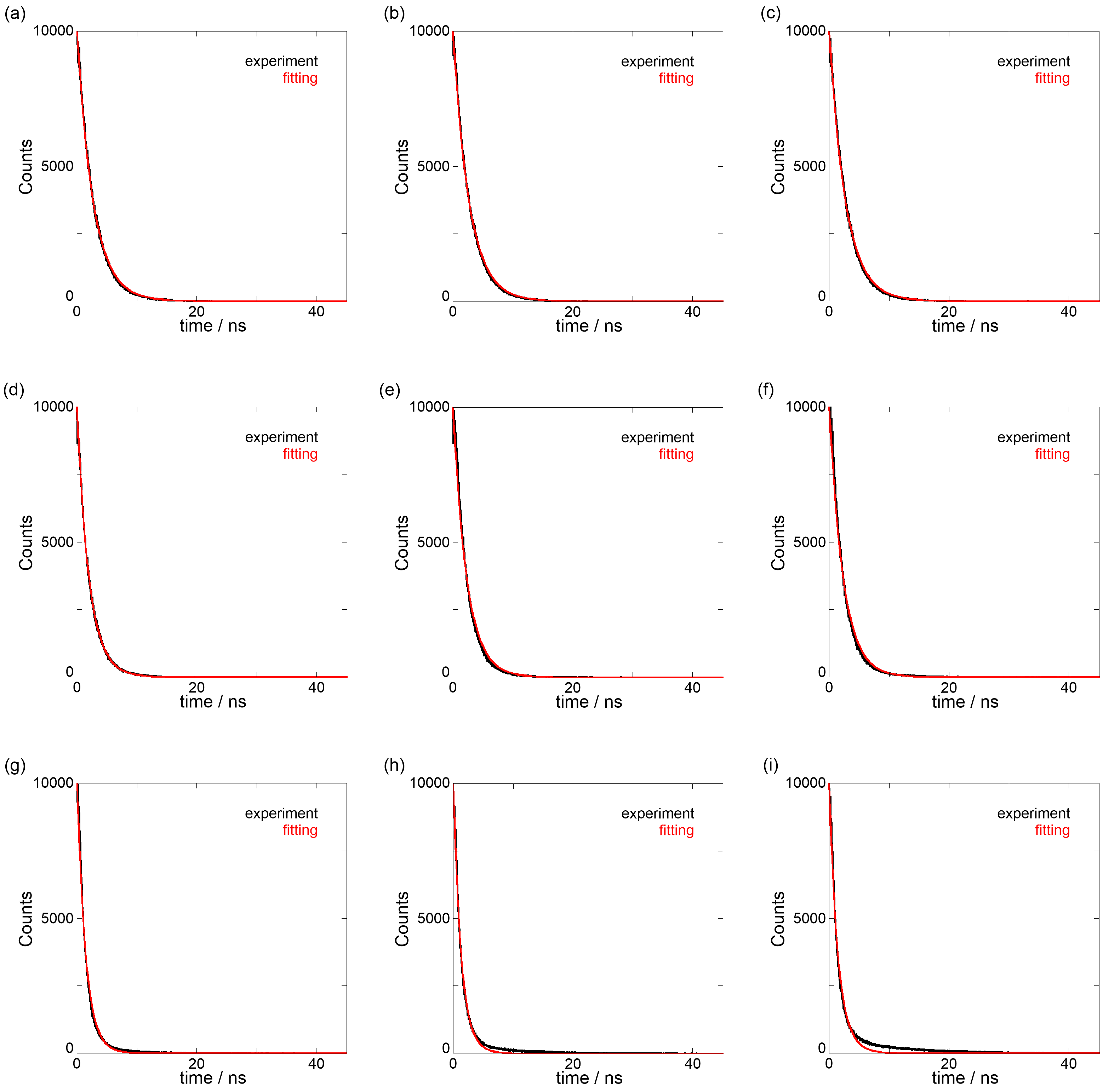


**Figure S14.** Fluorescence lifetime decays of **SC** (8.9 μM) in CH_2_Cl_2_ with triethylamine (a–c: 0.1, d–f: 1, and g–i: 5 vol%) at room temperature monitored at (a,d,g) 392, (b,e,h) 406, and (c,f,i) 431 nm, measured in a 1 cm cell, where the black lines represent experimental decay, and the red lines are the corresponding fitted curves.

| **Table S3.** Fluorescence lifetimes of **SC** (8.9 μM) in CH_2_Cl_2_ following the gradual addition of triethylamine (TEA) (0.1–5 vol%)*^a^* | | | | |
| --- | --- | --- | --- | --- |
| λ_em_/nm | TEA/vol% | *β* | *τ*/ns | *r* |
| 392 | 0.1 | 1 | 2.7 | 0.999 |
| 406 |  | 1 | 2.7 | 0.999 |
| 431 |  | 1 | 2.7 | 0.999 |
| 392 | 1 | 0.98 | 2.0 | 0.999 |
| 406 |  | 1 | 2.3 | 0.997 |
| 431 |  | 1 | 2.3 | 0.997 |
| 392 | 5 | 1 | 1.4 | 0.996 |
| 406 |  | 0.99 | 1.3 | 0.998 |
| 431 |  | 0.96 | 1.4 | 0.994 |
| *^a^*Average fluorescence lifetime (*τ*) and dispersion factor (*β*), analyzed by Kohlrausch decay model. | | | | |

**Discussion**

The addition of triethylamine (TEA) to the CH_2_Cl_2_ solution of **SC** causes an anionic indole side chain that emits as mono, di, and tri-anion excited species. Due to the multicomponent mixture of excited species, the decay profiles were difficult to analyze (χ^2^ > 1.3) by the common deconvolution fitting (see Table S2). Therefore, the decays were fitted by the stretched exponential (Kohlrausch decay^[[5]](#footnote-6)^) function to afford an average fluorescence lifetime (*τ*) and dispersion factor (*β*), as written in equation S1:

$$I\left( t \right)=I_{0}\exp\left\{ {-\left( \frac{t}{\tau} \right)}^{\beta} \right\} (S1)$$

where *I*(t) and *I*_0_ represent the photon counts at arbitrary and initial times, *t* is time, *τ* is average lifetime, and *β* is dispersion factor, respectively. As shown in Figure S14, the decays were well fitted by this model to give the shortening *τ* and much dispersed *β* upon the gradual addition of TEA listed in Table S3, indicating the formation of gradual-anionic **SC** fluorescent species.

**Photophysical Properties of ref**


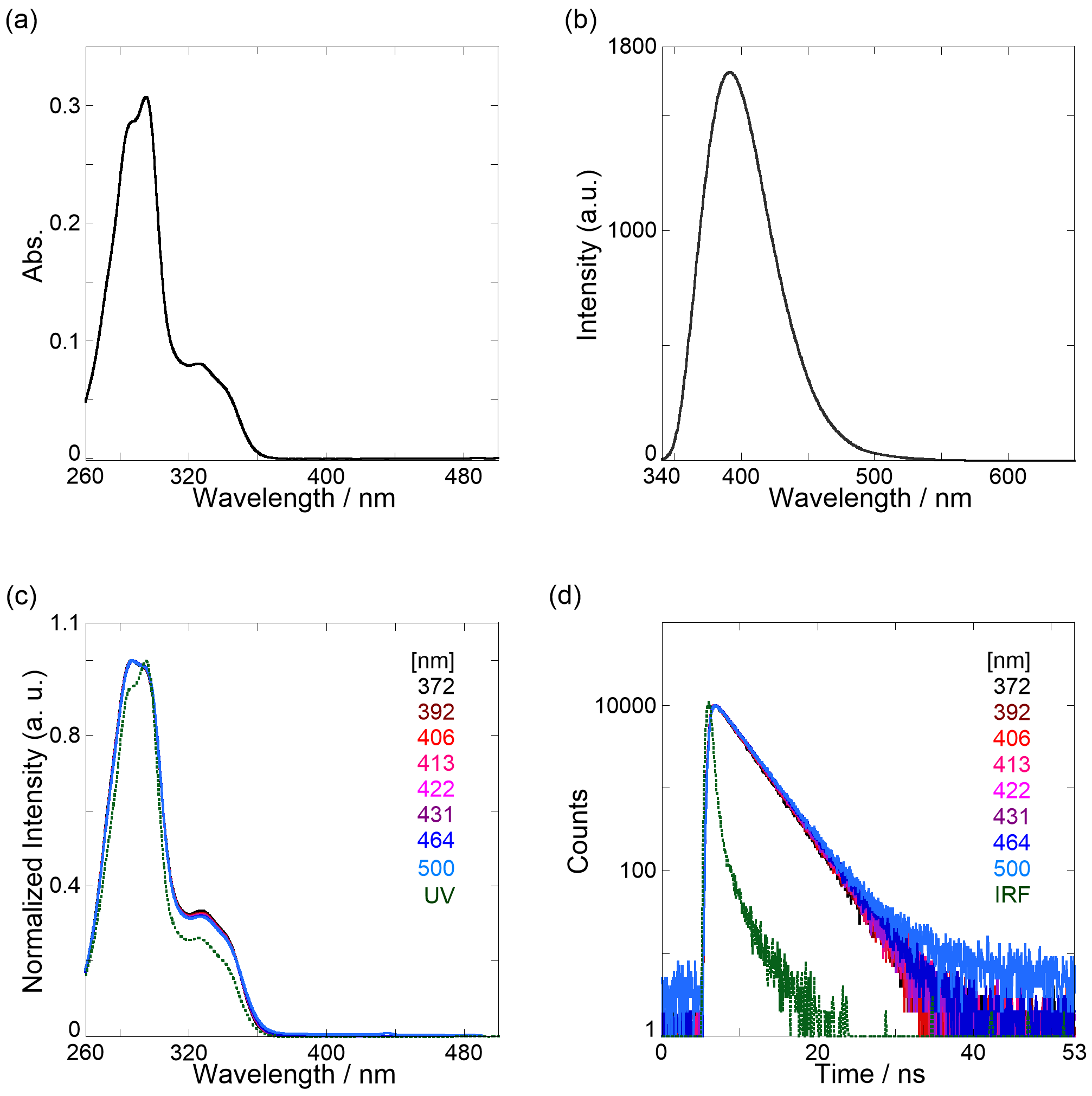


**Figure S15.** (a) UV/vis absorption and (b) fluorescence spectra (λ_ex_: 330 nm) of **ref** (15.6 μM) in CH_2_Cl_2_, measured in a 1cm cell. (c) Excitation spectra and (d) fluorescence lifetime decays (λ_ex_: 340 nm) of **ref** (15.6 μM) monitored at 372, 392, 406, 413, 422, 431, 464, and 500 nm (from black to sky blue) in CH_2_Cl_2_; measurements were performed in a 1 cm cell. The green dotted lines in (c) and (d) represent a normalized UV spectrum of (a) and IRF, respectively.

| **Table S4.** Fluorescence lifetimes of **ref** (15.6 μM) in CH_2_Cl_2_*^a^* | | | | | | | | |
| --- | --- | --- | --- | --- | --- | --- | --- | --- |
| λ_em_ / nm | *n*^b^ | *τ*_1_ / ns | *A*_1_ | *τ*_2_ / ns | *A*_2_ | *τ*_3_ / ns | *A*_3_ | χ^2^ |
| 372 | 2 | 1.4 | 0.04 | 3.2 | 0.96 |  |  | 1.1 |
| 392 | 2 | 0.8 | 0.02 | 3.2 | 0.98 |  |  | 1.1 |
| 406 | 1 |  |  | 3.2 |  |  |  | 1.2 |
| 413 | 1 |  |  | 3.2 |  |  |  | 1.1 |
| 422 | 1 |  |  | 3.2 |  |  |  | 1.2 |
| 431 | 1 |  |  | 3.3 |  |  |  | 1.1 |
| 464 | 1 |  |  | 3.3 |  |  |  | 1.4 |
| 500 | 2 |  |  | 3.4 | 0.99 | 0.1 | 0.01 | 1.2 |
| *^a^*Fluorescence lifetime (*τ*_i_) and relative abundance (*A*_i_) of each component. *^b^*Number of components. | | | | | | | | |

**Sensing Data of ref**


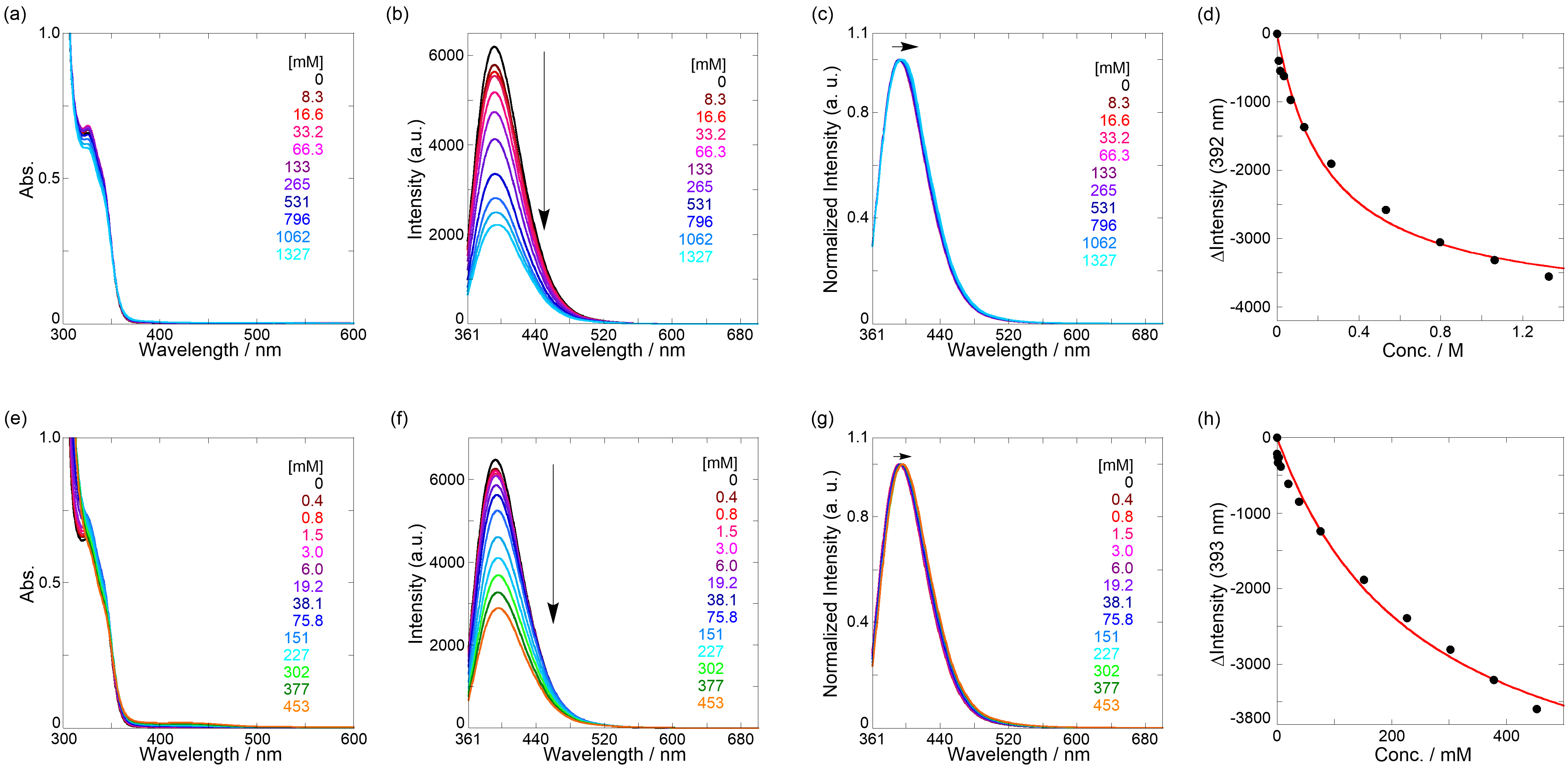


**Figure S16.** (a,e) UV/vis absorption and (b,f) fluorescence (λ_ex_: 351 nm) spectra of **ref** (a,b: 1.36 mM, e,f: 1.35 mM; black) following the addition of (a,b) **MB** (8.3–1327 mM; from brown to light blue) or (e,f) **TBPB** (0.4–453 mM; from brown to orange) in CH_2_Cl_2_ at 25 °C; measurements were conducted in a 1 mm cell. The excitation wavelength at which comparable absorbances were obtained was selected. (c,g) Normalized fluorescence spectra of (b) or (g). (d,h) Non-linear least-squares fittings, assuming the 1:1 stoichiometry with **ref** and (d) **MB** or (h) **TBPB** monitored at 392 and 393 nm, to determine the binding constants at 25 °C.

**Sensing Data of SC**


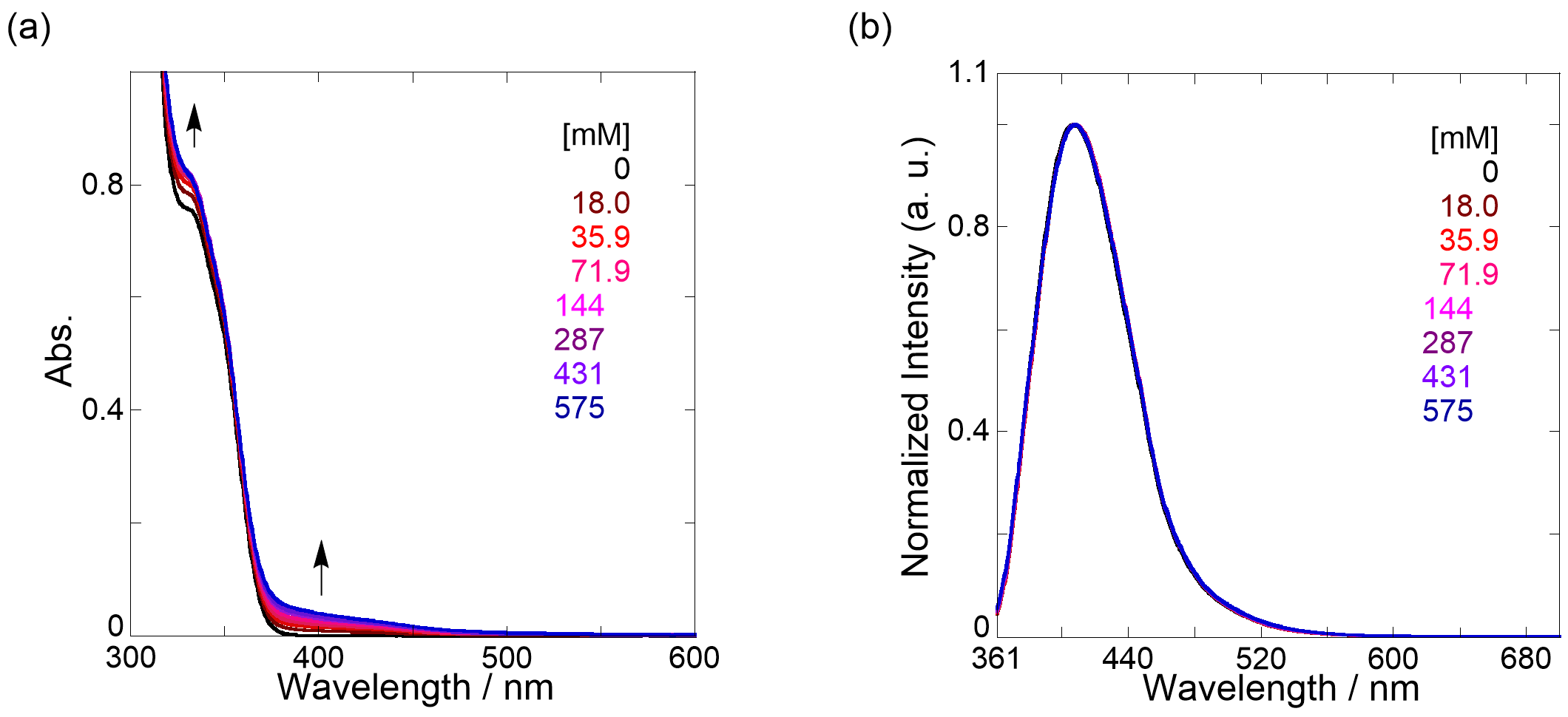


**Figure S17.** (a) UV/vis absorption and (b) normalized fluorescence spectra of (λ_ex_: 351 nm) of **SC** (421 μM; black) following the gradual addition of **MB** (18.0–575 mM; from brown to blue) in CH_2_Cl_2_ at 25 °C; measurements were conducted in a 1 mm cell.


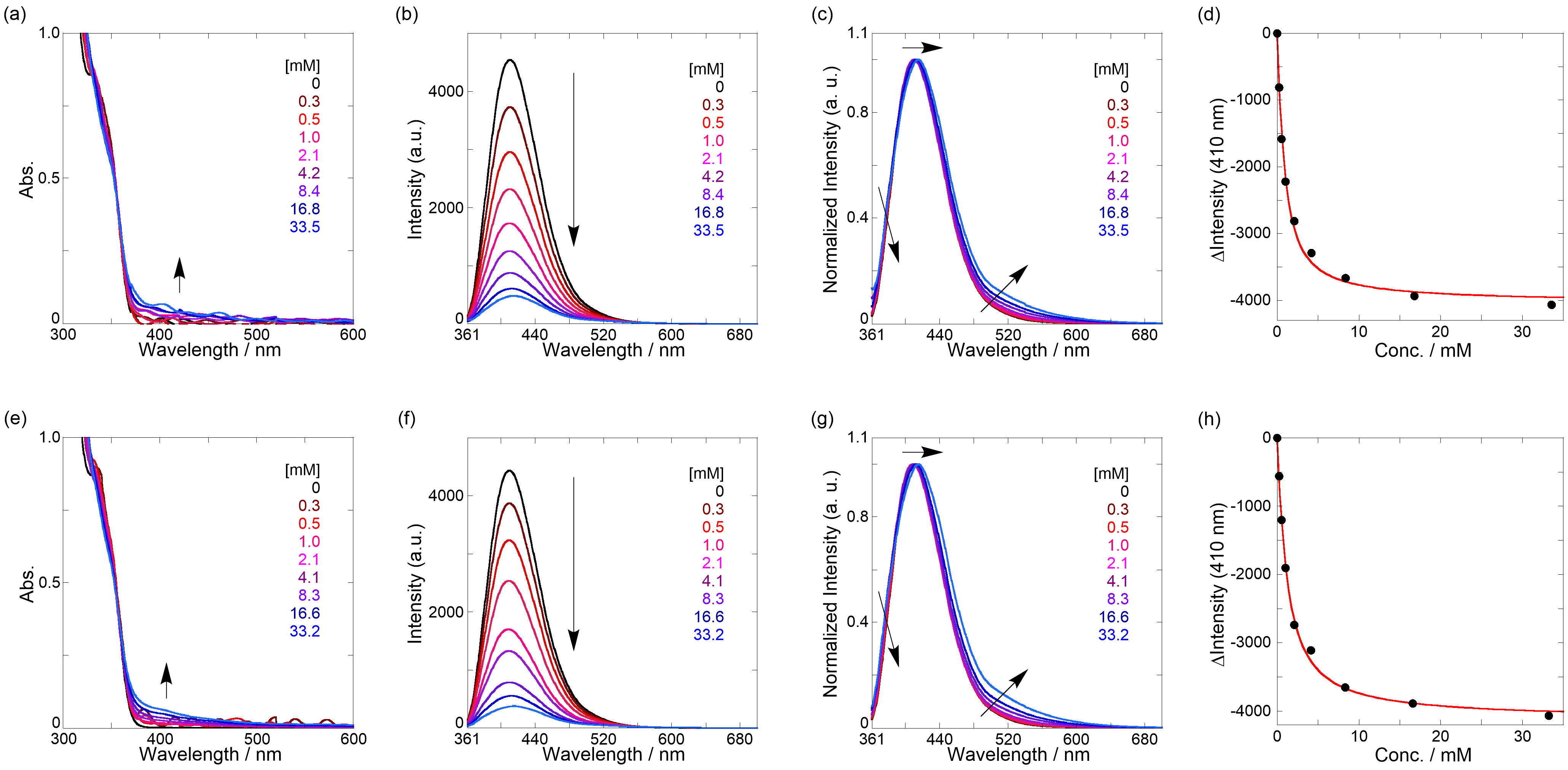


**Figure S18.** (a,e) UV/vis absorption and (b,f) fluorescence (λ_ex_: 351 nm) spectra of **SC** (a,b: 480 μM, e,f: 493 μM; black) following the addition of **TBPB** (a,b: 0.3–33.5 mM, e,f: 0.3–33.2 mM; from brown to blue) in CH_2_Cl_2_ at (a,b) 5 and (e,f) 15 °C; measurements were conducted in a 1 mm cell. The excitation wavelength at which comparable absorbances were obtained was selected. (c,g) Normalized fluorescence spectra of (b) or (f). (d,h) Non-linear least-squares fittings, assuming the 1:1 stoichiometry with **SC** and **TBPB** monitored at 410 nm, to determine the binding constants at (d) 5 and (h) 15 °C.


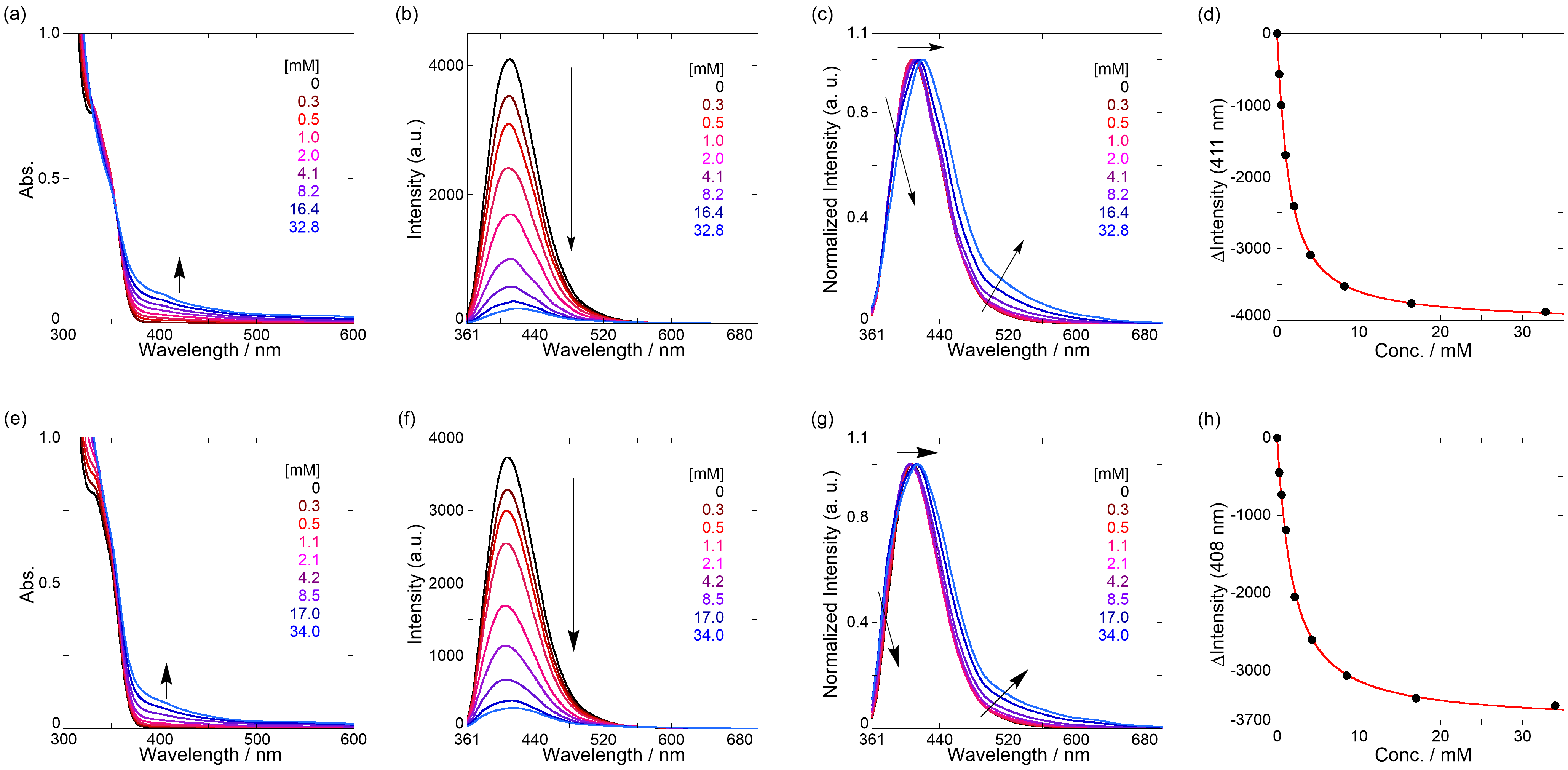


**Figure S19.** (a,e) UV/vis absorption and (b,f) fluorescence (λ_ex_: 351 nm) spectra of **SC** (a,b: 468 μM, e,f: 452 μM; black) following the addition of **TBPB** (a,b: 0.3–32.8 mM, e,f: 0.3–34.0 mM; from brown to blue) in CH_2_Cl_2_ at (a,b) 25 and (e,f) 35 °C; measurements were conducted in a 1 mm cell. The excitation wavelength at which comparable absorbances were obtained was selected. (c,g) Normalized fluorescence spectra of (b) or (f). (d,h) Non-linear least-squares fittings, assuming the 1:1 stoichiometry with **SC** and **TBPB** monitored at 411 and 408 nm, to determine the binding constants at (d) 25 and (h) 35 °C.


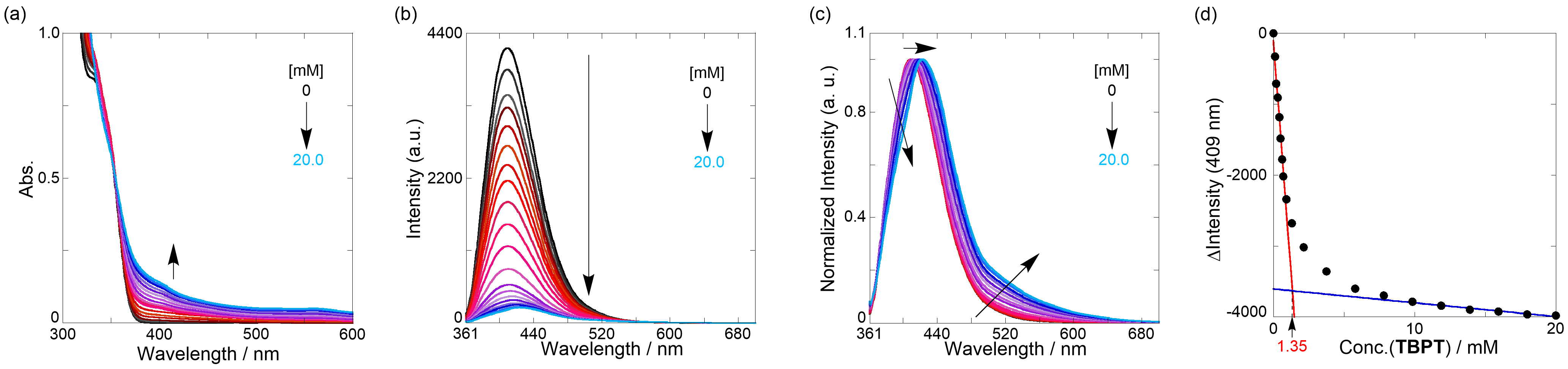


**Figure S20.** (a) UV/vis absorption and (b) fluorescence (λ_ex_: 351 nm) spectra of **SC** (460 μM, black) following the addition of **TBPT** (0.1–20.0 mM; from dark gray to light blue) in CH_2_Cl_2_ at 25 °C; measurements were conducted in a 1 mm cell. The excitation wavelength at which comparable absorbances were obtained was selected. (c) Normalized fluorescence spectra of (b). (d) Fluorescence intensity changes at 409 nm of **SC** (460 μM) as a function of the **TBPT** concentration (red; *r* = 0.994, blue; *r* = 0.985).

**Discussion**

As shown in Figure S20, the fluorescence intensity induced by the addition of **TBPT** into **SC** showed a saturation behavior to reach a quasi-plateau at 10–20 mM, that is, molar ratio plot. The inflection point estimated by extrapolating the fluorescence changes in the low (red) and high concentration (blue) ranges was 1.35 mM, apparently indicating the complexation stoichiometry of 1:3. This result indicates that the three indole recognition sites in **SC** independently recognize **TBPT**, which should be therefore treated the stoichiometry of **SC** ⸦ **TBPT** as 1:1.

**
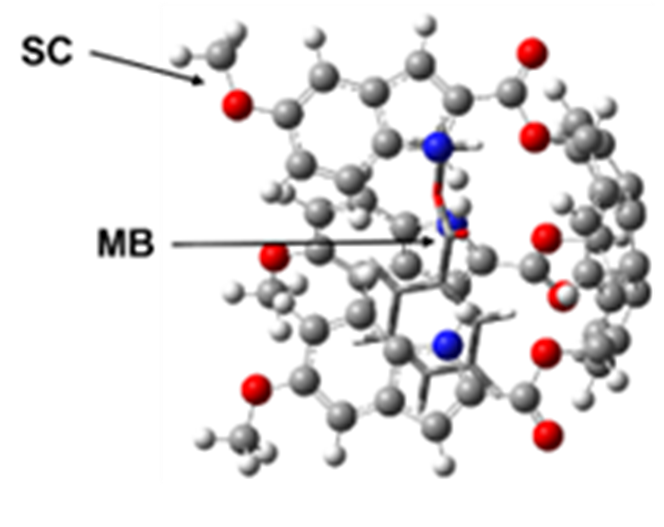
**

**Figure S20-2.** Quantum chemistry calculation-based optimized structure of **SC** ⸦ **MB**.


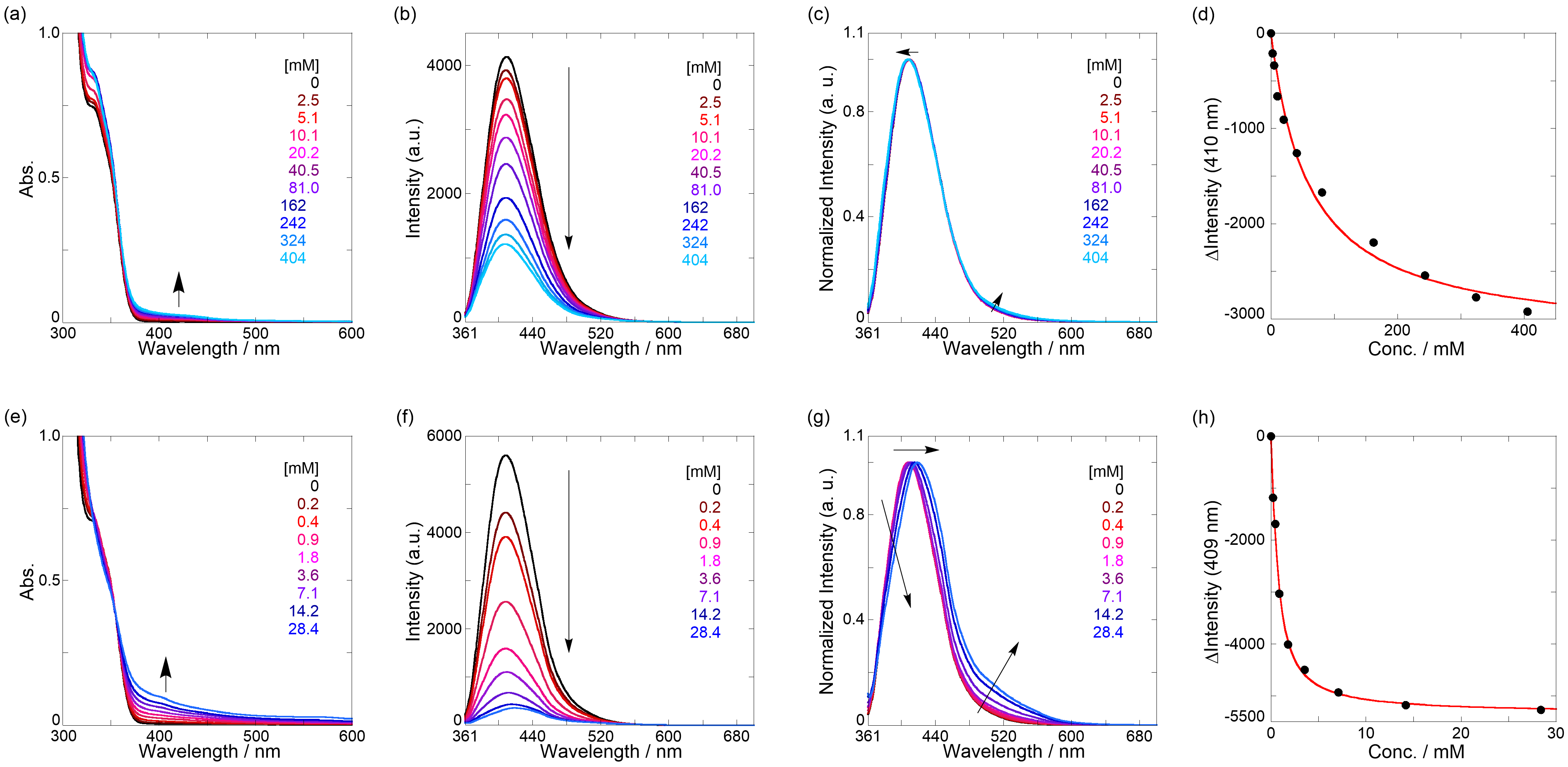


**Figure S21.** (a,e) UV/vis absorption and (b,f) fluorescence (λ_ex_: 351 nm) spectra of **SC** (a,b: 415 μM, e,f: 458 μM; black) following the addition of (a,b) **DI** (2.5–404 mM; from brown to light blue) or (e,f) **TBPI** (0.2–28.4 mM; from brown to blue) in CH_2_Cl_2_ at 25 °C; measurements were conducted in a 1 mm cell. The excitation wavelength at which comparable absorbances were obtained was selected. (c,g) Normalized fluorescence spectra of (b) or (f). (d,h) Non-linear least-squares fittings, assuming the 1:1 stoichiometry with **SC** and (d) **DI** or (h) **TBPI** monitored at (d) 410 or (h) 409 nm, to determine the binding constants at 25 °C.


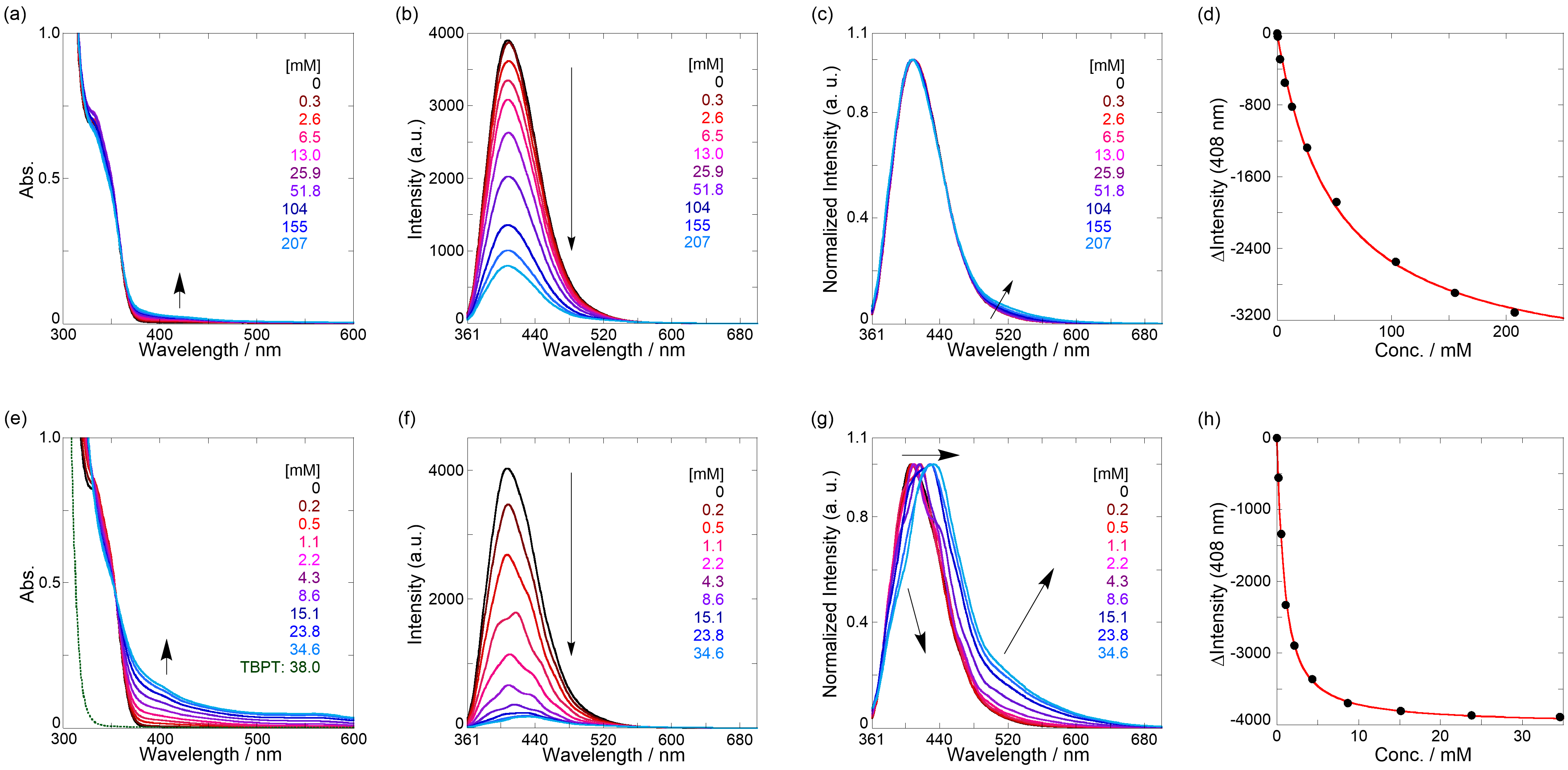


**Figure S22.** (a,e) UV/vis absorption and (b,f) fluorescence (λ_ex_: 351 nm) spectra of **SC** (a,b: 419 μM, e,f: 458 μM; black) following the addition of (a,b) **TT** (0.3–207 mM; from brown to sky blue) or (e,f) **TBPT** (0.2–34.6 mM; from brown to sky blue) in CH_2_Cl_2_ at 25 °C; measurements were conducted in a 1 mm cell. The excitation wavelength at which comparable absorbances were obtained was selected. The green dotted line in (e) represents UV spectra of **TBPT** (38.0 mM) in CH_2_Cl_2_ at 25 °C; measurements were conducted in a 1 mm cell. (c,g) Normalized fluorescence spectra of (b) or (f). (d,h) Non-linear least-squares fittings, assuming the 1:1 stoichiometry with **SC** and (d) **TT** or (h) **TBPT** monitored at 408 nm, to determine the binding constants at 25 °C.


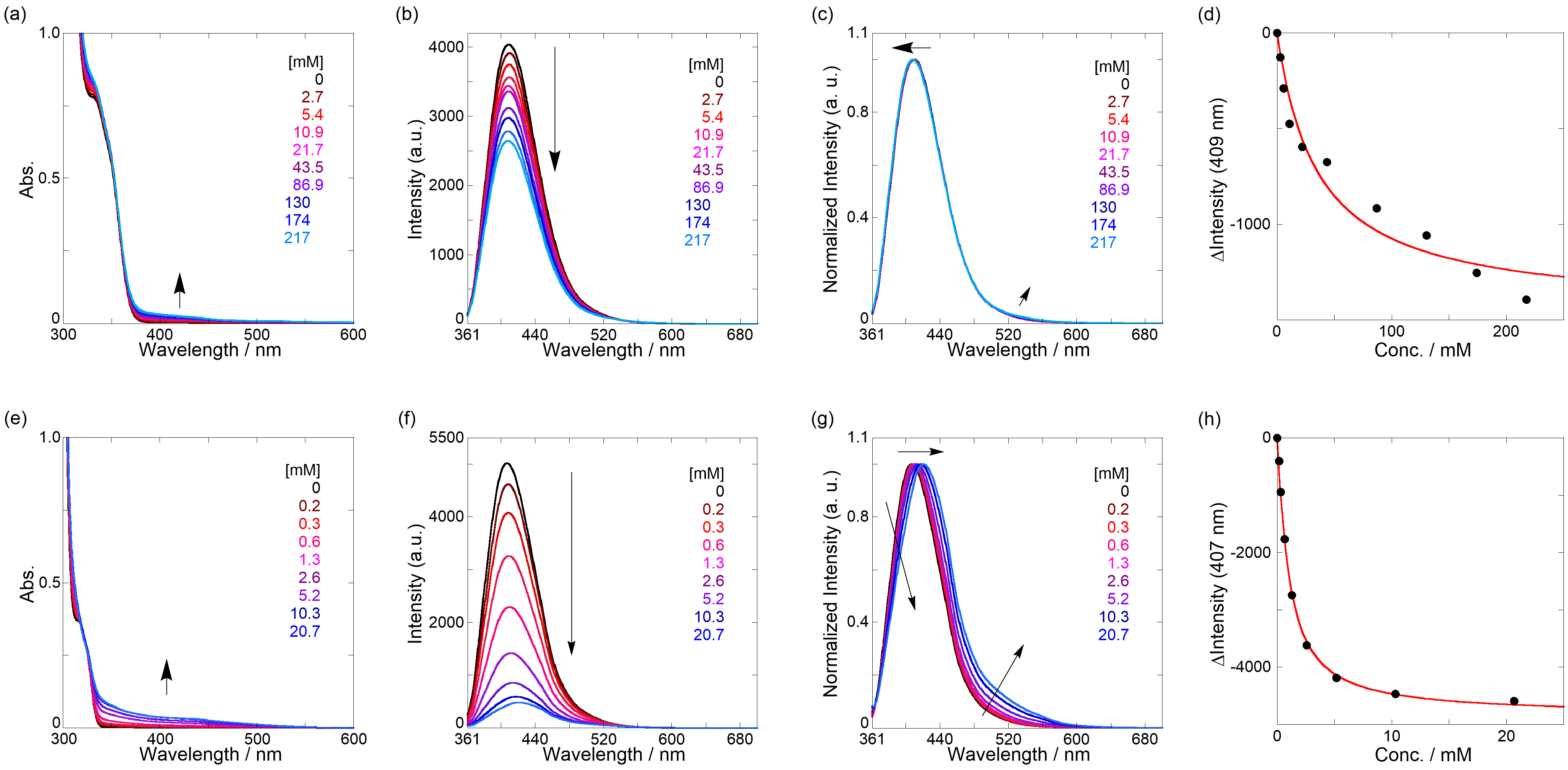


**Figure S23.** (a,e) UV/vis absorption and (b,f) fluorescence (λ_ex_: 351 nm) spectra of **SC** (a,b: 433 μM, e,f: 480 μM; black) following the addition of (a,b) **TC** (2.7–217 mM; from brown to sky blue) or (e,f) **TBPC** (0.2–20.7 mM; from brown to blue) in CH_2_Cl_2_ at 25 °C; measurements were conducted in a 1 mm cell. The excitation wavelength at which comparable absorbances were obtained was selected. (c,g) Normalized fluorescence spectra of (b) or (f). (d,h) Non-linear least-squares fittings, assuming the 1:1 stoichiometry with **SC** and (d) **TC** or (h) **TBPC** monitored at (d) 409 or (h) 407 nm, to determine the binding constants at 25 °C.


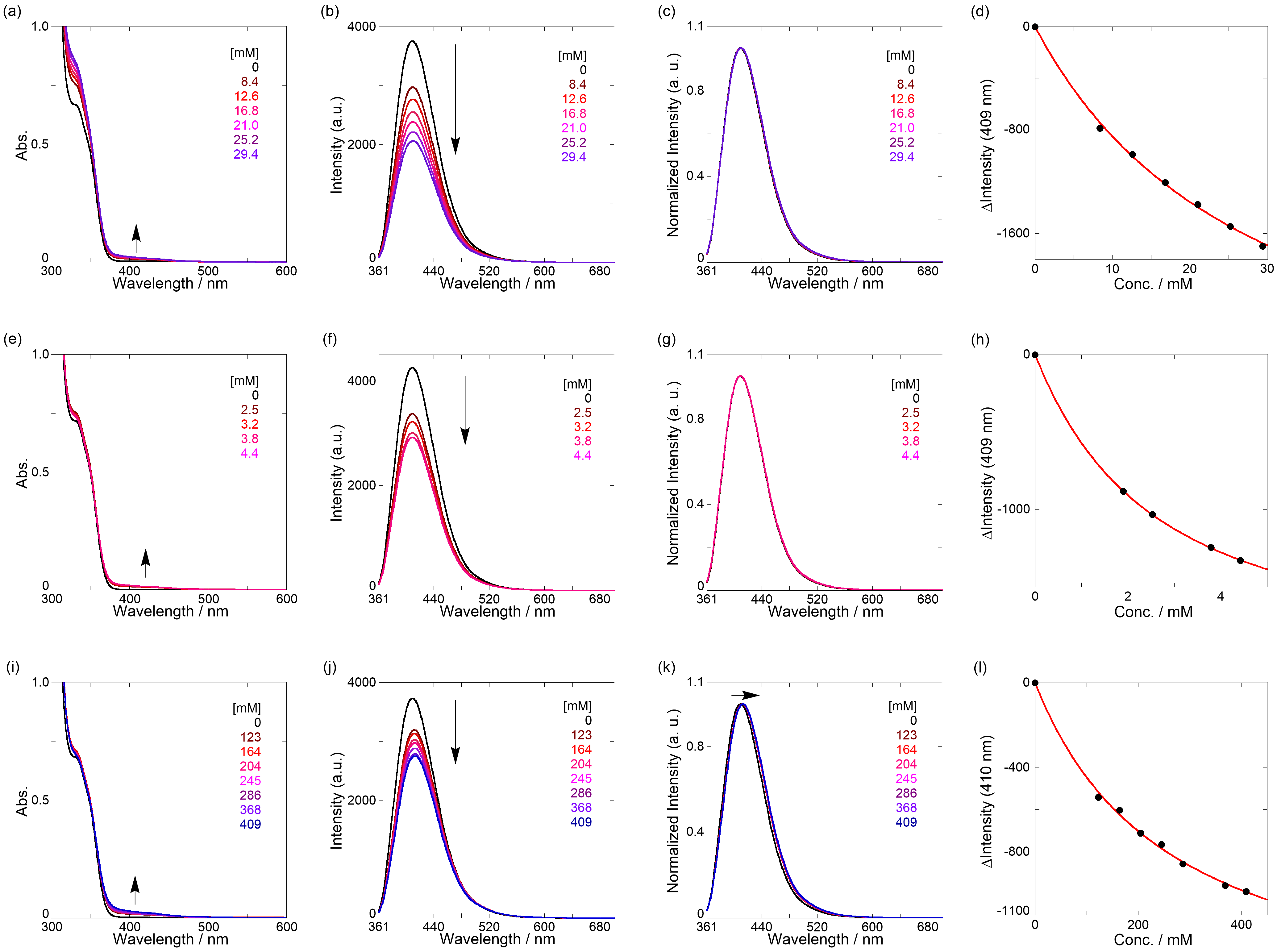


**Figure S24.** (a,e,i) UV/vis absorption and (b,f,j) fluorescence (λ_ex_: 351 nm) spectra of **SC** (a,b: 420 μM, e,f: 450 μM, i,j: 430 μM; black) following the addition of (a,b) testosterone (8.4–29.4 mM; from brown to purple), (e,f) corticosterone (2.5–4.4 mM; from brown to pink), or (i,j) allylestrenol (123–409 mM; from brown to blue) in CH_2_Cl_2_ at 25 °C; measurements were conducted in a 1 mm cell. The excitation wavelength at which comparable absorbances were obtained was selected. (c,g,k) Normalized fluorescence spectra of (b), (f), or (j). (d,h,l) Non-linear least-squares fittings, assuming the 1:1 stoichiometry with **SC** and (d) testosterone, (h) corticosterone, or (l) allylestrenol monitored at (d,h) 409 or (l) 410 nm, to determine the binding constants at 25 °C.

**Analyses for Supramolecular Polymerization**


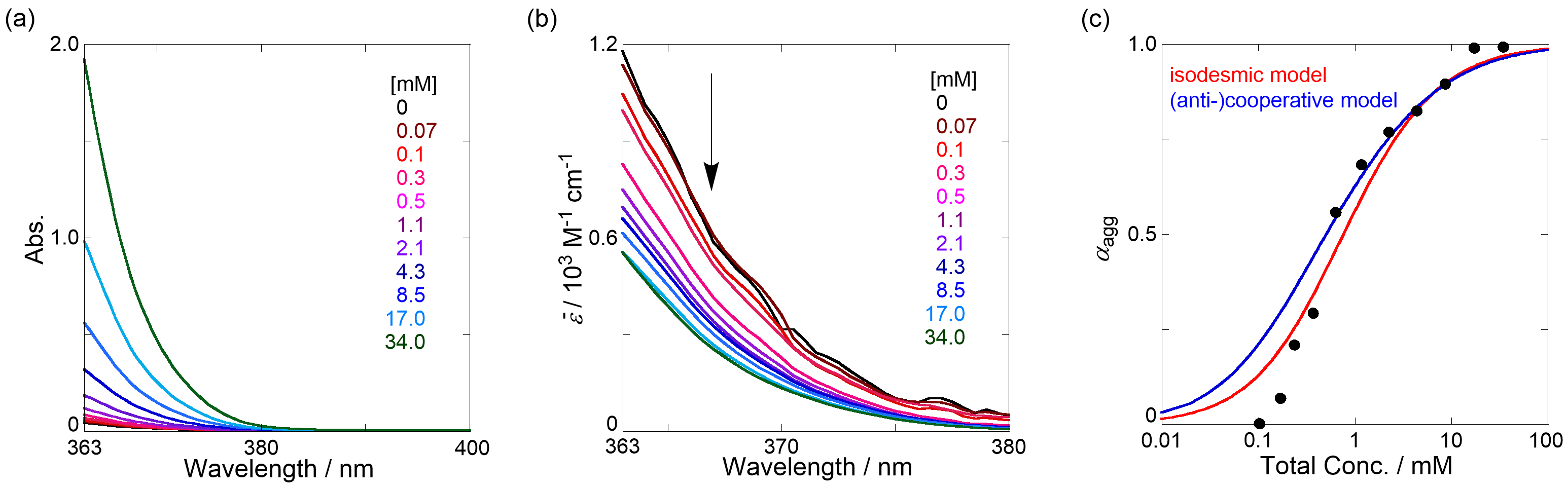


**Figure S25.** (a) UV/vis absorption and (b) molar extinction coefficient spectra of **SC** (128 μM; black) following the addition of **sumanene** (0.07–34.0 mM; from brown to green) in CH_2_Cl_2_ at 25 °C; measurements were conducted in a 1 mm cell. (c) Plots of α_agg_ of **SC**•(**sumanene**)_n_ hetero-supramolecular polymer monitored at 363 nm in (b) as a function of *C*_t_; the red and blue lines represent the fitting results based on the isodesmic and (anti-)cooperative model, respectively.

| **Table S5.** Equilibrium constants of **SC•**(**sumanene**)_n_ hetero-supramolecular polymerization in CH_2_Cl_2_ at 25 °C based on the isodesmic and anti-cooperative models | | | |
| --- | --- | --- | --- |
| fitting model | *K*_i_ or *K*_ac_ / M^-1^ | σ | correlation coefficient |
| isodesmic | 770 ± 120 | 1 | 0.978 |
| anti-cooperative | 540 ± 100 | 2.92 ± 1.26 | 0.993 |

**Discussion**

As shown in Figure 2a in the main text, **SC** and **sumanene** have different molar extinction coefficients that are not suitable to directly analyze hetero-supramolecular polymerization between **SC** and **sumanene**. Therefore, the molar extinction coefficients of the **sumanene** skeleton were extracted from those of **SC**, and then analyzed based on the assumption that all chemical species in solution composed of **sumanene**. Thus, *ε*_SC_ (Figure 2a, black solid line), *ε*_sumanene,(exp.)_ (Figure 2a, green dotted line), and *ε*_ref_ (Figure 2a, purple dotted line) were calculated from dilute solution of each compound, from which *ε*_sumanene-ref_ (Figure 2a, red solid line) can be calculated based on the equation S2. Then, *ε*_sumanene,(calcd.)_ in Figure S25b (black line) was re-calculated using the above-mentioned values of *ε*_ref_, *ε*_sumanene-ref_, and *ε*_SC_. The formation of supramolecular polymers was analyzed, according to our previous report^[[6]](#footnote-7)^.

$$\varepsilon_{SC}=\varepsilon_{Sumanene}+3\varepsilon_{ref}+\varepsilon_{sumanene-ref} (S2)$$

**
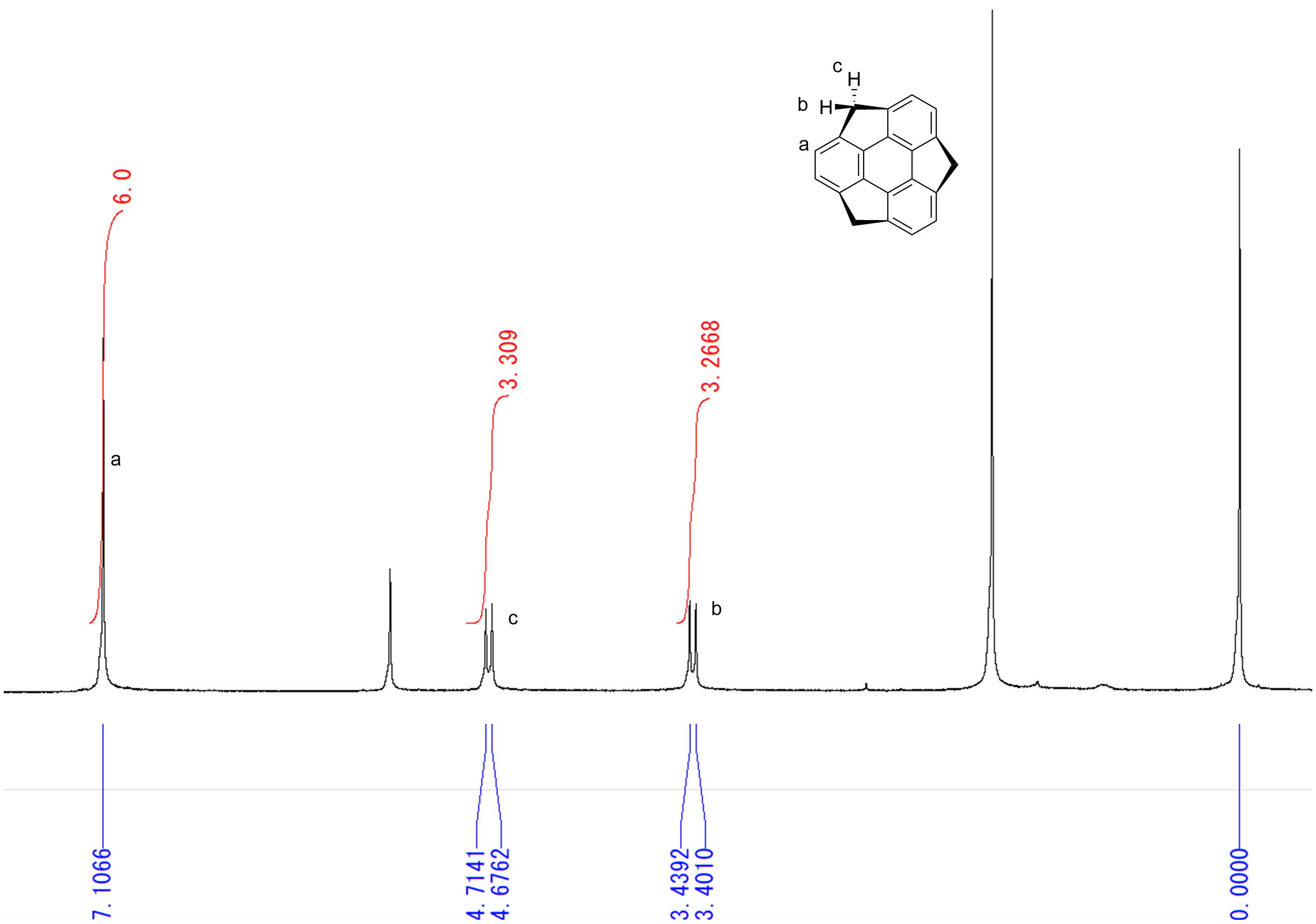
**

**Figure S26.** ^1^H NMR spectrum of **sumanene** in CD_2_Cl_2_ (9.16 mM) at room temperature for DOSY measurement.


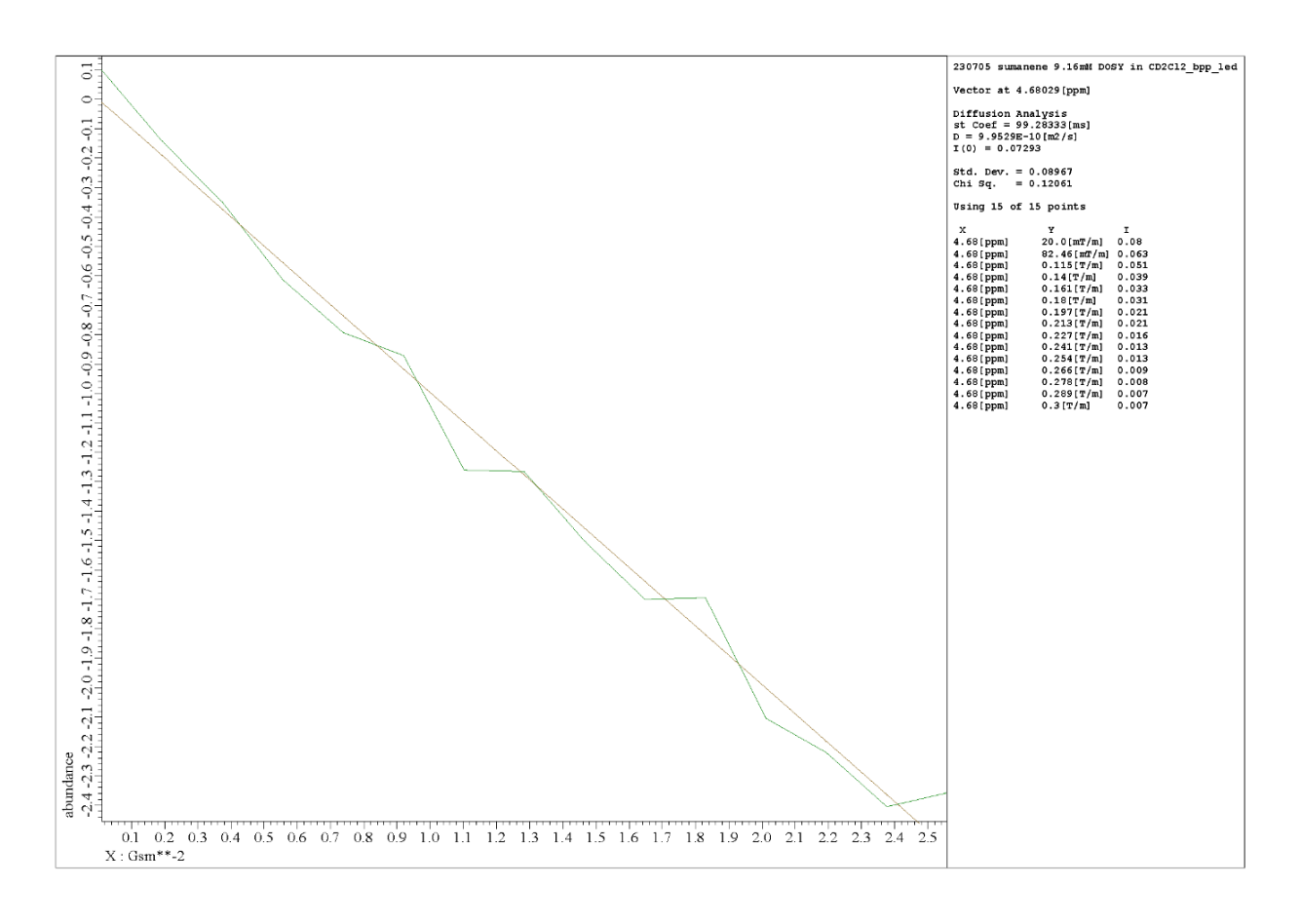


**Figure S27.** Diffusion analysis at 4.68 ppm of **sumanene** in CD_2_Cl_2_ (9.16 mM) at room temperature (23.7 °C, *D* = 9.95 × 10^-10^ m^2^/s).


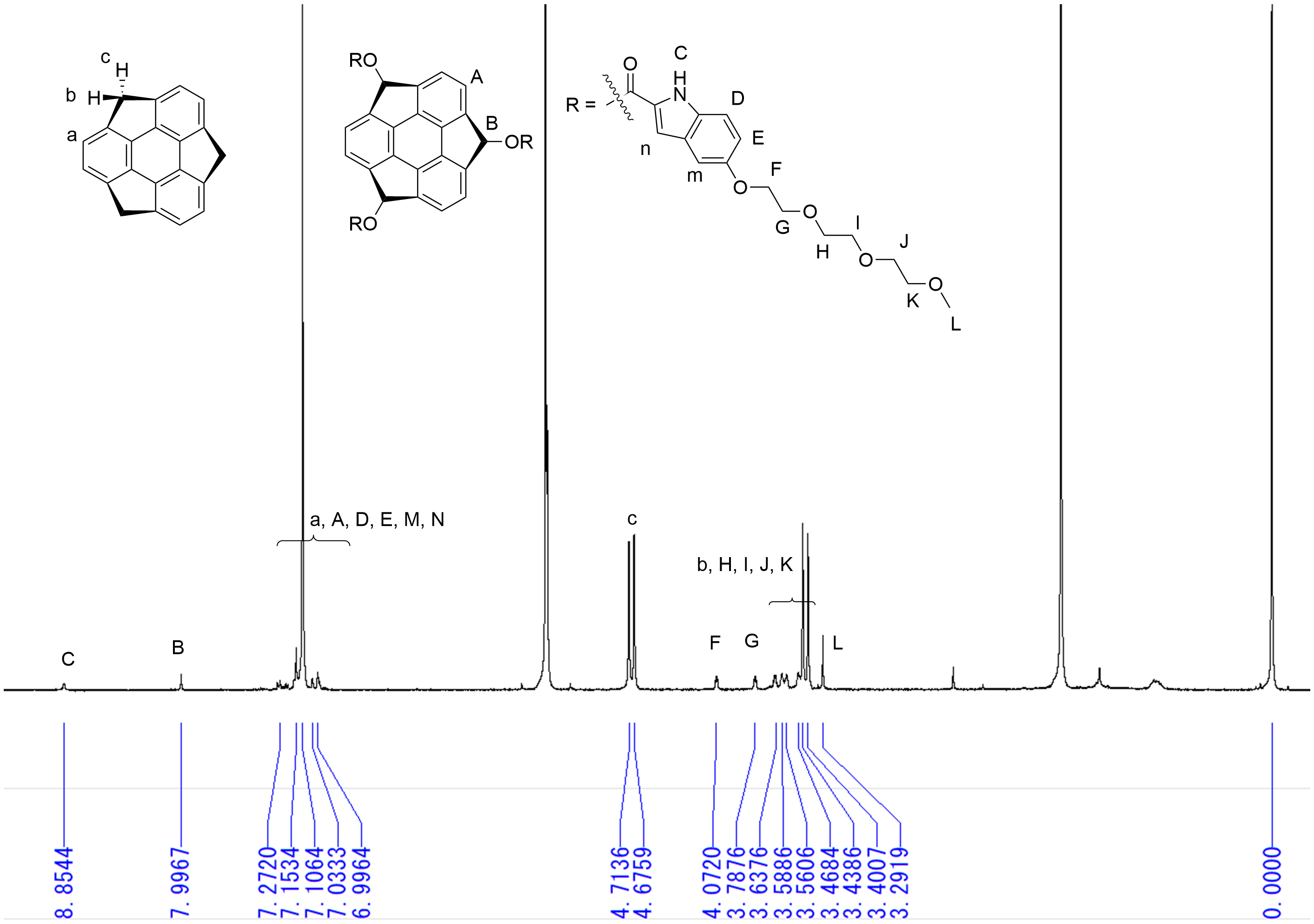


**Figure S28.** ^1^H NMR spectrum of **SC** (447 μM) and **sumanene** (9.09 mM) in CD_2_Cl_2_ at room temperature for DOSY measurement.


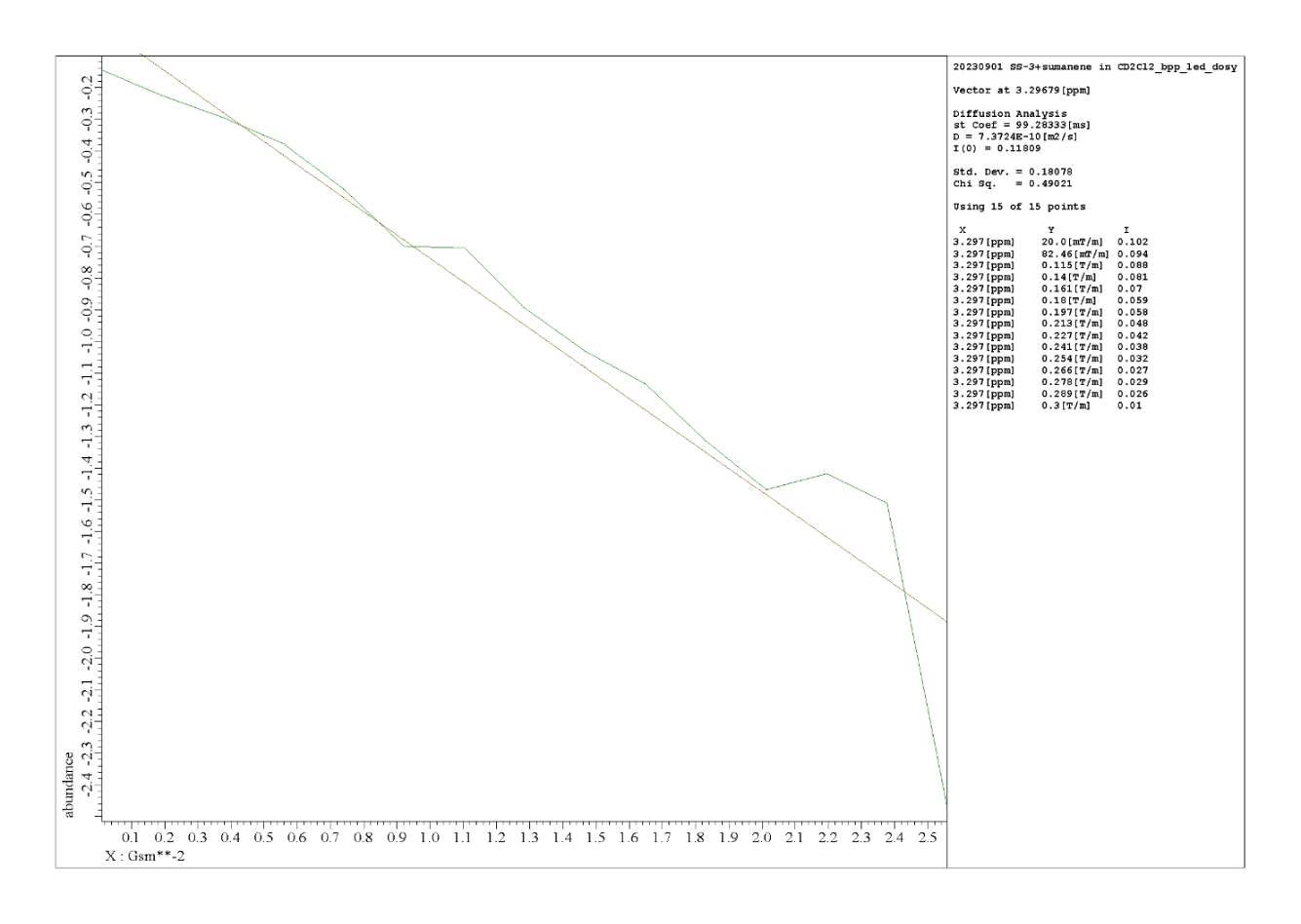


**Figure S29.** Diffusion analysis at 3.30 ppm of **SC** (447 μM) and **sumanene** (9.09 mM) in CD_2_Cl_2_ at room temperature (23.7 °C, *D* = 7.37 × 10^-10^ m^2^/s).

**Discussion**

In the DOSY measurements, the sizes of the supramolecular polymers were estimated by the ellipsoid approximations of equations S3 and S4^[[7]](#footnote-8)^. Since over 95% **sumanene** in the heteromer mixture containing **SC** and **sumanene** existed, the radius of the bowl of **sumanene** (4.1 Å)^6^ was used as the length of the short axis for all ellipsoid approximations:

$$D= \frac{k_{b}T}{6\pi\eta b}f\left( \rho\right) (S3)$$

$$f\left( \rho\right)=\frac{ln(\rho+\sqrt{\rho^{2}-1})}{\sqrt{\rho^{2}-1}} (S4)$$

where *D* represents diffusion coefficient, *k*_b_ is Boltzmann constant, *T* is temperature, *η* is viscosity (= 0.482 mPa^[[8]](#footnote-9)^), *a* is long axis of ellipsoid, *b* is short axis of ellipsoid, *ρ* (= *a*/*b*) is aspect ratio, respectively.

| **Table S6.** Analyses for **SC**•(**sumanene**)_n_ hetero-supramolecular polymerization based on DOSY and UV measurements | | | | | | |
| --- | --- | --- | --- | --- | --- | --- |
| system | concentration / mM | | | DOSY | | UV |
|  | **SC** | **sumanene** | total | 2*a* / Å | DP | DP |
| **sumanene** | 0 | 9.16 | 9.16 | 10.8 | 3 | 2.9 |
| **SC**•(**sumanene**)_n_ | 0.45 | 9.09 | 9.54 | 21.3 | 4–5*^a,b^* | 3.3*^a^* |
| *^a^*DP in **SC**•(**sumanene**)_n_ system means 1 × **SC** + *n* × **sumanene**; DP = 5 means 1 × **SC** + 4 × **sumanene**. *^b^*A long axis of the ellipsoid was calculated without containing the triethylene glycol chains in **SC** due to the flexible moiety. | | | | | | |

**Sensing Data of SC•(sumanene)_n_**


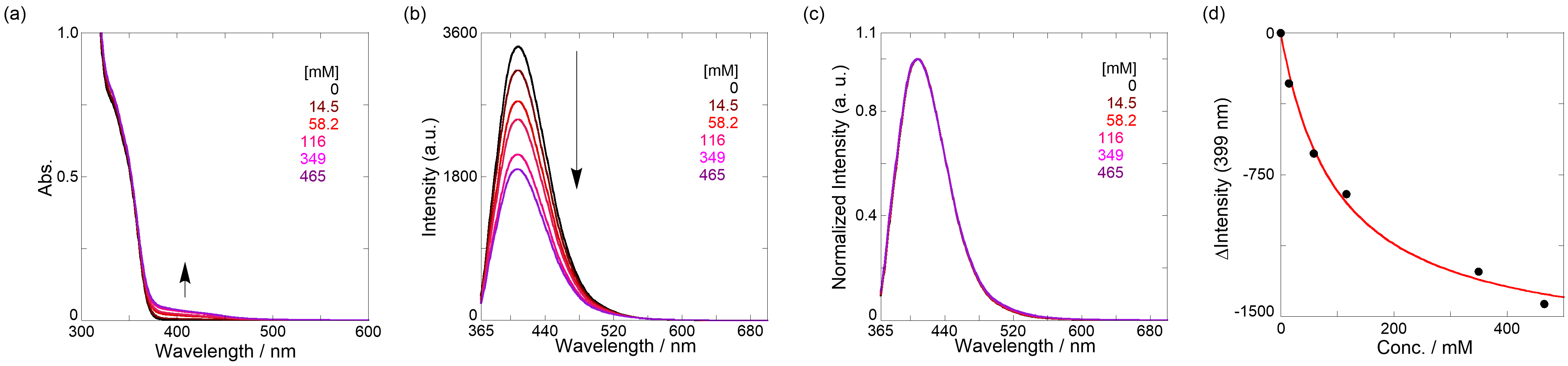


**Figure S30.** (a) UV/vis absorption and (b) fluorescence (λ_ex_: 355 nm) spectra of **SC** (432 μM) with **sumanene** (552 μM, DP: 1.5, black) following the addition of **MB** (14.5–465 mM; from brown to purple) in CH_2_Cl_2_ at 25 °C; measurements were conducted in a 1 mm cell. The excitation wavelength at which comparable absorbances were obtained was selected. (c) Normalized fluorescence spectra of (b). (d) Non-linear least-squares fitting, assuming the 1:1 stoichiometry with **SC**•(**sumanene**)_n_ and **MB** monitored at 399 nm, to determine the binding constant as 10 ± 1 M^-1^ at 25 °C.


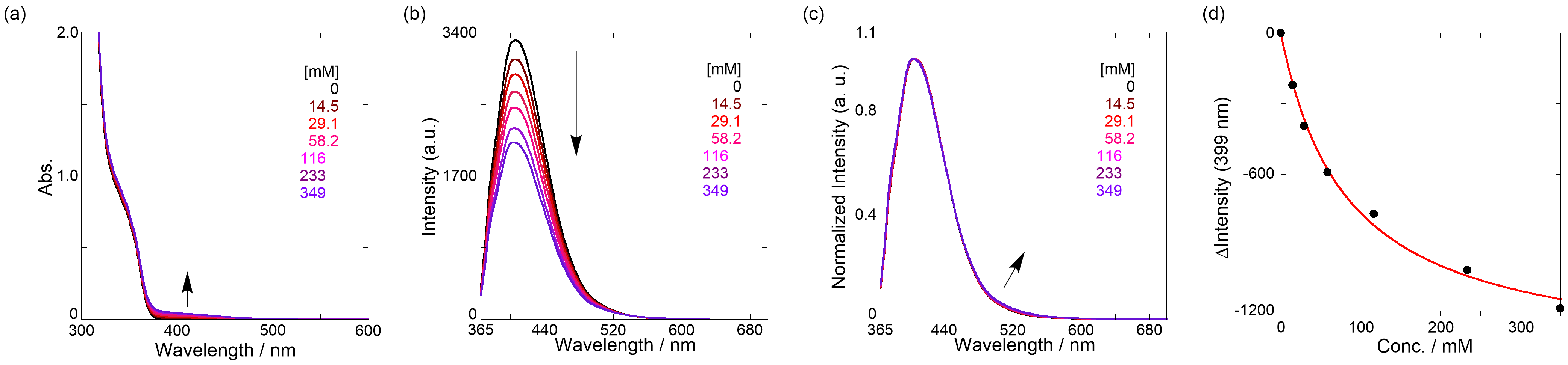


**Figure S31.** (a) UV/vis absorption and (b) fluorescence (λ_ex_: 355 nm) spectra of **SC** (468 μM) with **sumanene** (2.06 mM, DP: 2.0, black) following the addition of **MB** (14.5–349 mM; from brown to violet) in CH_2_Cl_2_ at 25 °C; measurements were conducted in a 1 mm cell. The excitation wavelength at which comparable absorbances were obtained was selected. (c) Normalized fluorescence spectra of (b). (d) Non-linear least-squares fitting, assuming the 1:1 stoichiometry with **SC**•(**sumanene**)_n_ and **MB** monitored at 399 nm, to determine the binding constant as 12 ± 1 M^-1^ at 25 °C.


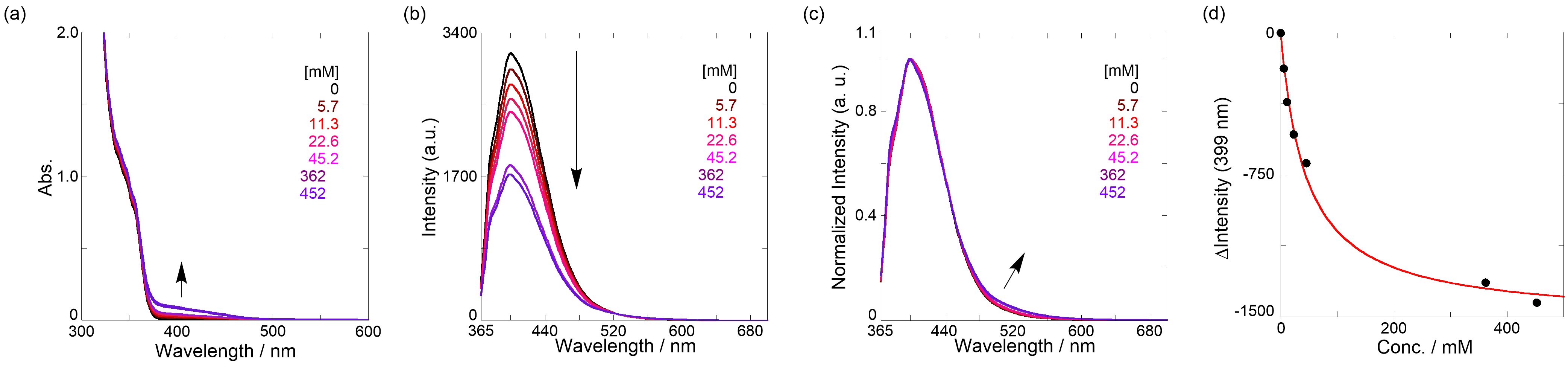


**Figure S32.** (a) UV/vis absorption and (b) fluorescence (λ_ex_: 355 nm) spectra of **SC** (482 μM) with **sumanene** (4.43 mM, DP: 2.5, black) following the addition of **MB** (5.7–452 mM; from brown to violet) in CH_2_Cl_2_ at 25 °C; measurements were conducted in a 1 mm cell. The excitation wavelength at which comparable absorbances were obtained was selected. (c) Normalized fluorescence spectra of (b). (d) Non-linear least-squares fitting, assuming the 1:1 stoichiometry with **SC**•(**sumanene**)_n_ and **MB** monitored at 399 nm, to determine the binding constant as 22 ± 3 M^-1^ at 25 °C.


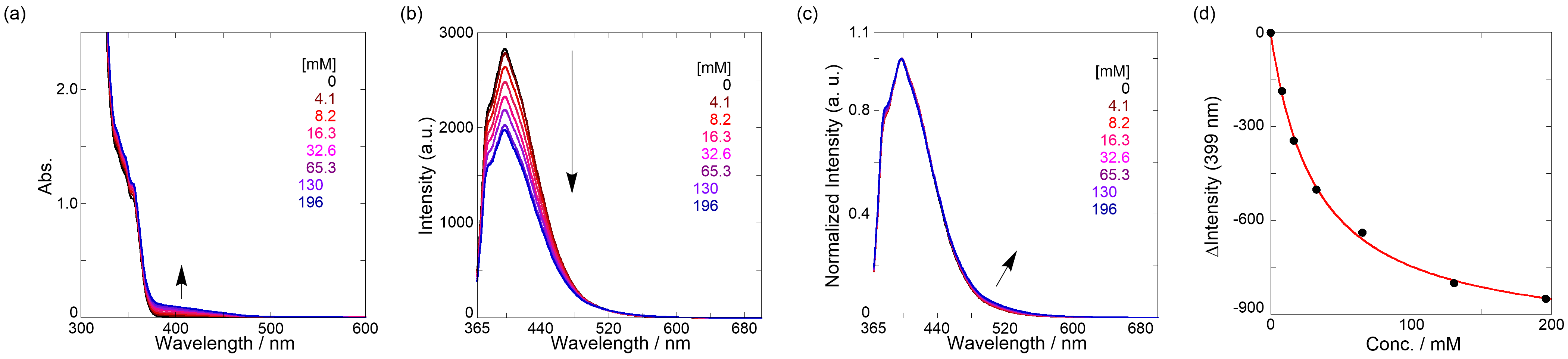


**Figure S33.** (a) UV/vis absorption and (b) fluorescence (λ_ex_: 355 nm) spectra of **SC** (470 μM) with **sumanene** (7.28 mM, DP: 3.0, black) following the addition of **MB** (4.1–196 mM; from brown to navy) in CH_2_Cl_2_ at 25 °C; measurements were conducted in a 1 mm cell. The excitation wavelength at which comparable absorbances were obtained was selected. (c) Normalized fluorescence spectra of (b). (d) Non-linear least-squares fitting, assuming the 1:1 stoichiometry with **SC**•(**sumanene**)_n_ and **MB** monitored at 399 nm, to determine the binding constant as 31 ± 2 M^-1^ at 25 °C.


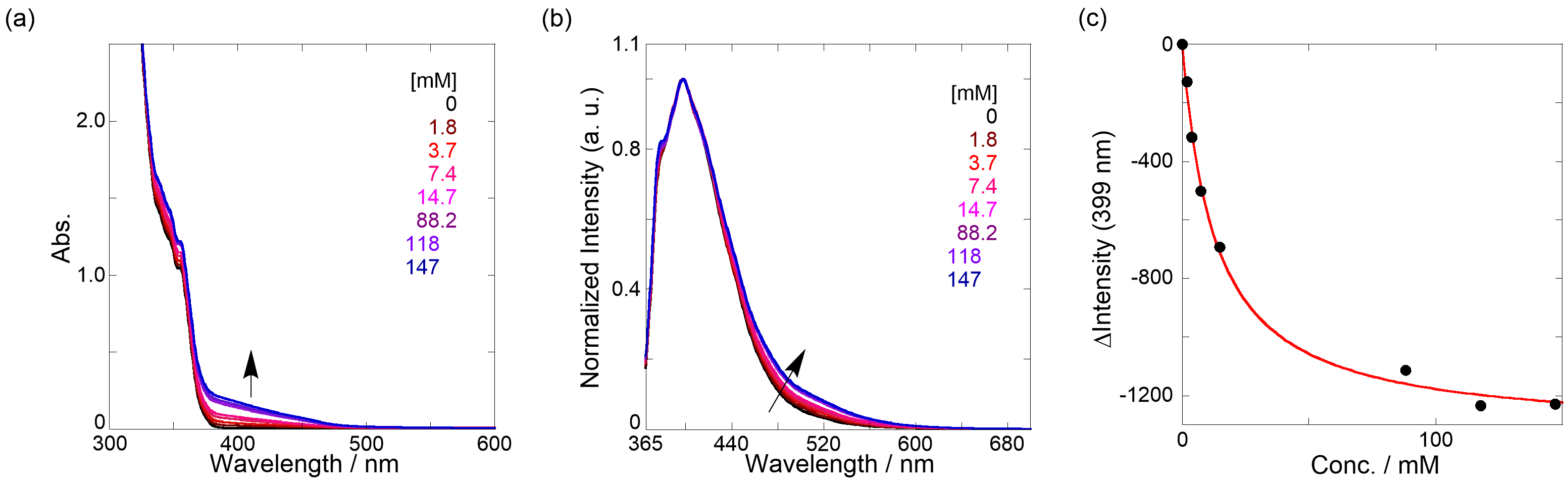


**Figure S34.** (a) UV/vis spectra absorption of **SC** (445 μM) with **sumanene** (8.87 mM, DP: 3.2, black) following the addition of **MB** (1.8–147 mM; from brown to navy) in CH_2_Cl_2_ at 25 °C; measurements were conducted in a 1 mm cell. (b) Normalized fluorescence spectra of Figure 5a in the main text. (c) Non-linear least-squares fitting, assuming the 1:1 stoichiometry with **SC**•(**sumanene**)_n_ and **MB** monitored at 399 nm, to determine the binding constant at 25 °C.


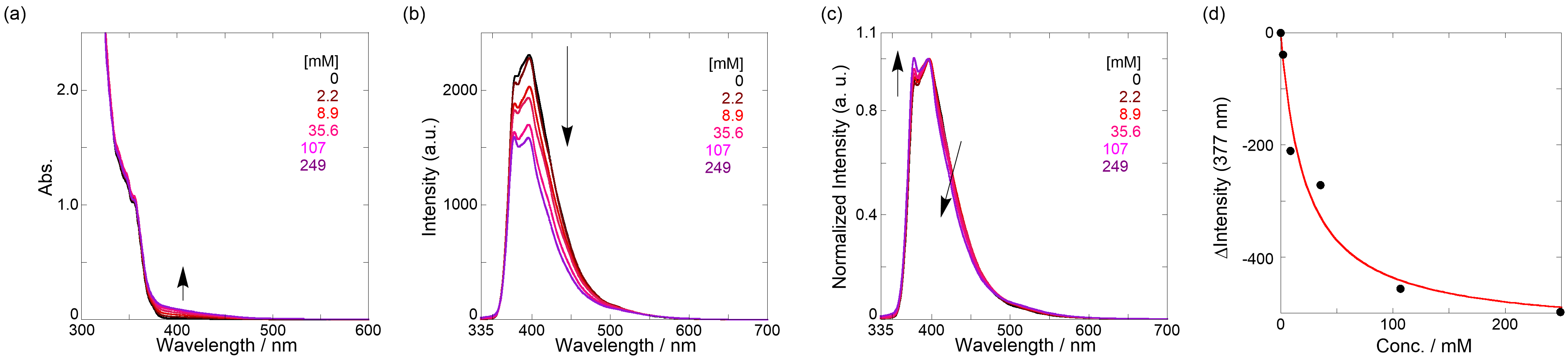


**Figure S35.** (a) UV/vis absorption and (b) fluorescence (λ_ex_: 325 nm) spectra of **SC** (402 μM) with **sumanene** (8.72 mM, DP: 3.2, black) following the addition of **DI** (2.2–249 mM, from brown to purple) in CH_2_Cl_2_ at 25 °C; measurements were conducted in a 1 mm cell. The excitation wavelength at which comparable absorbances were obtained was selected. (c) Normalized fluorescence spectra of (b). (d) Non-linear least-squares fitting, assuming the 1:1 stoichiometry with **SC**•(**sumanene**)_n_ and **DI** monitored at 377 nm, to determine the binding constant at 25 °C.


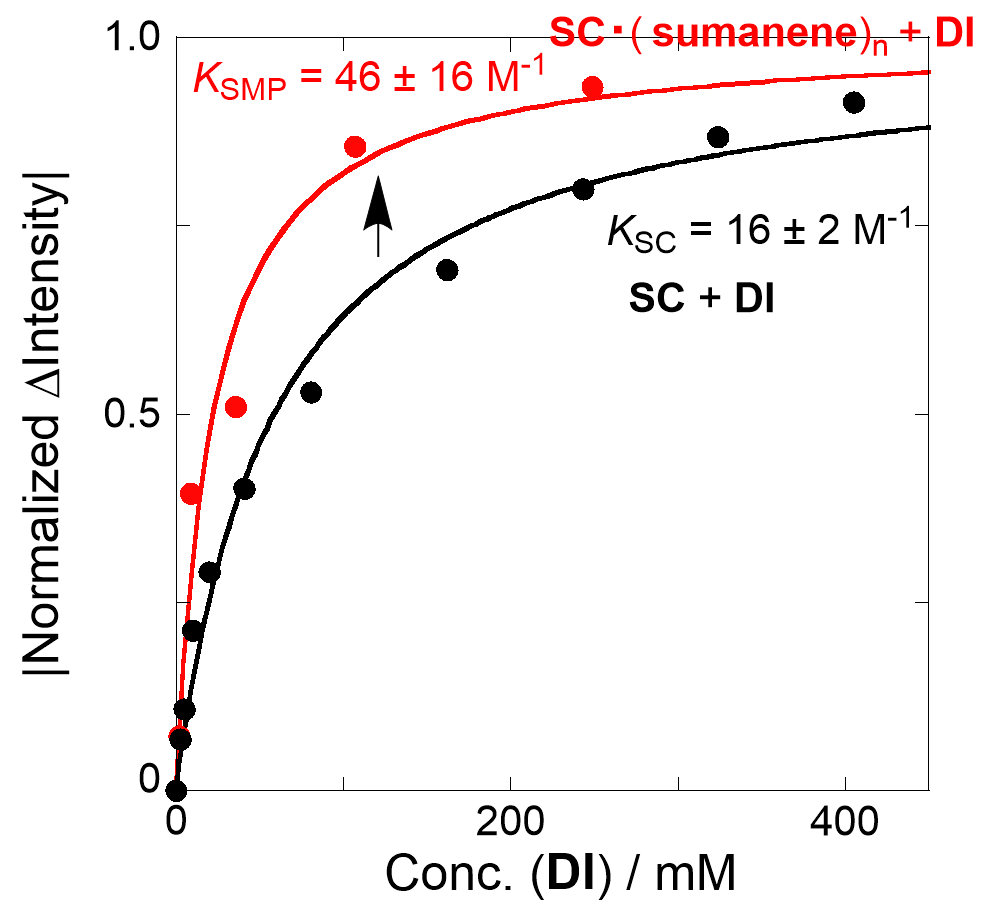


**Figure S36.** Normalized binding isotherms of **SC**•(**sumanene**)_n_ (red) and **SC** (black) following the addition of **DI** at 25 °C.


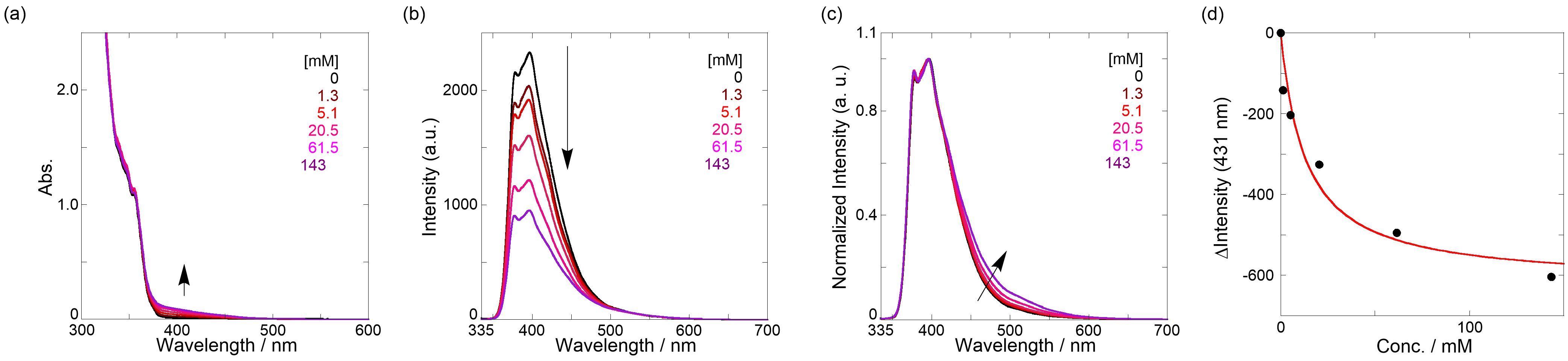


**Figure S37.** (a) UV/vis absorption and (b) fluorescence (λ_ex_: 325 nm) spectra of **SC** (422 μM) with **sumanene** (9.02 mM, DP: 3.2, black) following the addition of **TT** (1.3–143 mM; from brown to purple) in CH_2_Cl_2_ at 25 °C; measurements were conducted in a 1 mm cell. The excitation wavelength at which comparable absorbances were obtained was selected. (c) Normalized fluorescence spectra of (b). (d) Non-linear least-squares fitting, assuming the 1:1 stoichiometry with **SC**•(**sumanene**)_n_ and **TT** monitored at 431 nm, to determine the binding constant at 25 °C.


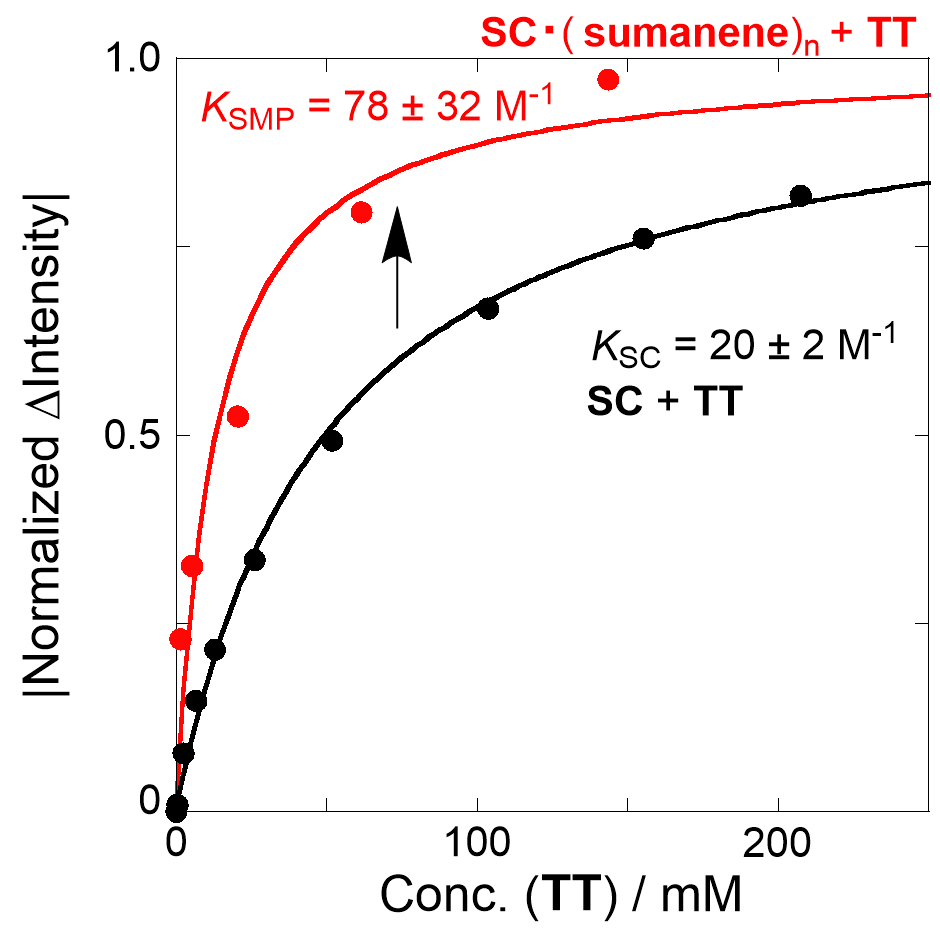


**Figure S38.** Normalized binding isotherms of **SC**•(**sumanene**)_n_ (red) and **SC** (black) following the addition of **TT** at 25 °C.


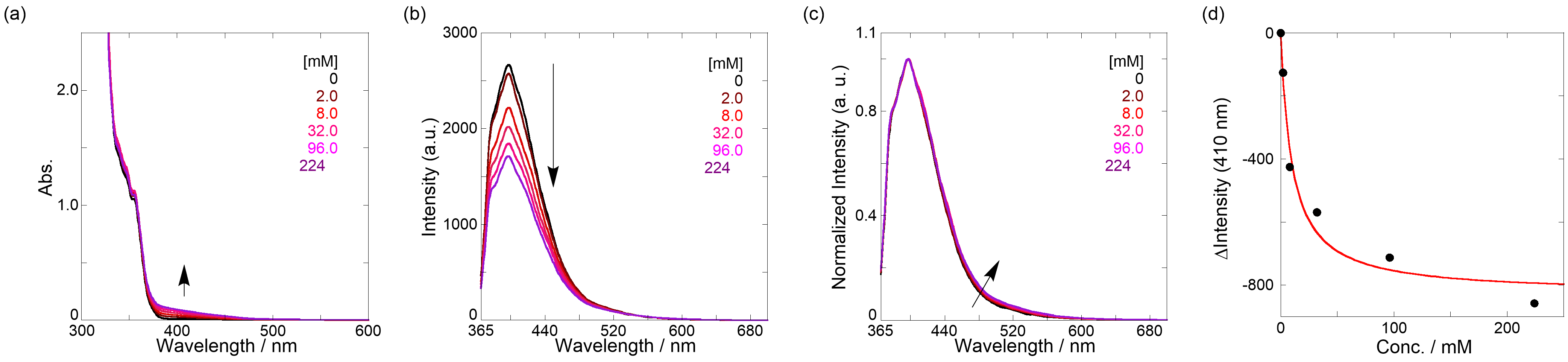


**Figure S39.** (a) UV/vis absorption and (b) fluorescence (λ_ex_: 355 nm) spectra of **SC** (395 μM) with **sumanene** (8.99 mM, DP: 3.2, black) following the addition of **TC** (2.0–224 mM; from brown to purple) in CH_2_Cl_2_ at 25 °C; measurements were conducted in a 1 mm cell. The excitation wavelength at which comparable absorbances were obtained was selected. (c) Normalized fluorescence spectra of (b). (d) Non-linear least-squares fitting, assuming the 1:1 stoichiometry with **SC**•(**sumanene**)_n_ and **TC** monitored at 410 nm, to determine the binding constant at 25 °C.


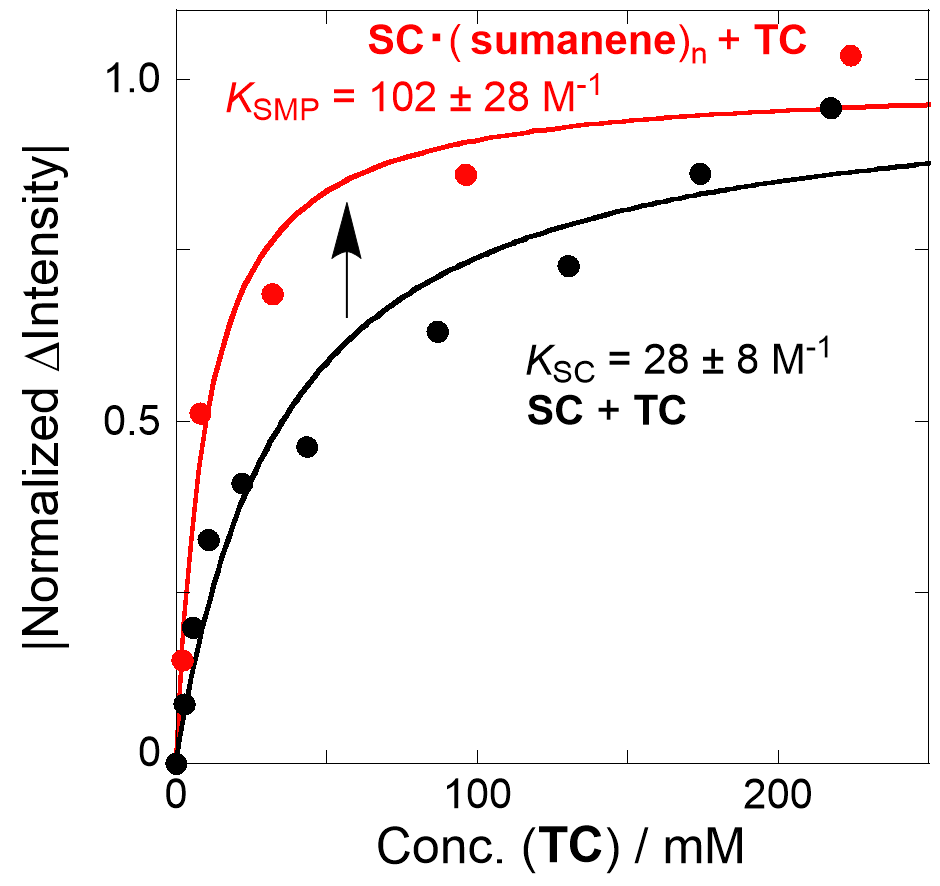


**Figure S40.** Normalized binding isotherms of **SC**•(**sumanene**)_n_ (red) and **SC** (black) following the addition of **TC** at 25 °C.


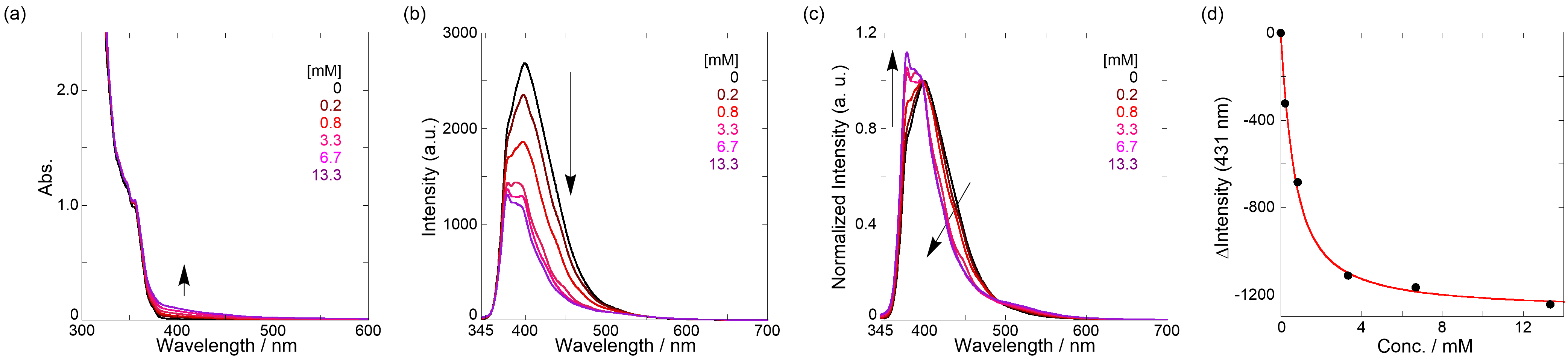


**Figure S41.** (a) UV/vis absorption and (b) fluorescence (λ_ex_: 335 nm) spectra of **SC** (405 μM) with **sumanene** (8.64 mM, DP: 3.2, black) following the addition of **TBPB** (0.2–13.3 mM; from brown to purple) in CH_2_Cl_2_ at 25 °C; measurements were conducted in a 1 mm cell. The excitation wavelength at which comparable absorbances were obtained was selected. (c) Normalized fluorescence spectra of (b). (d) Non-linear least-squares fitting, assuming the 1:1 stoichiometry with **SC**•(**sumanene**)_n_ and **TBPB** monitored at 431 nm, to determine the binding constant at 25 °C.


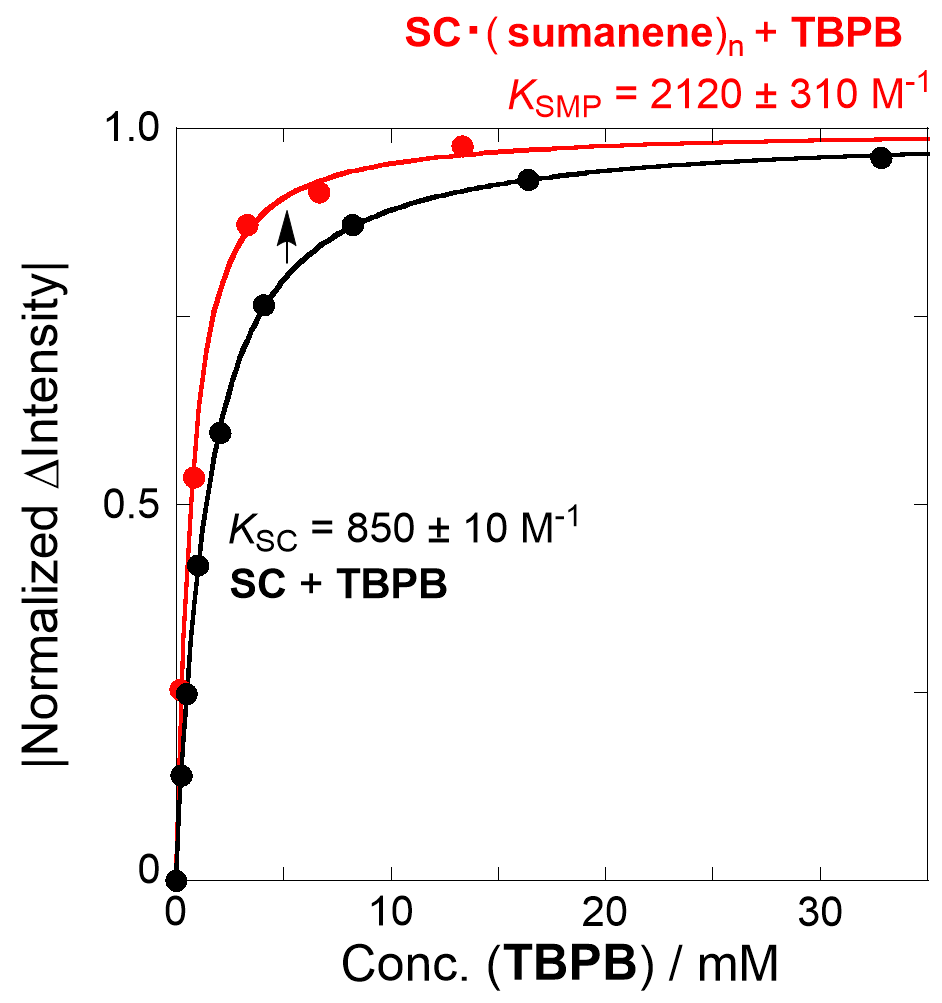


**Figure S42.** Normalized binding isotherms of **SC**•(**sumanene**)_n_ (red) and **SC** (black) following the addition of **TBPB** at 25 °C.


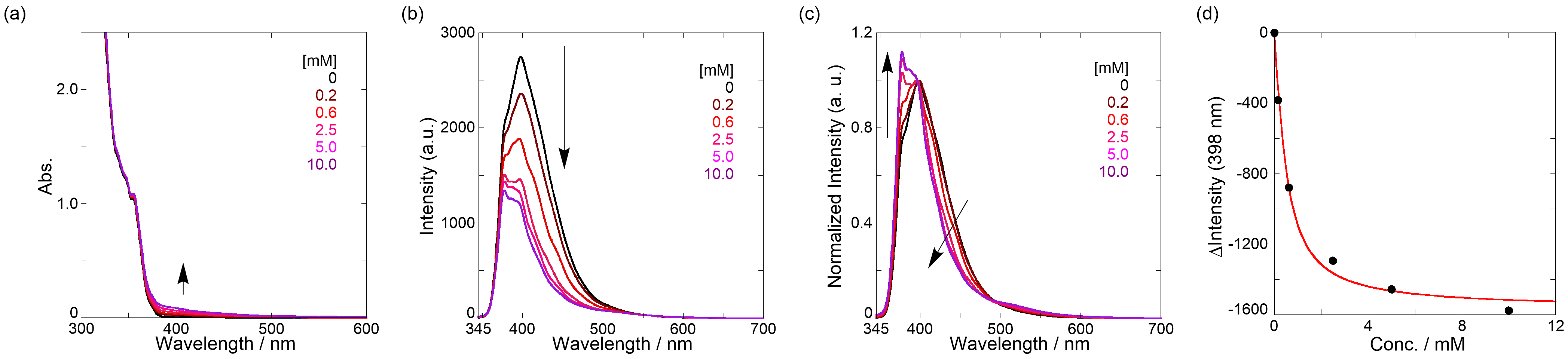


**Figure S43.** (a) UV/vis absorption and (b) fluorescence (λ_ex_: 335 nm) spectra of **SC** (394 μM) with **sumanene** (8.73 mM, DP: 3.2, black) following the addition of **TBPI** (0.2–10.0 mM; from brown to purple) in CH_2_Cl_2_ at 25 °C; measurements were conducted in a 1 mm cell. The excitation wavelength at which comparable absorbances were obtained was selected. (c) Normalized fluorescence spectra of (b). (d) Non-linear least-squares fitting, assuming the 1:1 stoichiometry with **SC**•(**sumanene**)_n_ and **TBPI** monitored at 398 nm, to determine the binding constant at 25 °C.


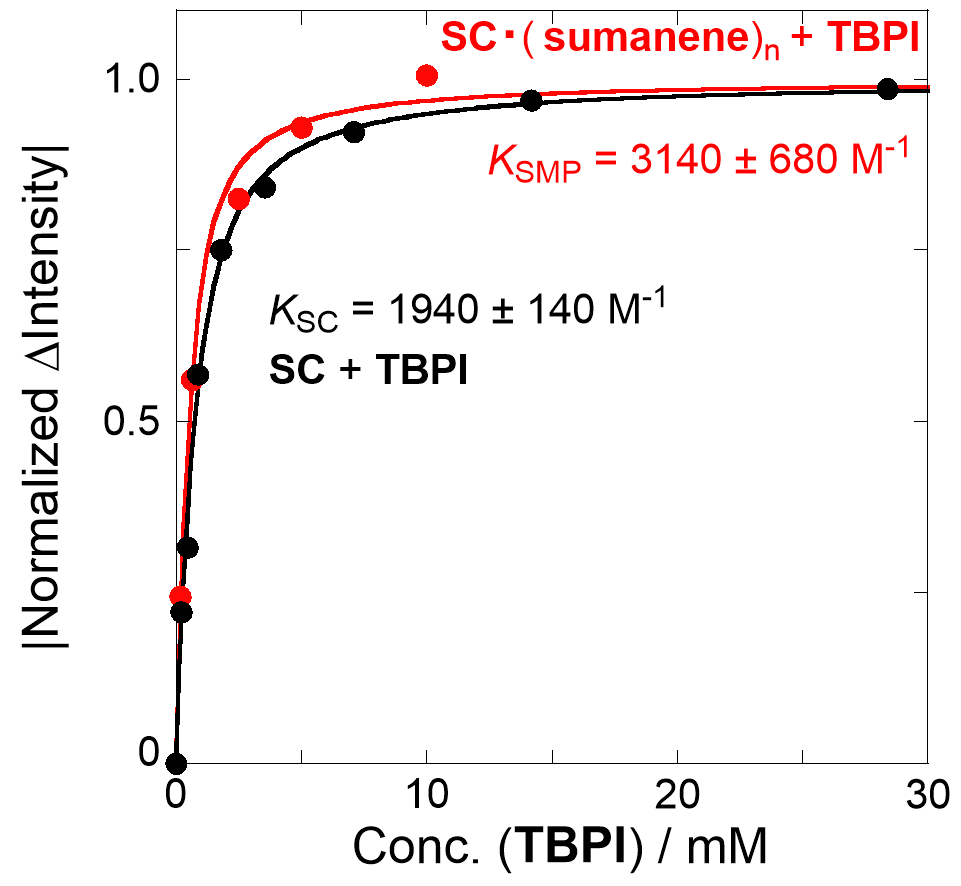


**Figure S44.** Normalized binding isotherms of **SC**•(**sumanene**)_n_ (red) and **SC** (black) following the addition of **TBPI** at 25 °C.


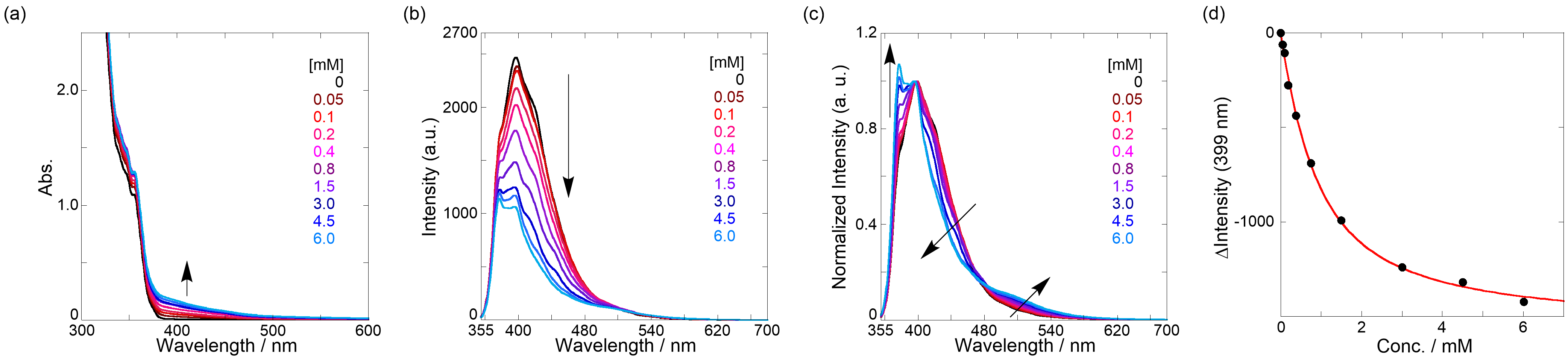


**Figure S45.** (a) UV/vis absorption and (b) fluorescence (λ_ex_: 345 nm) spectra of **SC** (450 μM) with **sumanene** (9.29 mM, DP: 3.3, black) following the addition of **TBPT** (0.05–6.0 mM; from brown to sky blue) in CH_2_Cl_2_ at 25 °C; measurements were conducted in a 1 mm cell. The excitation wavelength at which comparable absorbances were obtained was selected. (c) Normalized fluorescence spectra of (b). (d) Non-linear least-squares fitting, assuming the 1:1 stoichiometry with **SC**•(**sumanene**)_n_ and **TBPT** monitored at 399 nm, to determine the binding constant at 25 °C.


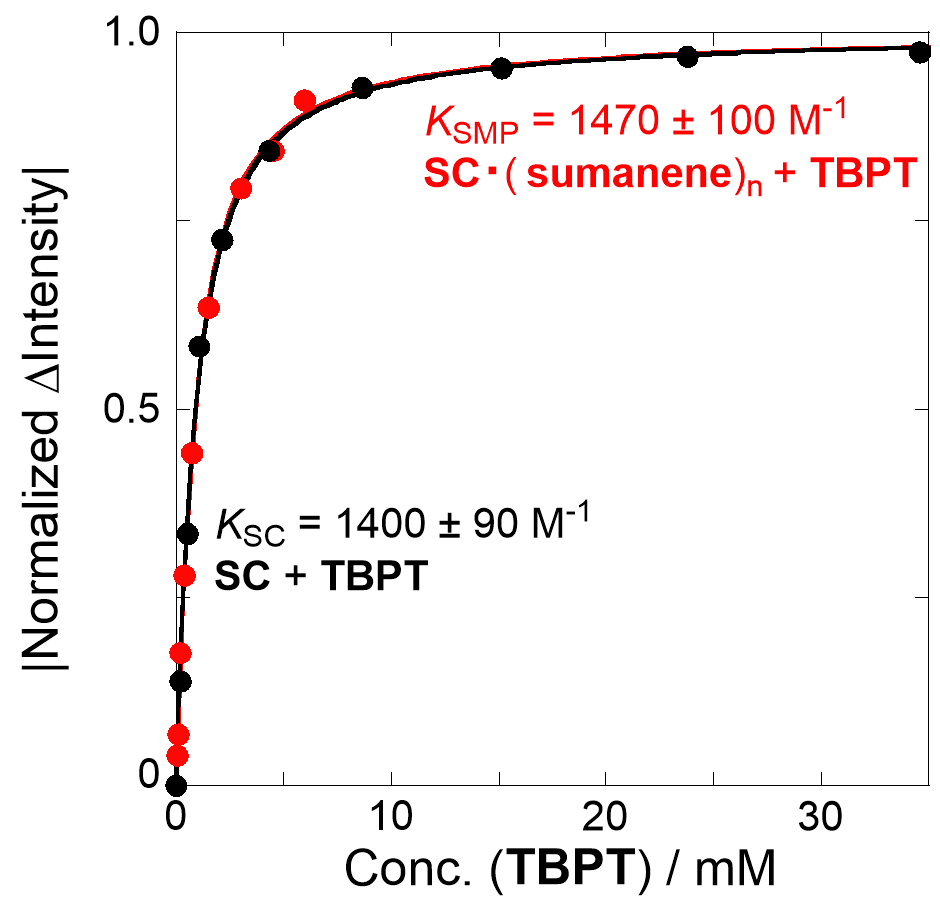


**Figure S46.** Normalized binding isotherms of **SC**•(**sumanene**)_n_ (red) and **SC** (black) following the addition of **TBPT** at 25 °C.


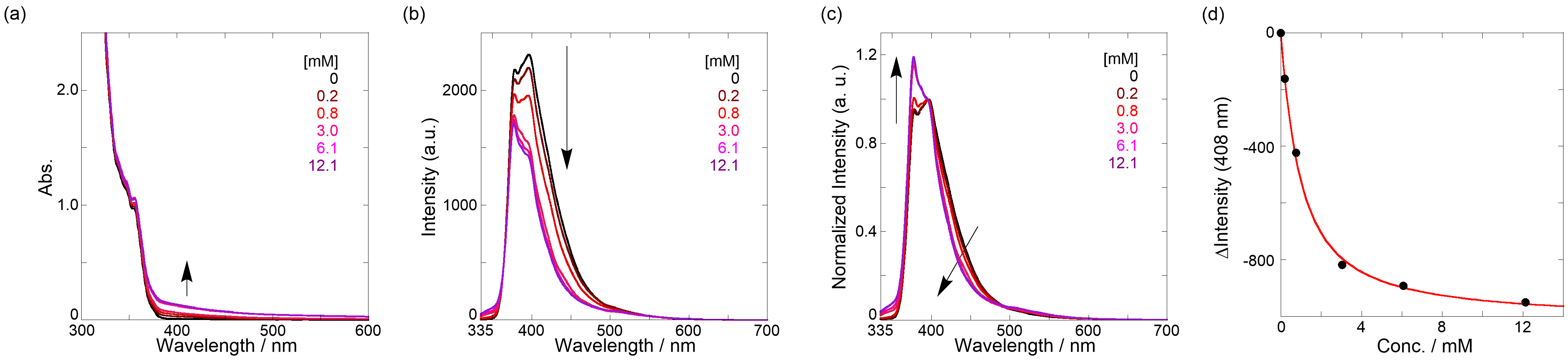


**Figure S47.** (a) UV/vis absorption and (b) fluorescence (λ_ex_: 325 nm) spectra of **SC** (397 μM) with **sumanene** (8.78 mM, DP: 3.2, black) following the addition of **TBPC** (0.2–12.1 mM; from brown to purple) in CH_2_Cl_2_ at 25 °C; measurements were conducted in a 1 mm cell. The excitation wavelength at which comparable absorbances were obtained was selected. (c) Normalized fluorescence spectra of (b). (d) Non-linear least-squares fitting, assuming the 1:1 stoichiometry with **SC**•(**sumanene**)_n_ and **TBPC** monitored at 408 nm, to determine the binding constant at 25 °C.


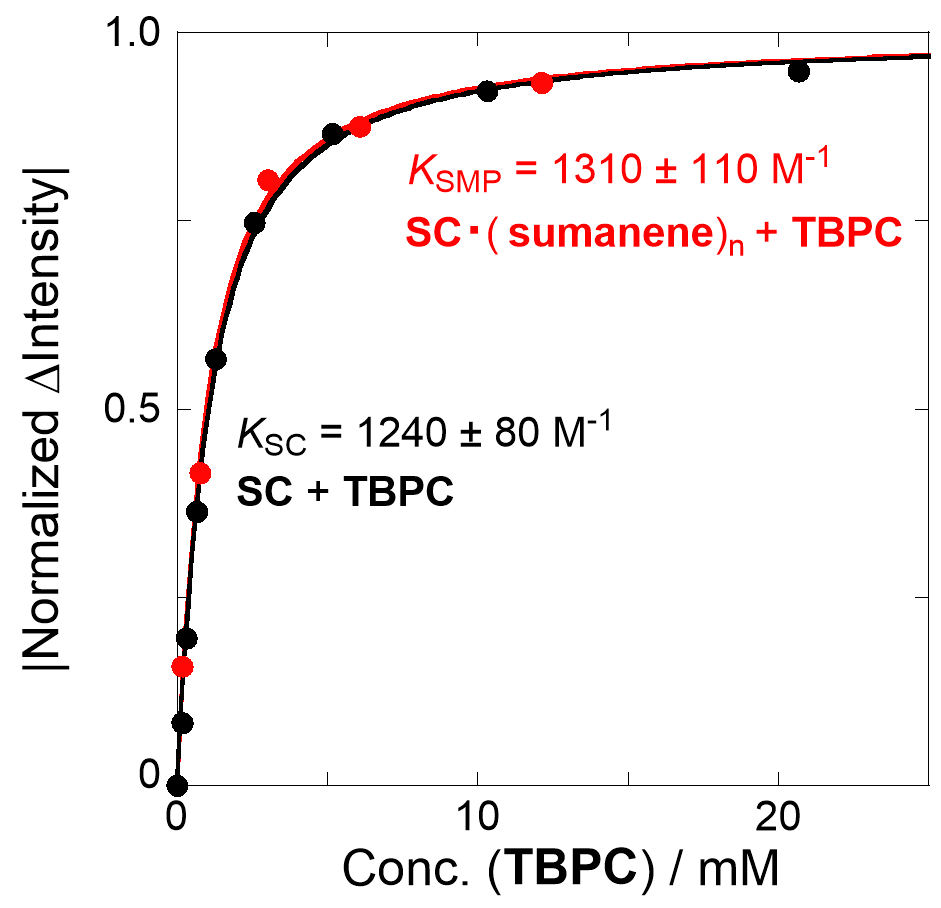


**Figure S48.** Normalized binding isotherms of **SC**•(**sumanene**)_n_ (red) and **SC** (black) following the addition of **TBPC** at 25 °C.

**Steroid Sensing by SC•(sumanene)_n_**


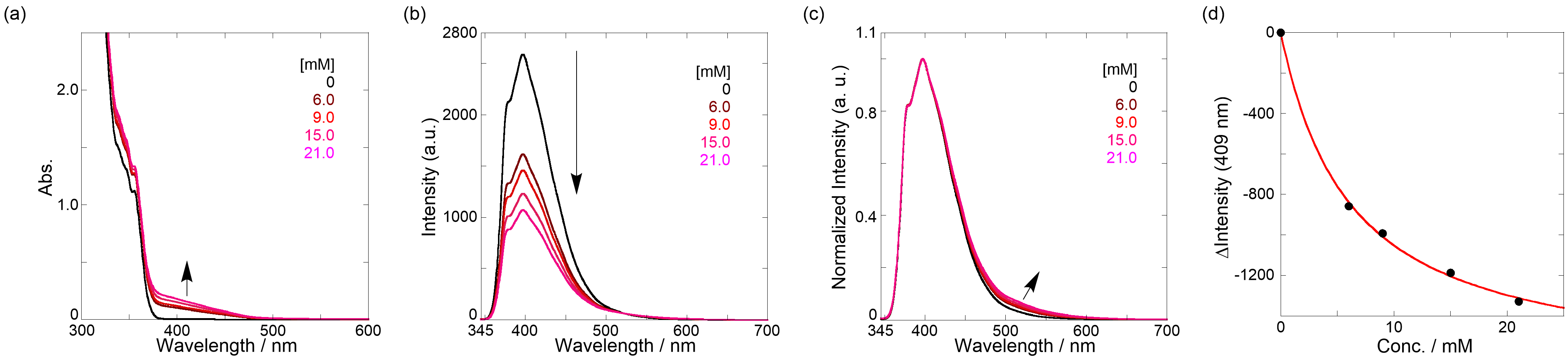


**Figure S49.** (a) UV/vis absorption and (b) fluorescence (λ_ex_: 335 nm) spectra of **SC** (437 μM) with **sumanene** (8.50 mM, DP: 3.2, black) following the addition of **testosterone** (6.0–21.0 mM; from brown to pink) in CH_2_Cl_2_ at 25 °C; measurements were conducted in a 1 mm cell. The excitation wavelength at which comparable absorbances were obtained was selected. (c) Normalized fluorescence spectra of (b). (d) Non-linear least-squares fitting, assuming the 1:1 stoichiometry with **SC**•(**sumanene**)_n_ and **testosterone** monitored at 409 nm, to determine the binding constant at 25 °C.


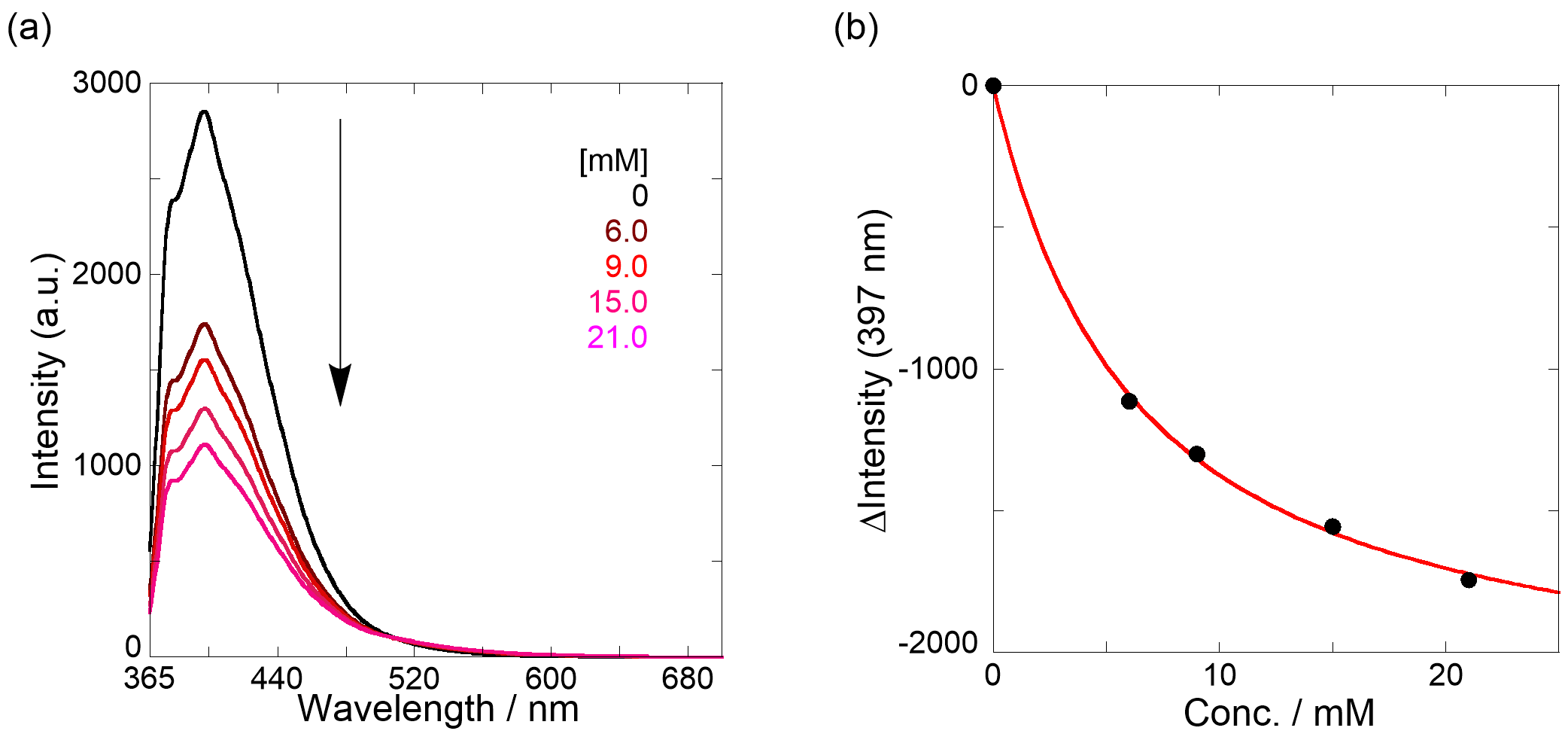


**Figure S49-2.** (a) Fluorescence (λ_ex_: 355 nm) spectra of **SC** (437 μM) with **sumanene** (8.50 mM, DP: 3.2, black) following the addition of **testosterone** (6.0–21.0 mM; from brown to pink) in CH_2_Cl_2_ at 25 °C in a 1 mm cell. (b) Non-linear least-squares fitting, assuming the 1:1 stoichiometry with **SC**•(**sumanene**)_n_ and **testosterone** monitored at 397 nm, to determine the binding constant as 170 ± 15 M^-1^ at 25 °C.


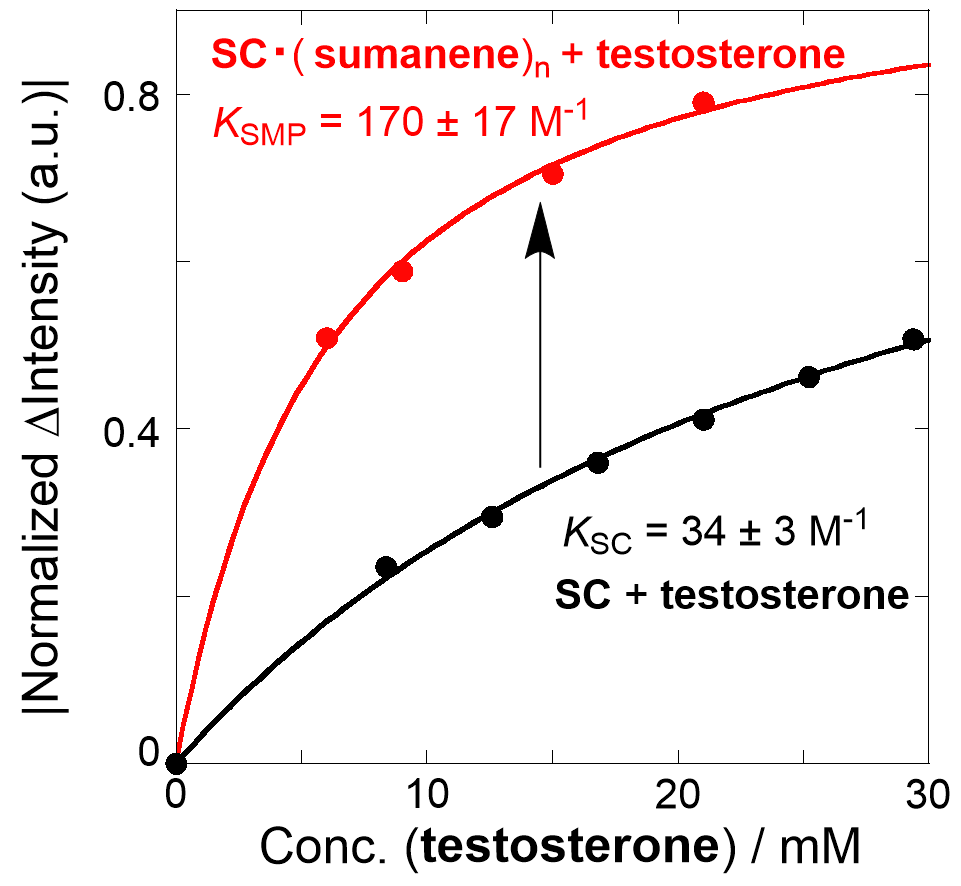


**Figure S50.** Normalized binding isotherms of **SC**•(**sumanene**)_n_ (red) and **SC** (black) following the addition of **testosterone** at 25 °C.


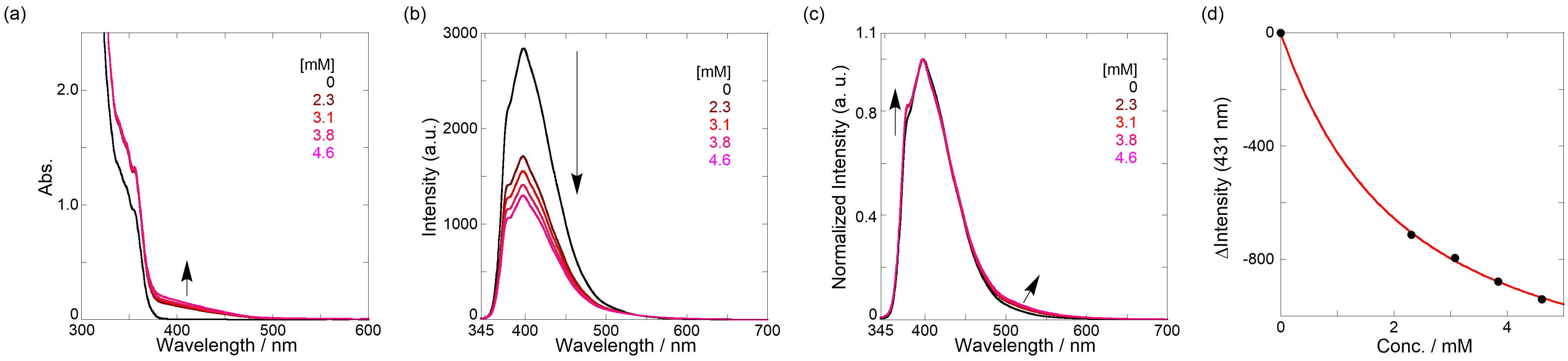


**Figure S51.** (a) UV/vis absorption and (b) fluorescence (λ_ex_: 335 nm) spectra of **SC** (434 μM) with **sumanene** (9.28 mM, DP: 3.3, black) following the addition of **corticosterone** (2.3–4.6 mM; from brown to pink) in CH_2_Cl_2_ at 25 °C, measured in a 1 mm cell; the excitation wavelength at which comparable absorbances were obtained was selected. (c) Normalized fluorescence spectra of (b). (d) Non-linear least-squares fitting, assuming the 1:1 stoichiometry with **SC**•(**sumanene**)_n_ and **corticosterone** monitored at 431 nm, to determine the binding constant at 25 °C.


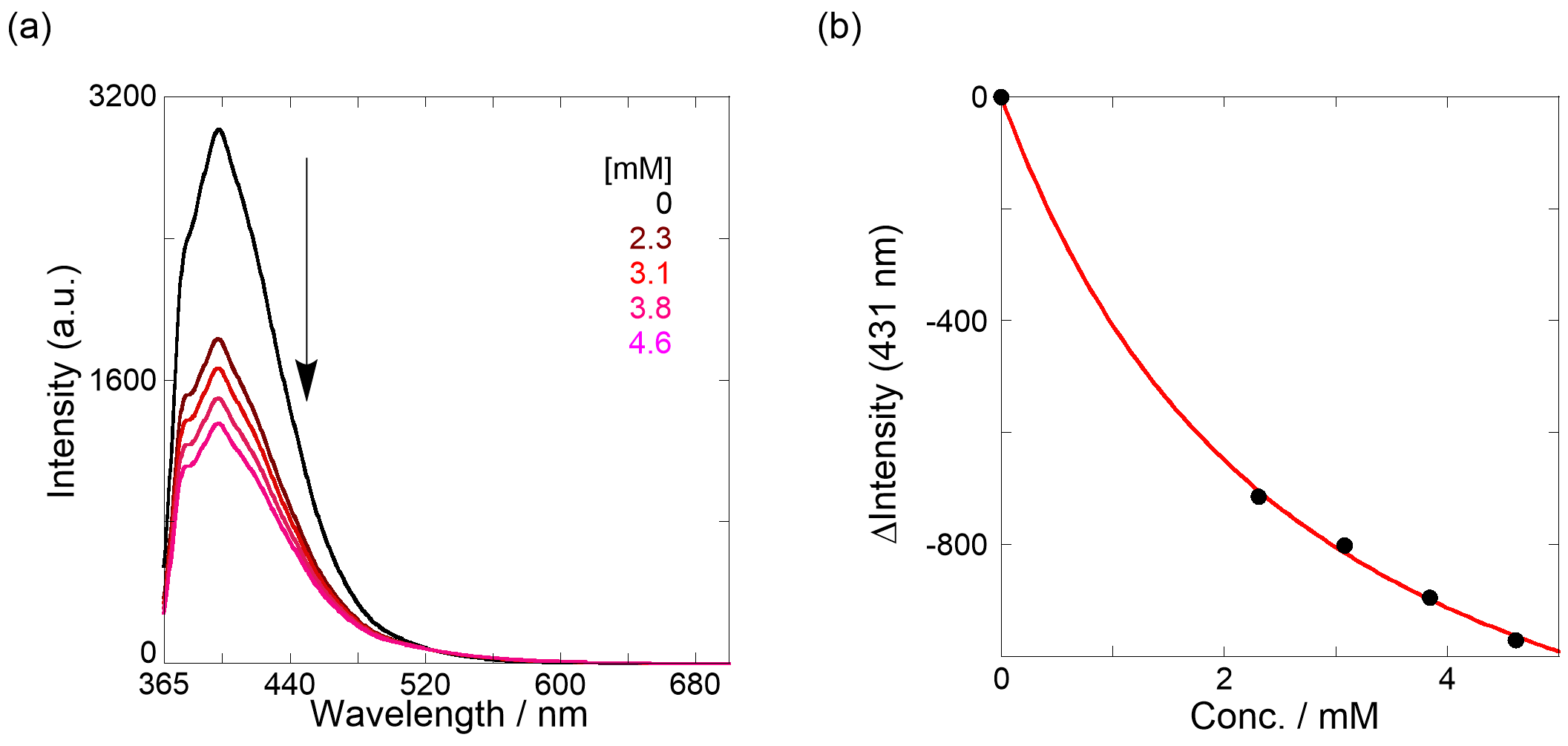


**Figure S51-2.** (a) Fluorescence (λ_ex_: 355 nm) spectra of **SC** (434 μM) with **sumanene** (9.28 mM, DP: 3.3, black) following the addition of **corticosterone** (2.3–4.6 mM; from brown to pink) in CH_2_Cl_2_ at 25 °C in a 1 mm cell. (b) Non-linear least-squares fitting, assuming the 1:1 stoichiometry with **SC**•(**sumanene**)_n_ and **corticosterone** monitored at 431 nm, to determine the binding constant as 430 ± 50 M^-1^ at 25 °C.


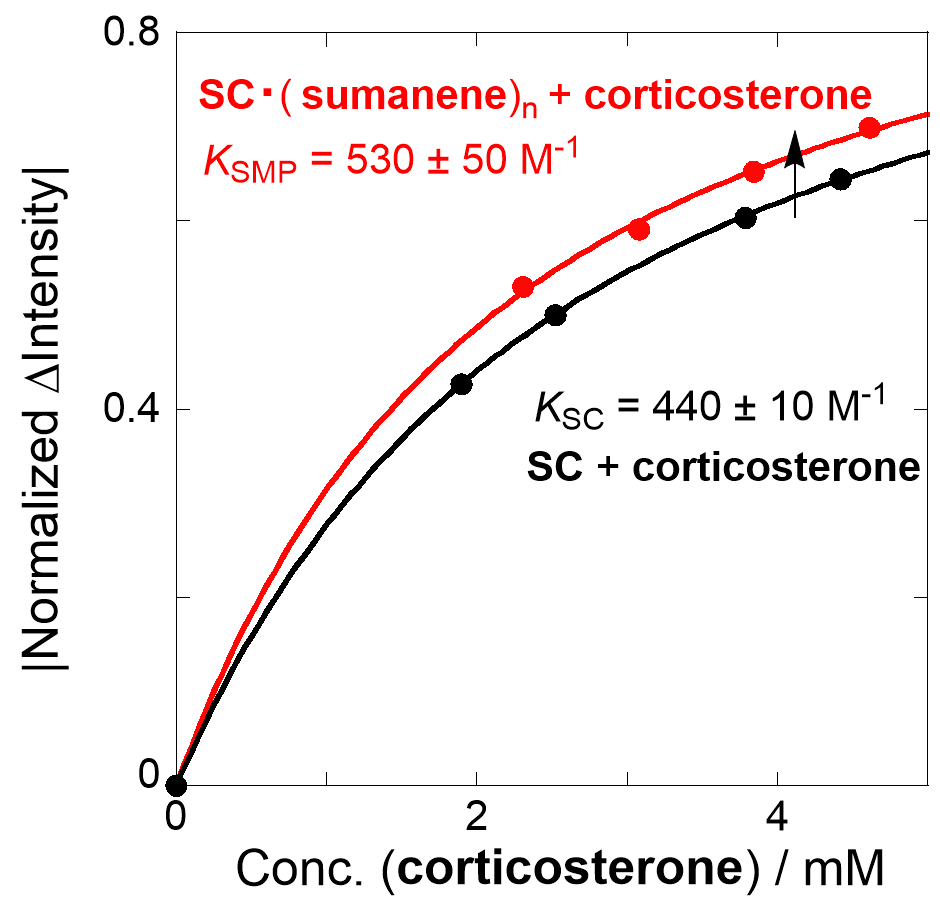


**Figure S52.** Normalized binding isotherms of **SC**•(**sumanene**)_n_ (red) and **SC** (black) following the addition of **corticosterone** at 25 °C.


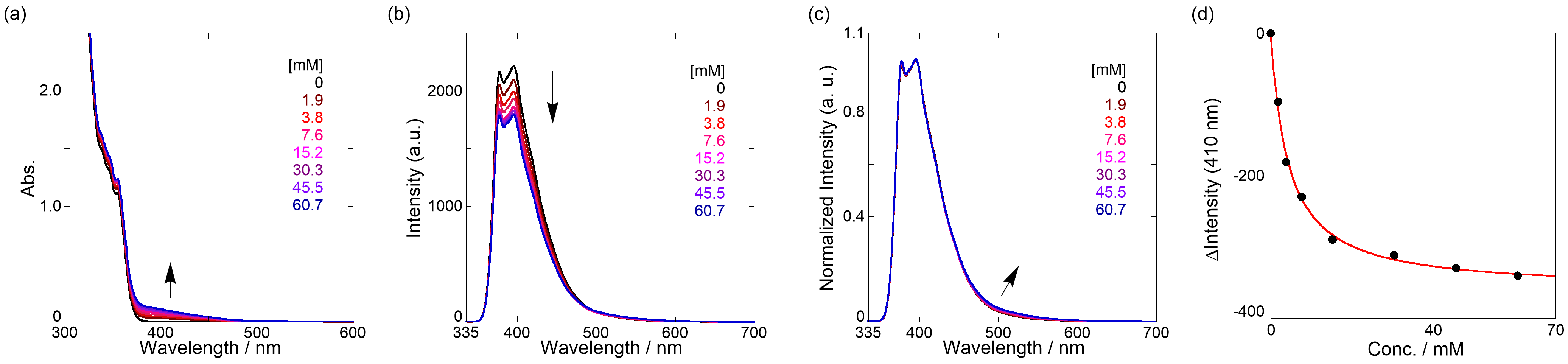


**Figure S53.** (a) UV/vis absorption and (b) fluorescence (λ_ex_: 325 nm) spectra of **SC** (437 μM) with **sumanene** (9.09 mM, DP: 3.3, black) following the addition of **allylestrenol** (1.9–60.7 mM; from brown to navy) in CH_2_Cl_2_ at 25 °C, measured in a 1 mm cell; the excitation wavelength at which comparable absorbances were obtained was selected. (c) Normalized fluorescence spectra of (b). (d) Non-linear least-squares fitting, assuming the 1:1 stoichiometry with **SC**•(**sumanene**)_n_ and **allylestrenol** monitored at 410 nm, to determine the binding constant at 25 °C.


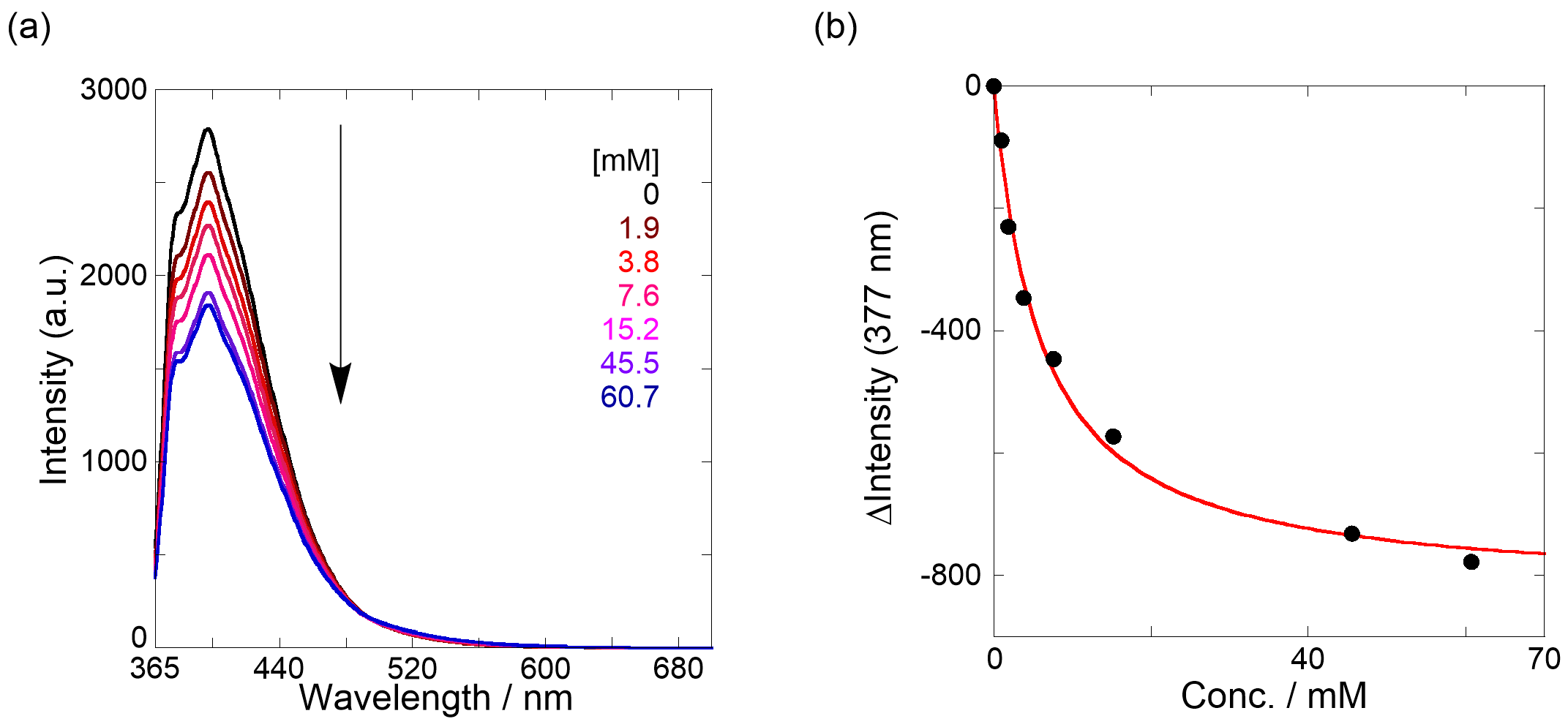


**Figure S53-2.** (a) Fluorescence (λ_ex_: 355 nm) spectra of **SC** (437 μM) with **sumanene** (9.09 mM, DP: 3.3, black) following the addition of **allylestrenol** (1.9–60.7 mM; from brown to navy) in CH_2_Cl_2_ at 25 °C in a 1 mm cell. (b) Non-linear least-squares fitting, assuming the 1:1 stoichiometry with **SC**•(**sumanene**)_n_ and **allylestrenol** monitored at 377 nm, to determine the binding constant as 180 ± 18 M^-1^ at 25 °C.

**Natural Population Analysis of SC**

| **Table S7.** Natural population analysis of **SC** | | | | |
| --- | --- | --- | --- | --- |
|  | **SC** | **SC**•**sumanene** | **SC**•(**sumanene**)_2_ | **SC**•(**sumanene**)_4_ |
| charge valence | 0 | -0.02 | -0.02 | -0.02 |

**Summary of Structure Optimization**

All computational studies were carried out by the Gaussian 16^[[9]](#footnote-10)^.

**SC**

| Symbol | X | Y | Z |
| --- | --- | --- | --- |
| C | 0.1369636 | -1.4079553 | -4.3521369 |
| C | 1.2938612 | -0.5567403 | -4.3516180 |
| C | 1.1411839 | 0.8109454 | -4.3571830 |
| C | -0.1744130 | 1.3873164 | -4.3539973 |
| C | -1.2825914 | 0.5712859 | -4.3548876 |
| C | -1.1238167 | -0.8562504 | -4.3492026 |
| C | -2.4548963 | 0.9326270 | -3.7003321 |
| C | -2.1875197 | -1.4652139 | -3.6907563 |
| C | 0.4119725 | -2.6023417 | -3.6955711 |
| C | 2.3550598 | -1.1721124 | -3.6950373 |
| C | 2.0400474 | 1.6476982 | -3.7050841 |
| C | -0.1702253 | 2.6152344 | -3.6997085 |
| C | -1.9648070 | -2.7220163 | -3.1427828 |
| C | -0.6598894 | -3.2884055 | -3.1404720 |
| C | 3.3339007 | -0.3496679 | -3.1520365 |
| C | 3.1718871 | 1.0636224 | -3.1523949 |
| C | -2.5133717 | 2.2058434 | -3.1496271 |
| C | -1.3703379 | 3.0526766 | -3.1539351 |
| C | -3.2468708 | -0.3633630 | -3.4319921 |
| C | 1.9309312 | -2.6397160 | -3.4308484 |
| C | 1.3135357 | 2.9822280 | -3.4407278 |
| H | -4.1168340 | -0.4818367 | -4.0841451 |
| H | 1.6464743 | 3.7932255 | -4.0946383 |
| H | 2.4668913 | -3.3360142 | -4.0820712 |
| H | 4.1726423 | -0.7734935 | -2.6120514 |
| H | 3.8858905 | 1.6606723 | -2.5960115 |
| H | -1.4209490 | 3.9919641 | -2.6154546 |
| H | -3.3858872 | 2.5267128 | -2.5914253 |
| H | -0.5000584 | -4.2032923 | -2.5807866 |
| H | -2.7515502 | -3.2345579 | -2.6013876 |
| O | -3.6881652 | -0.3329455 | -2.0754763 |
| O | 1.5593295 | 3.3524203 | -2.0848464 |
| O | 2.1282111 | -3.0326213 | -2.0734208 |
| C | 1.3001155 | 4.6340506 | -1.7386203 |
| C | 3.3669181 | -3.4501967 | -1.7256998 |
| C | -4.6694374 | -1.1961236 | -1.7266315 |
| O | -5.1791031 | -1.9761140 | -2.4862526 |
| O | 0.8787281 | 5.4633754 | -2.5000965 |
| O | 4.2953781 | -3.5057990 | -2.4872908 |
| C | -4.9990122 | -1.0448258 | -0.3102154 |
| C | -5.9406140 | -1.7266654 | 0.4167352 |
| N | -4.3472514 | -0.1457916 | 0.5021292 |
| C | -5.8712419 | -1.2280055 | 1.7493777 |
| H | -6.5941739 | -2.4886195 | 0.0227908 |
| C | -4.8628533 | -0.2421424 | 1.7662453 |
| H | -3.6056895 | 0.4570614 | 0.1911349 |
| C | 3.4037582 | -3.8052859 | -0.3078296 |
| C | 4.4665344 | -4.2772664 | 0.4186656 |
| N | 2.3013776 | -3.6860555 | 0.5067858 |
| C | 4.0032481 | -4.4600852 | 1.7533284 |
| H | 5.4521127 | -4.4646298 | 0.0231423 |
| C | 2.6455677 | -4.0788145 | 1.7718543 |
| H | 1.4079122 | -3.3461040 | 0.1966043 |
| C | 1.5945475 | 4.8469160 | -0.3223893 |
| C | 1.4724674 | 6.0044344 | 0.4023876 |
| N | 2.0480380 | 3.8351139 | 0.4922106 |
| C | 1.8685452 | 5.6979978 | 1.7359980 |
| H | 1.1383918 | 6.9502008 | 0.0063930 |
| C | 2.2201809 | 4.3323370 | 1.7556754 |
| H | 2.2009467 | 2.8911403 | 0.1830086 |
| C | 2.6569613 | 3.7049882 | 2.9299799 |
| C | 1.9547270 | 6.4632548 | 2.9170781 |
| C | -4.5358656 | 0.4515908 | 2.9389113 |
| C | -6.5764500 | -1.5327291 | 2.9316484 |
| C | 4.6225731 | -4.9132392 | 2.9361334 |
| C | 1.8842905 | -4.1366303 | 2.9467821 |
| H | -3.7615846 | 1.2098412 | 2.9514742 |
| H | -7.3502659 | -2.2884479 | 2.9145915 |
| C | -5.2325696 | 0.1399150 | 4.0788080 |
| C | -6.2499233 | -0.8467923 | 4.0821731 |
| H | -5.0239000 | 0.6437325 | 5.0145523 |
| H | 5.6637729 | -5.2060562 | 2.9177445 |
| H | 0.8406625 | -3.8446447 | 2.9606630 |
| C | 3.8682529 | -4.9675959 | 4.0888959 |
| C | 2.5052651 | -4.5791698 | 4.0871834 |
| H | 1.9669739 | -4.6456913 | 5.0246327 |
| H | 1.6857952 | 7.5108769 | 2.8978862 |
| H | 2.9280430 | 2.6557503 | 2.9446811 |
| C | 2.3850647 | 5.8401580 | 4.0692090 |
| C | 2.7328632 | 4.4662524 | 4.0686919 |
| H | 3.0644240 | 4.0357845 | 5.0055767 |
| O | -6.8411303 | -1.0331136 | 5.2915606 |
| O | 2.5174398 | 6.4478339 | 5.2775252 |
| O | 4.3280337 | -5.3810416 | 5.2990795 |
| C | 2.1953109 | 7.8162145 | 5.3622087 |
| C | -7.8664352 | -1.9945924 | 5.3793763 |
| C | 5.6732877 | -5.7887690 | 5.3854140 |
| H | 6.3579677 | -4.9721642 | 5.1263090 |
| H | 5.8362396 | -6.0776652 | 6.4222396 |
| H | 5.8749425 | -6.6484531 | 4.7350851 |
| H | -7.5001642 | -2.9967601 | 5.1258610 |
| H | -8.2010537 | -1.9867830 | 6.4152286 |
| H | -8.7100640 | -1.7429320 | 4.7254625 |
| H | 1.1439139 | 7.9978833 | 5.1085922 |
| H | 2.3692514 | 8.1048415 | 6.3973223 |
| H | 2.8339191 | 8.4203804 | 4.7066000 |

Total energy: -2804.299525 Hartree

**SC** ⸦ trimesate (C_6_H_3_(COO^-^)_3_)

| Symbol | X | Y | Z |
| --- | --- | --- | --- |
| C | 0.0345610 | -1.4084150 | -4.1220000 |
| C | 1.2535330 | -0.6427930 | -4.1287370 |
| C | 1.2024220 | 0.7329580 | -4.1231450 |
| C | -0.0701080 | 1.4058620 | -4.1286380 |
| C | -1.2359660 | 0.6737250 | -4.1219870 |
| C | -1.1824180 | -0.7648020 | -4.1276970 |
| C | -2.3664090 | 1.1144280 | -3.4264420 |
| C | -2.2798210 | -1.2871680 | -3.4394230 |
| C | 0.2187780 | -2.6078840 | -3.4268030 |
| C | 2.2553080 | -1.3320630 | -3.4415680 |
| C | 2.1498750 | 1.4921860 | -3.4288860 |
| C | 0.0266560 | 2.6178560 | -3.4413070 |
| C | -2.1368360 | -2.5119310 | -2.8128370 |
| C | -0.8779820 | -3.1804190 | -2.8095140 |
| C | 3.2453690 | -0.5957770 | -2.8163710 |
| C | 3.1948880 | 0.8286730 | -2.8129930 |
| C | -2.3135830 | 2.3509430 | -2.8100950 |
| C | -1.1052470 | 3.1068900 | -2.8145310 |
| C | -3.2619000 | -0.1248290 | -3.2073920 |
| C | 1.7399550 | -2.7637900 | -3.2089110 |
| C | 1.5244460 | 2.8875000 | -3.2102000 |
| H | -4.0873400 | -0.1277700 | -3.9291200 |
| H | 1.9340650 | 3.6036180 | -3.9324080 |
| H | 2.1549000 | -3.4771310 | -3.9307960 |
| H | 3.9428130 | -1.0721950 | -2.1366400 |
| H | 3.8650200 | 1.3490460 | -2.1398190 |
| H | -1.0407340 | 3.9489000 | -2.1345900 |
| H | -3.0986170 | 2.6709640 | -2.1360620 |
| H | -0.7624420 | -4.0204000 | -2.1356540 |
| H | -2.8975770 | -2.8767350 | -2.1319130 |
| O | -3.8125510 | -0.2407620 | -1.9072850 |
| O | 1.6993580 | 3.4227220 | -1.9101860 |
| O | 2.1164310 | -3.1822750 | -1.9087770 |
| C | 2.7882660 | 4.1578240 | -1.6659900 |
| C | 2.2107940 | -4.4928620 | -1.6652180 |
| C | -4.9937890 | 0.3348820 | -1.6643330 |
| O | -5.5893730 | 1.0319870 | -2.4528570 |
| O | 3.6903470 | 4.3259320 | -2.4536770 |
| O | 1.9068910 | -5.3583290 | -2.4533020 |
| C | -5.4975910 | -0.0488750 | -0.3386100 |
| C | -6.6884890 | 0.3521980 | 0.2360060 |
| N | -4.8427880 | -0.9310320 | 0.4637600 |
| C | -6.7723800 | -0.3247940 | 1.4794300 |
| H | -7.3829200 | 1.0528160 | -0.2006850 |
| C | -5.5987490 | -1.1140680 | 1.5751950 |
| H | -3.8996730 | -1.4418430 | 0.3097610 |
| C | 2.7941720 | -4.7352350 | -0.3387490 |
| C | 3.0430340 | -5.9660650 | 0.2379240 |
| N | 3.2283640 | -3.7254830 | 0.4629060 |
| C | 3.6690810 | -5.6977160 | 1.4819290 |
| H | 2.7852520 | -6.9187320 | -0.1977270 |
| C | 3.7639460 | -4.2864310 | 1.5758380 |
| H | 3.1983920 | -2.6533840 | 0.3078460 |
| C | 2.7066810 | 4.7845090 | -0.3395680 |
| C | 3.6492130 | 5.6139550 | 0.2375120 |
| N | 1.6146720 | 4.6573430 | 0.4618350 |
| C | 3.1042130 | 6.0224610 | 1.4815810 |
| H | 4.6036590 | 5.8655810 | -0.1978220 |
| C | 1.8336520 | 5.4008920 | 1.5750990 |
| C | 0.9971300 | 5.5970320 | 2.6826390 |
| C | 3.5475320 | 6.8567070 | 2.5326440 |
| C | -5.3529980 | -1.9374080 | 2.6827210 |
| C | -7.7189980 | -0.3591200 | 2.5282120 |
| C | 4.1712830 | -6.4984150 | 2.5325840 |
| C | 4.3514610 | -3.6596470 | 2.6834520 |
| H | -4.4460580 | -2.5300700 | 2.7149090 |
| H | -8.6134310 | 0.2490280 | 2.4644970 |
| C | -6.2896800 | -1.9582830 | 3.6884890 |
| C | -7.4637780 | -1.1715020 | 3.6116650 |
| H | -6.1509110 | -2.5719170 | 4.5714060 |
| H | 4.0929630 | -7.5771910 | 2.4695720 |
| H | 4.4097550 | -2.5777490 | 2.7142370 |
| C | 4.7446060 | -5.8689690 | 3.6161160 |
| C | 4.8370060 | -4.4585200 | 3.6911000 |
| H | 5.2970390 | -4.0298480 | 4.5742490 |
| H | 4.5216060 | 7.3269890 | 2.4702150 |
| H | 0.0303700 | 5.1078540 | 2.7130230 |
| C | 2.7157340 | 7.0393530 | 3.6160650 |
| C | 1.4471390 | 6.4159880 | 3.6906460 |
| H | 0.8459890 | 6.6005790 | 4.5737660 |
| O | -8.3050180 | -1.2978450 | 4.7018610 |
| O | 3.0259300 | 7.8286930 | 4.7083030 |
| O | 5.2739260 | -6.5318500 | 4.7081630 |
| C | 4.2883270 | 8.4255970 | 4.7213270 |
| C | -9.4531000 | -0.5028810 | 4.7119400 |
| C | 5.1619450 | -7.9237370 | 4.7204420 |
| H | 5.7024910 | -8.3888660 | 3.8831080 |
| H | 5.6042670 | -8.2629680 | 5.6594580 |
| H | 4.1139250 | -8.2519020 | 4.6784180 |
| H | -10.1248210 | -0.7371850 | 3.8731620 |
| H | -9.9703500 | -0.7153810 | 5.6498910 |
| H | -9.2111690 | 0.5683540 | 4.6703120 |
| H | 4.4226990 | 9.1253870 | 3.8835350 |
| H | 4.3610130 | 8.9788730 | 5.6599830 |
| H | 5.0953860 | 7.6807550 | 4.6806280 |
| C | 0.7341260 | 1.1729920 | 0.2930370 |
| C | -0.6561320 | 1.2423990 | 0.2925430 |
| C | -1.3843040 | 0.0512540 | 0.2942210 |
| C | -0.7491370 | -1.1874260 | 0.2920530 |
| C | 0.6465350 | -1.2225100 | 0.2923350 |
| C | 1.4016540 | -0.0531460 | 0.2908940 |
| H | 1.3389560 | 2.0681490 | 0.3006800 |
| H | -2.4619540 | 0.1271100 | 0.3027880 |
| H | 1.1194210 | -2.1938580 | 0.2990240 |
| C | 2.9292460 | -0.0613070 | 0.3116710 |
| C | -1.5204350 | -2.5060480 | 0.3140440 |
| C | -1.4119950 | 2.5699080 | 0.3143030 |
| O | 3.5169980 | -1.1937780 | 0.3659940 |
| O | -2.6476730 | 2.5338690 | 0.2597640 |
| O | -2.7950310 | -2.4480910 | 0.3684520 |
| O | -0.8709220 | -3.5578320 | 0.2592520 |
| O | 3.5148100 | 1.0273160 | 0.2553620 |
| O | -0.7239430 | 3.6444360 | 0.3676620 |
| H | 0.7003780 | 4.0969760 | 0.3071490 |

Total energy: -3600.5013 Hartree

**SC**•**sumanene**

| Symbol | X | Y | Z |
| --- | --- | --- | --- |
| C | -2.4960560 | -1.1140690 | 0.8496980 |
| C | -2.4945720 | -1.2787740 | -0.5719040 |
| C | -2.4965220 | -0.1767220 | -1.3898150 |
| C | -2.4935250 | 1.1367600 | -0.8216840 |
| C | -2.4950420 | 1.2940580 | 0.5416660 |
| C | -2.4930930 | 0.1452900 | 1.3951300 |
| C | -1.8511260 | 2.3618600 | 1.1566990 |
| C | -1.8500480 | 0.4245710 | 2.5965050 |
| C | -1.8530210 | -2.1812580 | 1.4667530 |
| C | -1.8528080 | -2.4594930 | -0.9308820 |
| C | -1.8537710 | -0.1782180 | -2.6226850 |
| C | -1.8510280 | 2.0373440 | -1.6648980 |
| C | -1.3176100 | -0.6492080 | 3.2962260 |
| C | -1.3142220 | -1.9564040 | 2.7251900 |
| C | -1.3207380 | -2.5291530 | -2.2108160 |
| C | -1.3163190 | -1.3810300 | -3.0573370 |
| C | -1.3129180 | 3.3391550 | 0.3322260 |
| C | -1.3177350 | 3.1800800 | -1.0853440 |
| C | -1.6084640 | 1.9555100 | 2.6242290 |
| C | -1.6118660 | -3.2492550 | 0.3810060 |
| C | -1.6109030 | 1.2957550 | -3.0047740 |
| H | -2.2955370 | 2.4483450 | 3.3173210 |
| H | -2.2983940 | 1.6497960 | -3.7776650 |
| H | -2.2999860 | -4.0950410 | 0.4614560 |
| H | -0.7923150 | -3.4156020 | -2.5423840 |
| H | -0.7685800 | -1.4379200 | -3.9915450 |
| H | -0.7886510 | 3.9099330 | -1.6873220 |
| H | -0.7643340 | 4.1761700 | 0.7499090 |
| H | -0.7662130 | -2.7371520 | 3.2410550 |
| H | -0.7880570 | -0.4932890 | 4.2291000 |
| O | -0.2705560 | 2.3020880 | 2.9753210 |
| O | -0.2732890 | 1.4261670 | -3.4814130 |
| O | -0.2745730 | -3.7285110 | 0.5052580 |
| C | 0.0179830 | 2.5483590 | -4.1773480 |
| C | 0.0148330 | -4.8928350 | -0.1185540 |
| C | 0.0209580 | 2.3447540 | 4.2950690 |
| O | -0.7740220 | 2.1082650 | 5.1660120 |
| O | -0.7772470 | 3.4208280 | -4.4072880 |
| O | -0.7814050 | -5.5268650 | -0.7592830 |
| C | 1.4232330 | 2.7052650 | 4.4962700 |
| C | 2.0944830 | 2.8638600 | 5.6813590 |
| N | 2.2797510 | 2.9327960 | 3.4439260 |
| C | 3.4370230 | 3.2064550 | 5.3505950 |
| H | 1.6579940 | 2.7459960 | 6.6603820 |
| C | 3.5169140 | 3.2385500 | 3.9428670 |
| H | 2.0136420 | 2.8602240 | 2.4775070 |
| C | 1.4164190 | -5.2503850 | 0.0924500 |
| C | 2.0850090 | -6.3570400 | -0.3640910 |
| N | 2.2749740 | -4.4555590 | 0.8163650 |
| C | 3.4279170 | -6.2455050 | 0.0977970 |
| H | 1.6465660 | -7.1443210 | -0.9564260 |
| C | 3.5107710 | -5.0434430 | 0.8307670 |
| H | 2.0111810 | -3.5821150 | 1.2377150 |
| C | 1.4202780 | 2.5432280 | -4.5901730 |
| C | 2.0911310 | 3.4913010 | -5.3190510 |
| N | 2.2773530 | 1.5182840 | -4.2618020 |
| C | 3.4339770 | 3.0345070 | -5.4504310 |
| H | 1.6542020 | 4.3981910 | -5.7057700 |
| C | 3.5144720 | 1.7987620 | -4.7755000 |
| H | 2.0117160 | 0.7170130 | -3.7164260 |
| C | 4.7129740 | 1.0756840 | -4.7115750 |
| C | 4.5768920 | 3.5668170 | -6.0815930 |
| C | 4.7149080 | 3.5447490 | 3.2838060 |
| C | 4.5801870 | 3.4882500 | 6.1263330 |
| C | 4.5690300 | -7.0608350 | -0.0472250 |
| C | 4.7099150 | -4.6293380 | 1.4256560 |
| H | 4.7759030 | 3.5701880 | 2.2020380 |
| H | 4.5147000 | 3.4641240 | 7.2058290 |
| C | 5.8160070 | 3.8148480 | 4.0561570 |
| C | 5.7558570 | 3.7887330 | 5.4718960 |
| H | 6.7687050 | 4.0585720 | 3.6023490 |
| H | 4.5012100 | -7.9828400 | -0.6089140 |
| H | 4.7733260 | -3.7060000 | 1.9895850 |
| C | 5.7458210 | -6.6478330 | 0.5404420 |
| C | 5.8090200 | -5.4357750 | 1.2722350 |
| H | 6.7624580 | -5.1673900 | 1.7103040 |
| H | 4.5108340 | 4.5142230 | -6.5995040 |
| H | 4.7745630 | 0.1256830 | -4.1936010 |
| C | 5.7530460 | 2.8505690 | -6.0150470 |
| C | 5.8138500 | 1.6108660 | -5.3309180 |
| H | 6.7668990 | 1.0966260 | -5.3153110 |
| O | 6.9332360 | 4.0799460 | 6.0848270 |
| O | 6.9302760 | 3.2370940 | -6.5731230 |
| O | 6.9215150 | -7.3270920 | 0.4851950 |
| C | 6.9536730 | 4.4575720 | -7.2756050 |
| C | 6.9573890 | 4.0794160 | 7.4930200 |
| C | 6.9423760 | -8.5456270 | -0.2207250 |
| H | 6.6903960 | -8.4015260 | -1.2782940 |
| H | 7.9604430 | -8.9231380 | -0.1431340 |
| H | 6.2519900 | -9.2758650 | 0.2182820 |
| H | 6.7050280 | 3.0922870 | 7.8987210 |
| H | 7.9764830 | 4.3342870 | 7.7784710 |
| H | 6.2690100 | 4.8261470 | 7.9067060 |
| H | 6.7008730 | 5.3017050 | -6.6227320 |
| H | 7.9726480 | 4.5782760 | -7.6390780 |
| H | 6.2651680 | 4.4428960 | -8.1290440 |
| C | -6.1345170 | -0.1373190 | -1.3950010 |
| C | -6.1327850 | 0.1731320 | 1.3947170 |
| C | -6.1333420 | 1.1246900 | -0.8423700 |
| C | -6.1345470 | -1.2884570 | -0.5479050 |
| C | -6.1339990 | -1.1360110 | 0.8213440 |
| C | -6.1328820 | 1.2827360 | 0.5781000 |
| C | -5.5365960 | 2.2115840 | -1.4634190 |
| C | -5.0733640 | 1.9900820 | -2.7627690 |
| H | -4.5786010 | 2.7818860 | -3.3169840 |
| C | -5.5395680 | -0.4186230 | -2.6156200 |
| C | -5.0764040 | 0.6870300 | -3.3333980 |
| H | -4.5862460 | 0.5575130 | -4.2934790 |
| C | -5.5364620 | 2.4797710 | 0.9446900 |
| C | -5.0732160 | 2.5481110 | 2.2611150 |
| H | -4.5821740 | 3.4438490 | 2.6289530 |
| C | -5.0715700 | 1.4024960 | 3.1043750 |
| H | -4.5767490 | 1.4861220 | 4.0672140 |
| C | -5.5359190 | 0.1668760 | 2.6464630 |
| C | -5.5383270 | -2.0526840 | 1.6745200 |
| C | -5.0765560 | -3.2273880 | 1.0753190 |
| H | -4.5860210 | -3.9943030 | 1.6669210 |
| C | -5.0754970 | -3.3848590 | -0.3384540 |
| H | -4.5816220 | -4.2609760 | -0.7476130 |
| C | -5.5392250 | -2.3701060 | -1.1794000 |
| C | -5.2602560 | -1.3138700 | 3.0073010 |
| H | -5.9008970 | -1.6689700 | 3.8209790 |
| H | -4.2220660 | -1.4518790 | 3.3221230 |
| C | -5.2596230 | 3.2644500 | -0.3618470 |
| H | -5.9003540 | 4.1466740 | -0.4605320 |
| H | -4.2214950 | 3.6061470 | -0.4008620 |
| C | -5.2638560 | -1.9426560 | -2.6423830 |
| H | -5.9060680 | -2.4686570 | -3.3561820 |
| H | -4.2263050 | -2.1481790 | -2.9203040 |

Total energy: -3611.675561 Hartree

**SC**•(**sumanene**)_2_

| Symbol | X | Y | Z |
| --- | --- | --- | --- |
| C | -0.7100186 | -0.5909655 | -1.2722120 |
| C | -0.7079236 | 0.8328068 | -1.1287985 |
| C | -0.7094189 | 1.3958325 | 0.1227455 |
| C | -0.7065773 | 0.5597355 | 1.2840361 |
| C | -0.7085991 | -0.8056318 | 1.1458745 |
| C | -0.7071764 | -1.3933150 | -0.1588470 |
| C | -0.0649475 | -1.6353101 | 2.0572350 |
| C | -0.0651850 | -2.6271850 | -0.1433708 |
| C | -0.0677372 | -0.9656013 | -2.4470709 |
| C | -0.0665538 | 1.4360261 | -2.2056555 |
| C | -0.0663584 | 2.6003347 | 0.3851333 |
| C | -0.0641271 | 1.1905209 | 2.3443439 |
| C | 0.4666307 | -3.0809951 | -1.3421893 |
| C | 0.4702765 | -2.2432627 | -2.4969414 |
| C | 0.4661589 | 2.7008176 | -1.9997183 |
| C | 0.4711493 | 3.2820242 | -0.6968638 |
| C | 0.4736742 | -1.0392840 | 3.1881733 |
| C | 0.4691544 | 0.3796536 | 3.3363157 |
| C | 0.1750410 | -2.9824298 | 1.3462495 |
| C | 0.1726154 | 0.3235240 | -3.2582879 |
| C | 0.1746471 | 2.6584235 | 1.9070257 |
| H | -0.5151138 | -3.7624855 | 1.6787900 |
| H | -0.5157349 | 3.3358442 | 2.4168262 |
| H | -0.5175367 | 0.4260747 | -4.1000493 |
| H | 0.9948383 | 3.2143629 | -2.7945443 |
| H | 1.0193228 | 4.2065361 | -0.5527194 |
| H | 0.9985330 | 0.8111676 | 4.1780707 |
| H | 1.0222962 | -1.6265343 | 3.9162822 |
| H | 1.0179025 | -2.5803931 | -3.3701207 |
| H | 0.9956390 | -4.0259035 | -1.3900501 |
| O | 1.5109029 | -3.4047966 | 1.6103499 |
| O | 1.5103350 | 3.0992791 | 2.1398696 |
| O | 1.5085961 | 0.3051936 | -3.7558105 |
| C | 1.7958922 | 3.5412249 | 3.3857611 |
| C | 1.7948231 | 1.1625355 | -4.7618739 |
| C | 1.7955658 | -4.7052222 | 1.3716409 |
| O | 0.9958146 | -5.5015187 | 0.9557769 |
| O | 0.9969279 | 3.5772274 | 4.2840892 |
| O | 0.9960212 | 1.9213946 | -5.2442454 |
| C | 3.1960675 | -4.9863951 | 1.6810579 |
| C | 3.8601641 | -6.1821857 | 1.5844618 |
| N | 4.0583846 | -4.0115757 | 2.1272183 |
| C | 5.2040557 | -5.9398409 | 1.9898470 |
| H | 3.4181842 | -7.1112322 | 1.2613068 |
| C | 5.2921095 | -4.5715904 | 2.3202696 |
| H | 3.7982651 | -3.0501286 | 2.2612658 |
| C | 3.1959597 | 1.0349842 | -5.1578366 |
| C | 3.8621737 | 1.7169280 | -6.1434299 |
| N | 4.0568902 | 0.1607467 | -4.5354124 |
| C | 5.2060208 | 1.2445680 | -6.1336651 |
| H | 3.4216313 | 2.4617420 | -6.7869177 |
| C | 5.2918461 | 0.2737343 | -5.1142708 |
| H | 3.7951083 | -0.4363790 | -3.7706078 |
| C | 3.1962087 | 3.9505929 | 3.4739226 |
| C | 3.8609837 | 4.4634870 | 4.5580445 |
| N | 4.0576223 | 3.8518789 | 2.4056740 |
| C | 5.2044169 | 4.6946634 | 4.1446782 |
| H | 3.4198058 | 4.6462818 | 5.5249180 |
| C | 5.2914586 | 4.2990673 | 2.7937557 |
| H | 3.7968605 | 3.4887040 | 1.5056124 |
| C | 6.4921666 | 4.3958012 | 2.0779850 |
| C | 6.3428348 | 5.2025049 | 4.8035549 |
| C | 6.4935160 | -3.9999547 | 2.7598875 |
| C | 6.3421144 | -6.7647941 | 2.1008152 |
| C | 6.3457703 | 1.5613683 | -6.9008993 |
| C | 6.4926207 | -0.3930034 | -4.8367823 |
| H | 6.5609118 | -2.9486786 | 3.0145655 |
| H | 6.2703175 | -7.8140993 | 1.8478666 |
| C | 7.5895130 | -4.8188328 | 2.8602147 |
| C | 7.5210954 | -6.1963802 | 2.5340074 |
| H | 8.5444667 | -4.4330353 | 3.1951892 |
| H | 6.2756485 | 2.3055223 | -7.6828754 |
| H | 6.5582818 | -1.1396475 | -4.0539695 |
| C | 7.5241479 | 0.9018799 | -6.6229638 |
| C | 7.5902904 | -0.0700186 | -5.5934944 |
| H | 8.5448703 | -0.5531195 | -5.4250413 |
| H | 6.2717319 | 5.5061408 | 5.8393690 |
| H | 6.5587907 | 4.0925935 | 1.0396159 |
| C | 7.5211707 | 5.2951211 | 4.0938367 |
| C | 7.5885305 | 4.8913814 | 2.7369231 |
| H | 8.5430082 | 4.9898487 | 2.2346680 |
| O | 8.6942251 | -6.8643284 | 2.6890441 |
| O | 8.6946619 | 5.7626755 | 4.5945947 |
| O | 8.6987321 | 1.1020743 | -7.2761809 |
| C | 8.7117560 | 6.1890408 | 5.9368565 |
| C | 8.7100340 | -8.2405322 | 2.3898036 |
| C | 8.7167649 | 2.0501711 | -8.3175795 |
| H | 8.4634729 | 3.0527352 | -7.9523342 |
| H | 9.7343384 | 2.0560226 | -8.7041956 |
| H | 8.0257730 | 1.7746023 | -9.1233005 |
| H | 8.4576798 | -8.4259387 | 1.3387871 |
| H | 9.7267671 | -8.5799753 | 2.5795156 |
| H | 8.0173236 | -8.7987760 | 3.0309803 |
| H | 8.4601480 | 5.3701941 | 6.6216016 |
| H | 9.7286676 | 6.5227301 | 6.1355969 |
| H | 8.0191640 | 7.0230808 | 6.1020311 |
| C | -4.3294993 | 1.3905729 | 0.1610083 |
| C | -4.3297137 | -1.3946015 | -0.1330741 |
| C | -4.3288719 | 0.5818718 | 1.2730715 |
| C | -4.3300741 | 0.8113839 | -1.1416618 |
| C | -4.3303750 | -0.5560460 | -1.2859697 |
| C | -4.3291483 | -0.8358667 | 1.1233101 |
| C | -3.7385544 | 0.9563862 | 2.4709726 |
| C | -3.2856810 | 2.2752009 | 2.5363676 |
| H | -2.7965285 | 2.6505823 | 3.4300935 |
| C | -3.7401178 | 2.6459769 | 0.1481766 |
| C | -3.2879591 | 3.1135500 | 1.3834098 |
| H | -2.8033635 | 4.0815865 | 1.4662391 |
| C | -3.7393489 | -1.4527332 | 2.2165686 |
| C | -3.2880950 | -2.7565208 | 2.0036268 |
| H | -2.8033252 | -3.3125729 | 2.8002499 |
| C | -3.2871668 | -3.3359021 | 0.7011223 |
| H | -2.7987986 | -4.2979401 | 0.5790925 |
| C | -3.7403230 | -2.6196281 | -0.4080314 |
| C | -3.7416411 | -1.1947609 | -2.3672219 |
| C | -3.2903955 | -0.3587527 | -3.3901333 |
| H | -2.8064910 | -0.7709681 | -4.2703248 |
| C | -3.2883133 | 1.0589372 | -3.2405251 |
| H | -2.7999513 | 1.6453127 | -4.0129039 |
| C | -3.7404617 | 1.6616038 | -2.0653656 |
| C | -3.4660215 | -2.6547642 | -1.9313239 |
| H | -4.1117707 | -3.3699907 | -2.4504301 |
| H | -2.4289912 | -2.9357909 | -2.1352220 |
| C | -3.4629743 | -0.3451975 | 3.2626683 |
| H | -4.1074705 | -0.4374732 | 4.1424754 |
| H | -2.4255523 | -0.3808828 | 3.6067658 |
| C | -3.4648850 | 2.9982041 | -1.3342260 |
| H | -4.1099358 | 3.8059857 | -1.6938203 |
| H | -2.4277087 | 3.3144851 | -1.4760954 |
| C | -8.0298754 | 0.8392504 | -1.1240587 |
| C | -8.0291386 | -0.8132115 | 1.1459397 |
| C | -8.0293093 | 1.3985168 | 0.1352232 |
| C | -8.0300609 | -0.5826412 | -1.2748125 |
| C | -8.0296810 | -1.3935757 | -0.1608377 |
| C | -8.0288435 | 0.5569963 | 1.2912166 |
| C | -7.4258879 | 2.6171427 | 0.4094904 |
| C | -6.9538777 | 3.3252978 | -0.6971752 |
| H | -6.4477764 | 4.2770232 | -0.5688089 |
| C | -7.4269949 | 1.4525582 | -2.2125553 |
| C | -6.9544196 | 2.7482662 | -1.9964371 |
| H | -6.4488322 | 3.2909366 | -2.7890693 |
| C | -7.4249968 | 1.1926816 | 2.3662676 |
| C | -6.9519667 | 0.3573862 | 3.3799346 |
| H | -6.4451687 | 0.7722098 | 4.2456532 |
| C | -6.9522590 | -1.0563315 | 3.2300008 |
| H | -6.4456308 | -1.6436035 | 3.9895098 |
| C | -7.4255543 | -1.6603923 | 2.0637700 |
| C | -7.4264706 | -2.6427387 | -0.1481902 |
| C | -6.9542617 | -3.1031038 | -1.3786375 |
| H | -6.4479565 | -4.0604588 | -1.4525895 |
| C | -6.9548032 | -2.2664467 | -2.5280022 |
| H | -6.4490093 | -2.6308638 | -3.4166818 |
| C | -7.4273224 | -0.9542026 | -2.4677424 |
| C | -7.1444562 | -2.9949230 | 1.3321503 |
| H | -7.7792365 | -3.8103477 | 1.6944811 |
| H | -6.1032897 | -3.3006612 | 1.4673987 |
| C | -7.1430140 | 2.6507222 | 1.9306605 |
| H | -7.7765752 | 3.3726451 | 2.4565421 |
| H | -6.1013140 | 2.9199149 | 2.1263115 |
| C | -7.1456324 | 0.3464415 | -3.2577055 |
| H | -7.7804007 | 0.4406524 | -4.1450326 |
| H | -6.1044034 | 0.3816267 | -3.5900758 |

Total energy: -4419.050022 Hartree

**SC**•(**sumanene**)_3_

| Symbol | X | Y | Z |
| --- | --- | --- | --- |
| C | -1.0903990 | -0.8380800 | 1.1224290 |
| C | -1.0927820 | 0.5847820 | 1.2751870 |
| C | -1.0907890 | 1.3933680 | 0.1663650 |
| C | -1.0924190 | 0.8143000 | -1.1422010 |
| C | -1.0900440 | -0.5502740 | -1.2881020 |
| C | -1.0921000 | -1.3939570 | -0.1322860 |
| C | -1.7327510 | -1.1747370 | -2.3512790 |
| C | -1.7336990 | -2.5983130 | -0.4020580 |
| C | -1.7336240 | -1.4466670 | 2.1943490 |
| C | -1.7346510 | 0.9532670 | 2.4529380 |
| C | -1.7338820 | 2.6260770 | 0.1571480 |
| C | -1.7342220 | 1.6497130 | -2.0505130 |
| C | -2.2666650 | -3.2892160 | 0.6770240 |
| C | -2.2715730 | -2.7071720 | 1.9794840 |
| C | -2.2675250 | 2.2333270 | 2.5115290 |
| C | -2.2720480 | 3.0703090 | 1.3561370 |
| C | -2.2708120 | -0.3587500 | -3.3356750 |
| C | -2.2666560 | 1.0602720 | -3.1885320 |
| C | -1.9729590 | -2.6392850 | -1.9330890 |
| C | -1.9736300 | -0.3523550 | 3.2538190 |
| C | -1.9739390 | 2.9961280 | -1.3204980 |
| H | -1.2826370 | -3.3343100 | -2.4185460 |
| H | -1.2837560 | 3.7642290 | -1.6795480 |
| H | -1.2825420 | -0.4257950 | 4.0978980 |
| H | -2.7966000 | 2.5721190 | 3.3949030 |
| H | -2.8204380 | 4.0045600 | 1.4051480 |
| H | -2.7957610 | 1.6556090 | -3.9238330 |
| H | -2.8189910 | -0.7836210 | -4.1693200 |
| H | -2.8200170 | -3.2168380 | 2.7639790 |
| H | -2.7956320 | -4.2236420 | 0.5284980 |
| O | -3.3083250 | -2.9989710 | -2.2791080 |
| O | -3.3094580 | 3.4753620 | -1.4589740 |
| O | -3.3088520 | -0.4727330 | 3.7385500 |
| C | -3.5929260 | 4.1654760 | -2.5872110 |
| C | -3.5930220 | 0.1590840 | 4.9002840 |
| C | -3.5909090 | -4.3212840 | -2.3104490 |
| O | -2.7895530 | -5.1839710 | -2.0646320 |
| O | -2.7922120 | 4.3863830 | -3.4571460 |
| O | -2.7928350 | 0.8020020 | 5.5272470 |
| C | -4.9908250 | -4.5359290 | -2.6706310 |
| C | -5.6527860 | -5.7276170 | -2.8192660 |
| N | -5.8551960 | -3.4922460 | -2.9086370 |
| C | -6.9973570 | -5.4102480 | -3.1662120 |
| H | -5.2089570 | -6.7021980 | -2.6922930 |
| C | -7.0880740 | -4.0035710 | -3.2111350 |
| H | -5.5971680 | -2.5231150 | -2.8442110 |
| C | -4.9935840 | -0.0462920 | 5.2634430 |
| C | -5.6577240 | 0.4186290 | 6.3694230 |
| N | -5.8562710 | -0.7729910 | 4.4756500 |
| C | -7.0020360 | -0.0405980 | 6.2646690 |
| H | -5.2155490 | 1.0146890 | 7.1517840 |
| C | -7.0902940 | -0.7806640 | 5.0673380 |
| H | -5.5962210 | -1.2002930 | 3.6039940 |
| C | -4.9932750 | 4.5833200 | -2.5917850 |
| C | -5.6567110 | 5.3078650 | -3.5484600 |
| N | -5.8563870 | 4.2663120 | -1.5683290 |
| C | -7.0009930 | 5.4484850 | -3.0987830 |
| H | -5.2140260 | 5.6859110 | -4.4562200 |
| C | -7.0899560 | 4.7832070 | -1.8583730 |
| H | -5.5968760 | 3.7256500 | -0.7618840 |
| C | -8.2919390 | 4.7298440 | -1.1401480 |
| C | -8.1387900 | 6.0806390 | -3.6409450 |
| C | -8.2908980 | -3.3567400 | -3.5246940 |
| C | -8.1340100 | -6.1976040 | -3.4421850 |
| C | -8.1404190 | 0.1123160 | 7.0824580 |
| C | -8.2921580 | -1.3750940 | 4.6603450 |
| H | -8.3604450 | -2.2757690 | -3.5601760 |
| H | -8.0602220 | -7.2762930 | -3.4081770 |
| C | -9.3855120 | -4.1402040 | -3.7889310 |
| C | -9.3144020 | -5.5552190 | -3.7498980 |
| H | -10.3414540 | -3.6961450 | -4.0378940 |
| H | -8.0684380 | 0.6795250 | 8.0007520 |
| H | -8.3597070 | -1.9444720 | 3.7406540 |
| C | -9.3198700 | -0.4749170 | 6.6764890 |
| C | -9.3884370 | -1.2140010 | 5.4690300 |
| H | -10.3437960 | -1.6512420 | 5.2061580 |
| H | -8.0665150 | 6.5912240 | -4.5918840 |
| H | -8.3599750 | 4.2193100 | -0.1865700 |
| C | -9.3183600 | 6.0245780 | -2.9294170 |
| C | -9.3876440 | 5.3499380 | -1.6848320 |
| H | -10.3430260 | 5.3423980 | -1.1747570 |
| O | -10.4864120 | -6.1799130 | -4.0367900 |
| O | -10.4912470 | 6.5847890 | -3.3252850 |
| O | -10.4933210 | -0.4125200 | 7.3585920 |
| C | -10.5060330 | 7.2784580 | -4.5510350 |
| C | -10.4992040 | -7.5882770 | -4.0235520 |
| C | -10.5088710 | 0.3005650 | 8.5731360 |
| H | -10.2547560 | 1.3566310 | 8.4220100 |
| H | -11.5259680 | 0.2276040 | 8.9540880 |
| H | -9.8172860 | -0.1359380 | 9.3037170 |
| H | -10.2449220 | -7.9828710 | -3.0324360 |
| H | -11.5155620 | -7.8842660 | -4.2772200 |
| H | -9.8063600 | -8.0029320 | -4.7655800 |
| H | -10.2523260 | 6.6182240 | -5.3891230 |
| H | -11.5228020 | 7.6455480 | -4.6790100 |
| H | -9.8137620 | 8.1288630 | -4.5393540 |
| C | 2.5301820 | 1.3947260 | 0.1277800 |
| C | 2.5294250 | -1.3912680 | -0.1578360 |
| C | 2.5301080 | 0.8323190 | -1.1269070 |
| C | 2.5295780 | 0.5598390 | 1.2832040 |
| C | 2.5294640 | -0.8080360 | 1.1429230 |
| C | 2.5299170 | -0.5858530 | -1.2722330 |
| C | 1.9408930 | 1.4458310 | -2.2223500 |
| C | 1.4883820 | 2.7499900 | -2.0149930 |
| H | 0.9997600 | 3.3015840 | -2.8123750 |
| C | 1.9414130 | 2.6209850 | 0.3985290 |
| C | 1.4902650 | 3.3330210 | -0.7140930 |
| H | 1.0058530 | 4.2974880 | -0.5960760 |
| C | 1.9410880 | -0.9641690 | -2.4696670 |
| C | 1.4890050 | -2.2835100 | -2.5299200 |
| H | 1.0043860 | -2.6633320 | -3.4241150 |
| C | 1.4866130 | -3.1186190 | -1.3744880 |
| H | 0.9974340 | -4.0847320 | -1.4534950 |
| C | 1.9395700 | -2.6464590 | -0.1414030 |
| C | 1.9402180 | -1.6554690 | 2.0692180 |
| C | 1.4882560 | -1.0476960 | 3.2418250 |
| H | 1.0036870 | -1.6320020 | 4.0180270 |
| C | 1.4864280 | 0.3704900 | 3.3873490 |
| H | 0.9971320 | 0.7853930 | 4.2633410 |
| C | 1.9397800 | 1.2020130 | 2.3619100 |
| C | 1.6644810 | -2.9943440 | 1.3418870 |
| H | 2.3089200 | -3.8018420 | 1.7033180 |
| H | 0.6271350 | -3.3104860 | 1.4827020 |
| C | 1.6662220 | 0.3353350 | -3.2655060 |
| H | 2.3119870 | 0.4262730 | -4.1446860 |
| H | 0.6291920 | 0.3716440 | -3.6107350 |
| C | 1.6651860 | 2.6606040 | 1.9215780 |
| H | 2.3106270 | 3.3763200 | 2.4403700 |
| H | 0.6281240 | 2.9417580 | 2.1249720 |
| C | 6.2086290 | 0.5869680 | 1.2720460 |
| C | 6.2092780 | -0.5603570 | -1.2833050 |
| C | 6.2093310 | 1.3916710 | 0.1571070 |
| C | 6.2084310 | -0.8317960 | 1.1273810 |
| C | 6.2087470 | -1.3949190 | -0.1270390 |
| C | 6.2095380 | 0.8075760 | -1.1438350 |
| C | 5.6146980 | 2.6449280 | 0.1392790 |
| C | 5.1543310 | 3.1145980 | 1.3695280 |
| H | 4.6565700 | 4.0764390 | 1.4436910 |
| C | 5.6130590 | 0.9648960 | 2.4667200 |
| C | 5.1535190 | 2.2807240 | 2.5248450 |
| H | 4.6550880 | 2.6543280 | 3.4138850 |
| C | 5.6150090 | 1.6535550 | -2.0686660 |
| C | 5.1549080 | 1.0462530 | -3.2371770 |
| H | 4.6566920 | 1.6297410 | -4.0051100 |
| C | 5.1547280 | -0.3712300 | -3.3818000 |
| H | 4.6562360 | -0.7874770 | -4.2516720 |
| C | 5.6144390 | -1.2021220 | -2.3599050 |
| C | 5.6136690 | -2.6185910 | -0.3975460 |
| C | 5.1531930 | -3.3268230 | 0.7125040 |
| H | 4.6539080 | -4.2830530 | 0.5910700 |
| C | 5.1528370 | -2.7433410 | 2.0122920 |
| H | 4.6540020 | -3.2884240 | 2.8075160 |
| C | 5.6128120 | -1.4430080 | 2.2210640 |
| C | 5.3351300 | -2.6585790 | -1.9189320 |
| H | 5.9757780 | -3.3786830 | -2.4382800 |
| H | 4.2955600 | -2.9333030 | -2.1175040 |
| C | 5.3368520 | 2.9913660 | -1.3428120 |
| H | 5.9789210 | 3.8003100 | -1.7059180 |
| H | 4.2979000 | 3.3022680 | -1.4825460 |
| C | 5.3335880 | -0.3327130 | 3.2618190 |
| H | 5.9741770 | -0.4229390 | 4.1452050 |
| H | 4.2940440 | -0.3670120 | 3.5990740 |
| C | 9.9082360 | -0.5889790 | -1.2742200 |
| C | 9.9083780 | 0.5593570 | 1.2885900 |
| C | 9.9075950 | -1.3964100 | -0.1573970 |
| C | 9.9089750 | 0.8337870 | -1.1280590 |
| C | 9.9090670 | 1.3972580 | 0.1295970 |
| C | 9.9077010 | -0.8116080 | 1.1478060 |
| C | 9.3011170 | -2.6440610 | -0.1405690 |
| C | 8.8260710 | -3.1075220 | -1.3687410 |
| H | 8.3145590 | -4.0624200 | -1.4379710 |
| C | 9.3026830 | -0.9636570 | -2.4648710 |
| C | 8.8270310 | -2.2748450 | -2.5205660 |
| H | 8.3162560 | -2.6393350 | -3.4063190 |
| C | 9.3013240 | -1.6545890 | 2.0677350 |
| C | 8.8261190 | -1.0465410 | 3.2309690 |
| H | 8.3142700 | -1.6306260 | 3.9893840 |
| C | 8.8268340 | 0.3672460 | 3.3762510 |
| H | 8.3157360 | 0.7854480 | 4.2377590 |
| C | 9.3027790 | 1.1984910 | 2.3608100 |
| C | 9.3041750 | 2.6161700 | 0.3999850 |
| C | 8.8299830 | 3.3203440 | -0.7081220 |
| H | 8.3198780 | 4.2701420 | -0.5816480 |
| C | 8.8297950 | 2.7393030 | -2.0051160 |
| H | 8.3196050 | 3.2771280 | -2.7980750 |
| C | 9.3037870 | 1.4435120 | -2.2173590 |
| C | 9.0179380 | 2.6539680 | 1.9201120 |
| H | 9.6465670 | 3.3803590 | 2.4458460 |
| H | 7.9739910 | 2.9184320 | 2.1107320 |
| C | 9.0148660 | -2.9898120 | 1.3402140 |
| H | 9.6427610 | -3.8085870 | 1.7070340 |
| H | 7.9706720 | -3.2864240 | 1.4734140 |
| C | 9.0176880 | 0.3343120 | -3.2574740 |
| H | 9.6469070 | 0.4257680 | -4.1491030 |
| H | 7.9739780 | 0.3682320 | -3.5824660 |

Total energy: -5226.424532 Hartree

**SC**•(**sumanene**)_4_

| Symbol | X | Y | Z |
| --- | --- | --- | --- |
| C | -2.9021934 | -0.8403481 | 1.1191535 |
| C | -2.9039547 | 0.5820552 | 1.2746398 |
| C | -2.9012038 | 1.3927818 | 0.1673850 |
| C | -2.9030725 | 0.8162779 | -1.1422922 |
| C | -2.9014333 | -0.5480116 | -1.2907441 |
| C | -2.9041218 | -1.3939812 | -0.1365806 |
| C | -3.5442263 | -1.1701298 | -2.3552387 |
| C | -3.5463111 | -2.5974837 | -0.4086252 |
| C | -3.5455856 | -1.4505896 | 2.1901263 |
| C | -3.5456796 | 0.9488817 | 2.4530120 |
| C | -3.5432571 | 2.6260303 | 0.1604872 |
| C | -3.5440980 | 1.6539552 | -2.0490381 |
| C | -4.0796283 | -3.2900780 | 0.6693400 |
| C | -4.0841771 | -2.7104106 | 1.9729114 |
| C | -4.0773075 | 2.2293415 | 2.5140087 |
| C | -4.0810136 | 3.0685017 | 1.3602435 |
| C | -4.0814984 | -0.3518281 | -3.3381917 |
| C | -4.0765505 | 1.0669972 | -3.1883075 |
| C | -3.7845046 | -2.6356553 | -1.9399766 |
| C | -3.7852898 | -0.3579692 | 3.2514919 |
| C | -3.7826627 | 2.9991529 | -1.3164391 |
| H | -3.0931689 | -3.3293503 | -2.4260336 |
| H | -3.0906740 | 3.7668199 | -1.6730864 |
| H | -3.0941242 | -0.4324812 | 4.0953284 |
| H | -4.6063198 | 2.5668971 | 3.3978487 |
| H | -4.6286842 | 4.0030907 | 1.4109470 |
| H | -4.6050946 | 1.6641038 | -3.9225261 |
| H | -4.6297261 | -0.7747247 | -4.1728006 |
| H | -4.6328840 | -3.2212418 | 2.7564727 |
| H | -4.6088735 | -4.2240742 | 0.5194022 |
| O | -5.1192207 | -2.9956036 | -2.2880402 |
| O | -5.1173947 | 3.4803725 | -1.4543165 |
| O | -5.1203040 | -0.4787486 | 3.7362774 |
| C | -5.4001105 | 4.1734054 | -2.5811517 |
| C | -5.4032811 | 0.1511230 | 4.8994482 |
| C | -5.4009242 | -4.3182249 | -2.3238182 |
| O | -4.5987648 | -5.1810422 | -2.0811485 |
| O | -4.5992185 | 4.3944519 | -3.4509158 |
| O | -4.6022317 | 0.7937927 | 5.5256197 |
| C | -6.8011085 | -4.5332558 | -2.6823334 |
| C | -7.4617315 | -5.7254075 | -2.8329940 |
| N | -7.6672846 | -3.4900623 | -2.9160128 |
| C | -8.8072590 | -5.4090018 | -3.1767848 |
| H | -7.0164384 | -6.6997362 | -2.7092357 |
| C | -8.9000325 | -4.0023085 | -3.2178079 |
| H | -7.4100797 | -2.5208175 | -2.8494322 |
| C | -6.8033885 | -0.0543707 | 5.2640879 |
| C | -7.4656310 | 0.4091562 | 6.3717891 |
| N | -7.6675853 | -0.7798016 | 4.4766834 |
| C | -8.8100972 | -0.0498220 | 6.2687419 |
| H | -7.0220215 | 1.0040298 | 7.1542462 |
| C | -8.9006047 | -0.7881546 | 5.0705494 |
| H | -7.4093796 | -1.2056950 | 3.6038043 |
| C | -6.8002363 | 4.5917065 | -2.5853196 |
| C | -7.4627758 | 5.3196294 | -3.5400381 |
| N | -7.6642656 | 4.2713124 | -1.5636419 |
| C | -8.8073371 | 5.4589245 | -3.0909557 |
| H | -7.0192944 | 5.7008637 | -4.4460673 |
| C | -8.8975289 | 4.7894091 | -1.8529205 |
| H | -7.4055521 | 3.7276764 | -0.7589484 |
| C | -10.1001528 | 4.7338740 | -1.1358997 |
| C | -9.9444901 | 6.0932140 | -3.6319572 |
| C | -10.1043039 | -3.3562936 | -3.5275606 |
| C | -9.9433118 | -6.1972254 | -3.4527706 |
| C | -9.9469255 | 0.1019964 | 7.0889110 |
| C | -10.1033172 | -1.3819051 | 4.6649698 |
| H | -10.1754080 | -2.2753368 | -3.5599433 |
| H | -9.8678641 | -7.2758929 | -3.4215661 |
| C | -11.1982783 | -4.1405645 | -3.7918800 |
| C | -11.1251056 | -5.5556402 | -3.7566344 |
| H | -12.1553278 | -3.6972252 | -4.0378187 |
| H | -9.8736518 | 0.6678763 | 8.0079371 |
| H | -10.1726985 | -1.9498658 | 3.7445452 |
| C | -11.1271423 | -0.4845561 | 6.6843681 |
| C | -11.1980540 | -1.2218788 | 5.4759583 |
| H | -12.1539393 | -1.6586462 | 5.2142219 |
| H | -9.8713137 | 6.6072387 | -4.5809604 |
| H | -10.1691334 | 4.2201402 | -0.1841189 |
| C | -11.1246456 | 6.0349816 | -2.9216468 |
| C | -11.1951823 | 5.3561075 | -1.6794166 |
| H | -12.1510190 | 5.3471154 | -1.1702264 |
| O | -12.2968688 | -6.1810013 | -4.0427835 |
| O | -12.2969870 | 6.5969976 | -3.3163536 |
| O | -12.2991149 | -0.4231153 | 7.3689487 |
| C | -12.3110463 | 7.2943187 | -4.5400800 |
| C | -12.3083613 | -7.5894462 | -4.0328062 |
| C | -12.3119511 | 0.2883804 | 8.5844844 |
| H | -12.0585068 | 1.3447083 | 8.4341190 |
| H | -13.3280968 | 0.2146151 | 8.9677916 |
| H | -11.6184957 | -0.1488588 | 9.3128395 |
| H | -12.0521575 | -7.9860641 | -3.0429994 |
| H | -13.3248924 | -7.8856638 | -4.2854670 |
| H | -11.6164795 | -8.0018968 | -4.7769355 |
| H | -12.0570760 | 6.6365044 | -5.3799810 |
| H | -13.3276803 | 7.6619608 | -4.6674219 |
| H | -11.6186139 | 8.1445432 | -4.5255256 |
| C | 0.7206067 | 1.3941911 | 0.1282643 |
| C | 0.7191697 | -1.3914183 | -0.1623772 |
| C | 0.7198239 | 0.8340052 | -1.1274948 |
| C | 0.7207450 | 0.5571575 | 1.2822624 |
| C | 0.7201836 | -0.8104995 | 1.1394796 |
| C | 0.7191683 | -0.5839144 | -1.2753812 |
| C | 0.1293763 | 1.4494494 | -2.2213789 |
| C | -0.3226502 | 2.7533430 | -2.0116036 |
| H | -0.8121359 | 3.3059671 | -2.8078236 |
| C | 0.1316668 | 2.6199143 | 0.4014602 |
| C | -0.3199820 | 3.3341456 | -0.7096501 |
| H | -0.8054323 | 4.2978355 | -0.5895960 |
| C | 0.1287142 | -0.9600006 | -2.4727232 |
| C | -0.3230361 | -2.2792749 | -2.5352117 |
| H | -0.8081543 | -2.6571902 | -3.4299647 |
| C | -0.3247078 | -3.1165086 | -1.3813612 |
| H | -0.8145381 | -4.0821838 | -1.4615686 |
| C | 0.1288168 | -2.6464435 | -0.1477217 |
| C | 0.1308619 | -1.6592911 | 2.0645980 |
| C | -0.3200793 | -1.0536351 | 3.2386563 |
| H | -0.8049368 | -1.6392638 | 4.0137080 |
| C | -0.3208928 | 0.3642329 | 3.3868526 |
| H | -0.8098033 | 0.7775376 | 4.2637945 |
| C | 0.1316085 | 1.1975039 | 2.3624475 |
| C | -0.1462960 | -2.9965494 | 1.3351669 |
| H | 0.4973086 | -3.8053556 | 1.6950582 |
| H | -1.1839814 | -3.3116266 | 1.4755696 |
| C | -0.1483718 | 0.3406708 | -3.2655806 |
| H | 0.4950652 | 0.4326344 | -4.1461409 |
| H | -1.1863525 | 0.3774482 | -3.6079270 |
| C | -0.1431643 | 2.6569390 | 1.9248493 |
| H | 0.5025115 | 3.3724258 | 2.4437157 |
| H | -1.1797855 | 2.9380205 | 2.1300588 |
| C | 4.3983055 | 0.5811848 | 1.2734342 |
| C | 4.3983493 | -0.5590454 | -1.2850896 |
| C | 4.3976884 | 1.3889312 | 0.1607401 |
| C | 4.3988631 | -0.8370505 | 1.1250847 |
| C | 4.3975770 | -1.3966013 | -0.1309528 |
| C | 4.3985138 | 0.8086841 | -1.1419644 |
| C | 3.8019541 | 2.6416929 | 0.1467455 |
| C | 3.3422935 | 3.1077797 | 1.3784610 |
| H | 2.8436464 | 4.0688470 | 1.4555221 |
| C | 3.8039008 | 0.9557827 | 2.4696377 |
| C | 3.3435073 | 2.2709023 | 2.5317556 |
| H | 2.8447193 | 2.6413072 | 3.4218507 |
| C | 3.8038307 | 1.6575292 | -2.0643707 |
| C | 3.3444665 | 1.0536323 | -3.2348130 |
| H | 2.8477589 | 1.6391358 | -4.0020704 |
| C | 3.3435318 | -0.3635804 | -3.3829156 |
| H | 2.8455615 | -0.7770376 | -4.2543669 |
| C | 3.8024909 | -1.1977111 | -2.3632103 |
| C | 3.8007547 | -2.6189393 | -0.4039234 |
| C | 3.3416233 | -3.3303424 | 0.7045295 |
| H | 2.8419241 | -4.2860210 | 0.5810356 |
| C | 3.3434181 | -2.7505264 | 2.0062233 |
| H | 2.8451287 | -3.2976181 | 2.8004097 |
| C | 3.8043065 | -1.4511976 | 2.2179318 |
| C | 3.5186454 | -2.6539824 | -1.9247839 |
| H | 4.1534087 | -3.3773318 | -2.4467906 |
| H | 2.4773304 | -2.9237618 | -2.1213773 |
| C | 3.5222257 | 2.9922039 | -1.3343211 |
| H | 4.1598340 | 3.8070682 | -1.6923698 |
| H | 2.4815511 | 3.2984988 | -1.4717647 |
| C | 3.5266675 | -0.3438220 | 3.2621854 |
| H | 4.1703260 | -0.4359370 | 4.1429311 |
| H | 2.4881322 | -0.3794837 | 3.6024365 |
| C | 8.0791096 | -0.5953452 | -1.2668431 |
| C | 8.0759405 | 0.5696516 | 1.2810815 |
| C | 8.0787243 | -1.3925788 | -0.1459020 |
| C | 8.0776236 | 0.8247621 | -1.1318544 |
| C | 8.0758814 | 1.3965228 | 0.1189414 |
| C | 8.0771509 | -0.7993569 | 1.1512013 |
| C | 7.4842646 | -2.6454012 | -0.1198811 |
| C | 7.0245994 | -3.1239807 | -1.3472087 |
| H | 6.5238729 | -4.0846850 | -1.4158340 |
| C | 7.4832988 | -0.9816324 | -2.4582746 |
| C | 7.0236339 | -2.2979120 | -2.5076076 |
| H | 6.5219008 | -2.6764119 | -3.3928212 |
| C | 7.4811135 | -1.6390714 | 2.0810249 |
| C | 7.0209316 | -1.0239234 | 3.2453450 |
| H | 6.5198340 | -1.6005811 | 4.0165799 |
| C | 7.0201743 | 0.3942857 | 3.3802634 |
| H | 6.5195572 | 0.8154934 | 4.2465883 |
| C | 7.4794965 | 1.2181975 | 2.3526860 |
| C | 7.4776892 | 2.6205993 | 0.3805993 |
| C | 7.0145088 | 3.3195395 | -0.7341469 |
| H | 6.5071660 | 4.2724119 | -0.6206898 |
| C | 7.0155328 | 2.7269176 | -2.0294410 |
| H | 6.5109335 | 3.2626346 | -2.8271014 |
| C | 7.4799552 | 1.4270382 | -2.2292941 |
| C | 7.1956859 | 2.6702106 | 1.9008012 |
| H | 7.8315921 | 3.3967575 | 2.4170149 |
| H | 6.1541214 | 2.9407614 | 2.0945449 |
| C | 7.2006587 | -2.9807376 | 1.3634982 |
| H | 7.8377601 | -3.7899045 | 1.7350769 |
| H | 6.1591791 | -3.2844950 | 1.5005702 |
| C | 7.1979375 | 0.3094665 | -3.2609746 |
| H | 7.8321891 | 0.3947040 | -4.1492928 |
| H | 6.1551475 | 0.3392889 | -3.5896951 |
| C | 11.7796279 | 0.8039576 | -1.1503233 |
| C | 11.7785804 | -0.8259777 | 1.1367579 |
| C | 11.7815497 | -0.5679658 | -1.2818649 |
| C | 11.7780044 | 1.3977546 | 0.1509430 |
| C | 11.7771652 | 0.5978130 | 1.2732724 |
| C | 11.7812547 | -1.3981572 | -0.1170901 |
| C | 11.1763522 | -1.2144366 | -2.3499365 |
| C | 10.6988177 | -0.3906106 | -3.3705066 |
| H | 10.1862565 | -0.8147198 | -4.2281852 |
| C | 11.1724748 | 1.6401165 | -2.0761500 |
| C | 10.6969627 | 1.0240433 | -3.2348675 |
| H | 10.1831978 | 1.6021802 | -3.9964719 |
| C | 11.1763685 | -2.6187806 | -0.3799475 |
| C | 10.6990311 | -3.3149695 | 0.7316825 |
| H | 10.1871375 | -4.2643935 | 0.6100882 |
| C | 10.6957806 | -2.7247936 | 2.0244590 |
| H | 10.1815666 | -3.2558961 | 2.8193094 |
| C | 11.1705185 | -1.4280952 | 2.2288187 |
| C | 11.1687669 | 0.9804274 | 2.4599851 |
| C | 10.6922218 | 2.2916180 | 2.5058924 |
| H | 10.1778984 | 2.6612847 | 3.3873706 |
| C | 10.6931986 | 3.1164829 | 1.3486582 |
| H | 10.1802474 | 4.0711603 | 1.4098612 |
| C | 11.1706676 | 2.6448616 | 0.1247895 |
| C | 10.8803718 | -0.3119053 | 3.2601151 |
| H | 11.5044107 | -0.3974958 | 4.1559482 |
| H | 9.8345213 | -0.3432456 | 3.5785491 |
| C | 10.8918642 | -2.6670532 | -1.8998908 |
| H | 11.5216681 | -3.3965127 | -2.4200442 |
| H | 9.8483280 | -2.9334628 | -2.0899064 |
| C | 10.8843638 | 2.9799826 | -1.3582543 |
| H | 11.5108377 | 3.7971739 | -1.7309510 |
| H | 9.8395811 | 3.2738533 | -1.4931003 |

Total energy: -6033.799123 Hartree

(**sumanene**)_3_

| Symbol | X | Y | Z |
| --- | --- | --- | --- |
| C | 0.66225 | 1.3131 | 0.48771 |
| C | 0.66350 | -1.3030 | -0.51342 |
| C | 0.66249 | 1.0966 | -0.87020 |
| C | 0.66269 | 0.20584 | 1.3863 |
| C | 0.66327 | -1.0785 | 0.89489 |
| C | 0.66308 | -0.23528 | -1.3799 |
| C | 0.067133 | 1.9718 | -1.7668 |
| C | -0.39212 | 3.1731 | -1.2265 |
| H | -0.88956 | 3.9073 | -1.8525 |
| C | 0.066616 | 2.4237 | 1.0675 |
| C | -0.39218 | 3.3974 | 0.18013 |
| H | -0.88971 | 4.2899 | 0.54666 |
| C | 0.068158 | -0.28872 | -2.6319 |
| C | -0.38996 | -1.5442 | -3.0317 |
| H | -0.88711 | -1.6733 | -3.9881 |
| C | -0.38959 | -2.6504 | -2.1341 |
| H | -0.88634 | -3.5599 | -2.4572 |
| C | 0.068930 | -2.5174 | -0.82338 |
| C | 0.068492 | -2.1364 | 1.5669 |
| C | -0.39067 | -1.8551 | 2.8537 |
| H | -0.88812 | -2.6191 | 3.4432 |
| C | -0.39160 | -0.52455 | 3.3627 |
| H | -0.88925 | -0.34985 | 4.3115 |
| C | 0.067098 | 0.54436 | 2.5926 |
| C | -0.21217 | -3.2376 | 0.51687 |
| H | 0.42491 | -4.1168 | 0.65726 |
| H | -1.2534 | -3.5679 | 0.56932 |
| C | -0.21299 | 1.1711 | -3.0608 |
| H | 0.42533 | 1.4892 | -3.8914 |
| H | -1.2539 | 1.2905 | -3.3742 |
| C | -0.21422 | 2.0652 | 2.5462 |
| H | 0.42357 | 2.6261 | 3.2369 |
| H | -1.2553 | 2.2760 | 2.8063 |
| C | 4.3614 | 0.23727 | 1.3836 |
| C | 4.3620 | -0.20460 | -1.3895 |
| C | 4.3611 | 1.3074 | 0.51541 |
| C | 4.3621 | -1.0983 | 0.87236 |
| C | 4.3624 | -1.3152 | -0.48851 |
| C | 4.3613 | 1.0824 | -0.89690 |
| C | 3.7557 | 2.5170 | 0.82379 |
| C | 3.2811 | 2.6452 | 2.1303 |
| H | 2.7708 | 3.5498 | 2.4459 |
| C | 3.7563 | 0.28943 | 2.6309 |
| C | 3.2816 | 1.5413 | 3.0259 |
| H | 2.7721 | 1.6636 | 3.9766 |
| C | 3.7559 | 2.1362 | -1.5660 |
| C | 3.2815 | 1.8520 | -2.8477 |
| H | 2.7709 | 2.6137 | -3.4287 |
| C | 3.2823 | 0.52439 | -3.3560 |
| H | 2.7723 | 0.34489 | -4.2973 |
| C | 3.7573 | -0.54270 | -2.5915 |
| C | 3.7580 | -2.4219 | -1.0669 |
| C | 3.2840 | -3.3902 | -0.18008 |
| H | 2.7745 | -4.2747 | -0.54947 |
| C | 3.2833 | -3.1664 | 1.2235 |
| H | 2.7739 | -3.8926 | 1.8492 |
| C | 3.7572 | -1.9706 | 1.7657 |
| C | 3.4731 | -2.0630 | -2.5447 |
| H | 4.1034 | -2.6272 | -3.2403 |
| H | 2.4298 | -2.2699 | -2.7991 |
| C | 3.4707 | 3.2364 | -0.51609 |
| H | 4.1001 | 4.1214 | -0.65706 |
| H | 2.4271 | 3.5594 | -0.56760 |
| C | 3.4720 | -1.1701 | 3.0588 |
| H | 4.1018 | -1.4902 | 3.8955 |
| H | 2.4285 | -1.2876 | 3.3644 |
| C | -3.0061 | 1.0782 | -0.89669 |
| C | -3.0025 | -1.0980 | 0.87135 |
| C | -3.0051 | -0.20617 | -1.3874 |
| C | -3.0054 | 1.3040 | 0.51421 |
| C | -3.0037 | 0.23680 | 1.3812 |
| C | -3.0032 | -1.3152 | -0.48648 |
| C | -3.6285 | -0.54560 | -2.5841 |
| C | -4.1354 | 0.51034 | -3.3356 |
| H | -4.6633 | 0.32737 | -4.2661 |
| C | -3.6304 | 2.1285 | -1.5625 |
| C | -4.1359 | 1.8422 | -2.8270 |
| H | -4.6645 | 2.5984 | -3.3987 |
| C | -3.6253 | -2.4182 | -1.0629 |
| C | -4.1286 | -3.3713 | -0.18256 |
| H | -4.6556 | -4.2455 | -0.55145 |
| C | -4.1277 | -3.1461 | 1.2253 |
| H | -4.6538 | -3.8617 | 1.8492 |
| C | -3.6237 | -1.9661 | 1.7639 |
| C | -3.6258 | 0.28774 | 2.6248 |
| C | -4.1319 | 1.5255 | 3.0098 |
| H | -4.6587 | 1.6422 | 3.9516 |
| C | -4.1335 | 2.6322 | 2.1109 |
| H | -4.6615 | 3.5291 | 2.4187 |
| C | -3.6291 | 2.5097 | 0.81978 |
| C | -3.8804 | -1.1718 | 3.0644 |
| H | -3.2033 | -1.4784 | 3.8685 |
| H | -4.9029 | -1.3097 | 3.4230 |
| C | -3.8843 | -2.0692 | -2.5459 |
| H | -3.2097 | -2.6125 | -3.2159 |
| H | -4.9077 | -2.3108 | -2.8413 |
| C | -3.8883 | 3.2380 | -0.51821 |
| H | -3.2153 | 4.0909 | -0.65518 |
| H | -4.9124 | 3.6133 | -0.57768 |

Total energy: -2422.0774 Hartree

(**sumanene**)_5_

| Symbol | X | Y | Z |
| --- | --- | --- | --- |
| C | 6.6804 | 0.31293 | -1.3675 |
| C | 6.6794 | -0.28351 | 1.3724 |
| C | 6.6812 | 1.3303 | -0.44241 |
| C | 6.6793 | -1.0483 | -0.93266 |
| C | 6.6787 | -1.3407 | 0.41097 |
| C | 6.6806 | 1.0263 | 0.95387 |
| C | 7.3051 | 2.5510 | -0.67979 |
| C | 7.8102 | 2.7453 | -1.9620 |
| H | 8.3386 | 3.6579 | -2.2188 |
| C | 7.3031 | 0.43314 | -2.6058 |
| C | 7.8092 | 1.6906 | -2.9212 |
| H | 8.3368 | 1.8594 | -3.8545 |
| C | 7.3042 | 2.0378 | 1.6777 |
| C | 7.8086 | 1.6815 | 2.9248 |
| H | 8.3368 | 2.4046 | 3.5380 |
| C | 7.8073 | 0.32342 | 3.3587 |
| H | 8.3345 | 0.088902 | 4.2780 |
| C | 7.3016 | -0.68911 | 2.5489 |
| C | 7.3005 | -2.4739 | 0.92564 |
| C | 7.8050 | -3.3764 | -0.0059620 |
| H | 8.3318 | -4.2696 | 0.31429 |
| C | 7.8056 | -3.0731 | -1.3990 |
| H | 8.3327 | -3.7525 | -2.0612 |
| C | 7.3016 | -1.8650 | -1.8715 |
| C | 7.5583 | -2.2081 | 2.4259 |
| H | 6.8841 | -2.7885 | 3.0645 |
| H | 8.5818 | -2.4650 | 2.7078 |
| C | 7.5635 | 3.2036 | 0.69692 |
| H | 6.8905 | 4.0477 | 0.88063 |
| H | 8.5876 | 3.5748 | 0.77807 |
| C | 7.5601 | -0.99947 | -3.1253 |
| H | 6.8866 | -1.2620 | -3.9479 |
| H | 8.5839 | -1.1160 | -3.4876 |
| C | 3.0138 | -1.0263 | -0.95257 |
| C | 3.0140 | 1.0458 | 0.93229 |
| C | 3.0138 | 0.28358 | -1.3708 |
| C | 3.0139 | -1.3299 | 0.44076 |
| C | 3.0140 | -0.31264 | 1.3660 |
| C | 3.0138 | 1.3384 | -0.41131 |
| C | 3.6088 | 0.68991 | -2.5564 |
| C | 4.0673 | -0.33371 | -3.3858 |
| H | 4.5647 | -0.10550 | -4.3234 |
| C | 3.6088 | -2.0445 | -1.6833 |
| C | 4.0672 | -1.6910 | -2.9525 |
| H | 4.5645 | -2.4203 | -3.5843 |
| C | 3.6087 | 2.4803 | -0.92768 |
| C | 4.0676 | 3.4026 | 0.012951 |
| H | 4.5653 | 4.3142 | -0.30299 |
| C | 4.0678 | 3.0994 | 1.4051 |
| H | 4.5659 | 3.7970 | 2.0713 |
| C | 3.6090 | 1.8694 | 1.8769 |
| C | 3.6088 | -0.43640 | 2.6132 |
| C | 4.0673 | -1.7122 | 2.9416 |
| H | 4.5653 | -1.8946 | 3.8888 |
| C | 4.0672 | -2.7662 | 1.9829 |
| H | 4.5648 | -3.6921 | 2.2541 |
| C | 3.6087 | -2.5598 | 0.68170 |
| C | 3.8903 | 0.99699 | 3.1234 |
| H | 3.2532 | 1.2677 | 3.9716 |
| H | 4.9318 | 1.0985 | 3.4414 |
| C | 3.8904 | 2.2056 | -2.4240 |
| H | 3.2539 | 2.8050 | -3.0830 |
| H | 4.9320 | 2.4300 | -2.6705 |
| C | 3.8901 | -3.2031 | -0.69702 |
| H | 3.2529 | -4.0729 | -0.88683 |
| H | 4.9313 | -3.5299 | -0.76813 |
| C | -0.66185 | 0.31461 | -1.3649 |
| C | -0.66198 | -0.28068 | 1.3723 |
| C | -0.66183 | 1.3322 | -0.44001 |
| C | -0.66226 | -1.0438 | -0.93071 |
| C | -0.66240 | -1.3360 | 0.41309 |
| C | -0.66197 | 1.0292 | 0.95358 |
| C | -0.065536 | 2.5615 | -0.68126 |
| C | 0.39575 | 2.7666 | -1.9816 |
| H | 0.89754 | 3.6905 | -2.2515 |
| C | -0.065940 | 0.43767 | -2.6117 |
| C | 0.39553 | 1.7124 | -2.9399 |
| H | 0.89716 | 1.8930 | -3.8854 |
| C | -0.065680 | 2.0472 | 1.6835 |
| C | 0.39608 | 1.6938 | 2.9514 |
| H | 0.89821 | 2.4222 | 3.5803 |
| C | 0.39626 | 0.33676 | 3.3852 |
| H | 0.89845 | 0.10835 | 4.3200 |
| C | -0.065591 | -0.68662 | 2.5575 |
| C | -0.066683 | -2.4774 | 0.92957 |
| C | 0.39384 | -3.3993 | -0.010479 |
| H | 0.89510 | -4.3087 | 0.30589 |
| C | 0.39383 | -3.0967 | -1.4026 |
| H | 0.89507 | -3.7927 | -2.0679 |
| C | -0.066618 | -1.8676 | -1.8748 |
| C | 0.21597 | -2.2021 | 2.4254 |
| H | -0.42037 | -2.8015 | 3.0847 |
| H | 1.2578 | -2.4261 | 2.6710 |
| C | 0.21606 | 3.2051 | 0.69698 |
| H | -0.42046 | 4.0756 | 0.88641 |
| H | 1.2576 | 3.5306 | 0.76786 |
| C | 0.21567 | -0.99573 | -3.1211 |
| H | -0.42066 | -1.2671 | -3.9698 |
| H | 1.2574 | -1.0974 | -3.4378 |
| C | -4.3377 | -1.0269 | -0.95202 |
| C | -4.3399 | 1.0463 | 0.93220 |
| C | -4.3384 | 0.28294 | -1.3709 |
| C | -4.3381 | -1.3299 | 0.44170 |
| C | -4.3393 | -0.31218 | 1.3665 |
| C | -4.3396 | 1.3384 | -0.41166 |
| C | -3.7415 | 0.68904 | -2.5556 |
| C | -3.2787 | -0.33402 | -3.3832 |
| H | -2.7754 | -0.10504 | -4.3173 |
| C | -3.7398 | -2.0443 | -1.6815 |
| C | -3.2778 | -1.6908 | -2.9493 |
| H | -2.7739 | -2.4187 | -3.5774 |
| C | -3.7436 | 2.4798 | -0.92797 |
| C | -3.2827 | 3.4016 | 0.012063 |
| H | -2.7802 | 4.3102 | -0.30450 |
| C | -3.2829 | 3.0990 | 1.4040 |
| H | -2.7806 | 3.7942 | 2.0693 |
| C | -3.7441 | 1.8701 | 1.8762 |
| C | -3.7432 | -0.43482 | 2.6133 |
| C | -3.2809 | -1.7092 | 2.9418 |
| H | -2.7782 | -1.8888 | 3.8869 |
| C | -3.2796 | -2.7633 | 1.9838 |
| H | -2.7759 | -3.6862 | 2.2535 |
| C | -3.7405 | -2.5585 | 0.68326 |
| C | -3.4610 | 0.99849 | 3.1223 |
| H | -4.0962 | 1.2699 | 3.9720 |
| H | -2.4186 | 1.0998 | 3.4372 |
| C | -3.4595 | 2.2043 | -2.4234 |
| H | -4.0942 | 2.8041 | -3.0839 |
| H | -2.4170 | 2.4273 | -2.6672 |
| C | -3.4560 | -3.2014 | -0.69465 |
| H | -4.0901 | -4.0736 | -0.88457 |
| H | -2.4134 | -3.5238 | -0.76446 |
| C | -8.0375 | 0.31107 | -1.3711 |
| C | -8.0386 | -0.28528 | 1.3734 |
| C | -8.0387 | 1.3312 | -0.44430 |
| C | -8.0370 | -1.0513 | -0.93534 |
| C | -8.0377 | -1.3439 | 0.41153 |
| C | -8.0392 | 1.0274 | 0.95343 |
| C | -7.4325 | 2.5560 | -0.68406 |
| C | -6.9555 | 2.7570 | -1.9803 |
| H | -6.4420 | 3.6766 | -2.2430 |
| C | -7.4300 | 0.43350 | -2.6125 |
| C | -6.9543 | 1.7052 | -2.9360 |
| H | -6.4400 | 1.8793 | -3.8760 |
| C | -7.4336 | 2.0422 | 1.6804 |
| C | -6.9577 | 1.6871 | 2.9436 |
| H | -6.4446 | 2.4147 | 3.5646 |
| C | -6.9570 | 0.33350 | 3.3766 |
| H | -6.4438 | 0.10189 | 4.3047 |
| C | -7.4321 | -0.68925 | 2.5542 |
| C | -7.4306 | -2.4800 | 0.92699 |
| C | -6.9531 | -3.3958 | -0.011912 |
| H | -6.4391 | -4.2968 | 0.30808 |
| C | -6.9522 | -3.0941 | -1.4006 |
| H | -6.4373 | -3.7810 | -2.0649 |
| C | -7.4290 | -1.8711 | -1.8753 |
| C | -7.1436 | -2.2036 | 2.4219 |
| H | -7.7698 | -2.8080 | 3.0866 |
| H | -6.0986 | -2.4202 | 2.6611 |
| C | -7.1462 | 3.1988 | 0.69376 |
| H | -7.7733 | 4.0762 | 0.88413 |
| H | -6.1015 | 3.5151 | 0.76297 |
| C | -7.1409 | -0.99909 | -3.1203 |
| H | -7.7657 | -1.2734 | -3.9768 |
| H | -6.0953 | -1.0967 | -3.4261 |

Total energy: -4036.8266 Hartree

**SC** ⸦ **MB**

| Symbol | X | Y | Z |
| --- | --- | --- | --- |
| C | 3.732864 | -3.190029 | 0.321809 |
| C | 4.622613 | -2.097925 | 0.602570 |
| C | 4.956750 | -1.205403 | -0.393943 |
| C | 4.388698 | -1.354317 | -1.706203 |
| C | 3.540885 | -2.404530 | -1.975048 |
| C | 3.209333 | -3.345963 | -0.942865 |
| C | 2.491827 | -2.287429 | -2.877969 |
| C | 1.947212 | -3.888102 | -1.152203 |
| C | 3.015123 | -3.555140 | 1.453315 |
| C | 4.498822 | -1.697267 | 1.925868 |
| C | 5.193243 | 0.134151 | -0.119286 |
| C | 4.231867 | -0.116837 | -2.320163 |
| C | 1.306475 | -4.446162 | -0.051069 |
| C | 1.839456 | -4.274166 | 1.254297 |
| C | 4.857179 | -0.390225 | 2.237475 |
| C | 5.208146 | 0.526150 | 1.214235 |
| C | 2.399057 | -1.103106 | -3.598542 |
| C | 3.276279 | -0.018102 | -3.325425 |
| C | 1.466259 | -3.390207 | -2.540771 |
| C | 3.518500 | -2.666242 | 2.622488 |
| C | 4.913928 | 0.934402 | -1.408109 |
| H | 1.443296 | -4.201575 | -3.274157 |
| H | 5.795719 | 1.388717 | -1.867213 |
| H | 3.920300 | -3.222377 | 3.471597 |
| H | 4.724001 | -0.007447 | 3.242982 |
| H | 5.331855 | 1.569820 | 1.480311 |
| H | 3.103194 | 0.927790 | -3.826662 |
| H | 1.577762 | -0.938575 | -4.287273 |
| H | 1.241797 | -4.592064 | 2.100848 |
| H | 0.324066 | -4.889582 | -0.155664 |
| O | 0.183015 | -2.762726 | -2.508411 |
| O | 3.999771 | 1.961148 | -1.024802 |
| O | 2.449310 | -1.817403 | 3.064978 |
| C | 3.812914 | 3.016320 | -1.841506 |
| C | 1.510758 | -2.329333 | 3.889221 |
| C | -0.900422 | -3.570577 | -2.490907 |
| O | -0.837562 | -4.768804 | -2.403019 |
| O | 4.386833 | 3.180061 | -2.885279 |
| O | 1.592423 | -3.407537 | 4.417220 |
| C | -2.133190 | -2.791962 | -2.597590 |
| C | -3.420566 | -3.260168 | -2.538773 |
| N | -2.138039 | -1.432553 | -2.805558 |
| C | -4.274314 | -2.128957 | -2.678697 |
| H | -3.697429 | -4.294624 | -2.412373 |
| C | -3.436966 | -1.005840 | -2.829161 |
| H | -1.337605 | -0.834826 | -2.665054 |
| C | 0.380110 | -1.406072 | 3.985850 |
| C | -0.802738 | -1.594129 | 4.654966 |
| N | 0.328319 | -0.235055 | 3.264281 |
| C | -1.650237 | -0.505162 | 4.303166 |
| H | -1.022796 | -2.429333 | 5.300808 |
| C | -0.914585 | 0.311444 | 3.421282 |
| H | 0.977185 | 0.011270 | 2.530302 |
| C | 2.786430 | 3.889287 | -1.266754 |
| C | 2.262601 | 5.048367 | -1.779424 |
| N | 2.163270 | 3.574477 | -0.080890 |
| C | 1.238442 | 5.466218 | -0.879526 |
| H | 2.591168 | 5.531057 | -2.686252 |
| C | 1.196158 | 4.512907 | 0.158918 |
| C | 0.282092 | 4.622290 | 1.215793 |
| C | 0.347900 | 6.559676 | -0.867963 |
| C | -3.959848 | 0.288594 | -2.939947 |
| C | -5.674274 | -1.964564 | -2.659337 |
| C | -2.981225 | -0.170354 | 4.624782 |
| C | -1.479469 | 1.451109 | 2.836093 |
| H | -3.310437 | 1.150483 | -3.039998 |
| H | -6.314930 | -2.829767 | -2.552378 |
| C | -5.324389 | 0.431358 | -2.905450 |
| C | -6.183507 | -0.687525 | -2.770164 |
| H | -5.786202 | 1.407929 | -2.983899 |
| H | -3.543494 | -0.797915 | 5.303257 |
| H | -0.919712 | 2.056229 | 2.133856 |
| C | -3.531314 | 0.952424 | 4.043007 |
| C | -2.778436 | 1.756427 | 3.151977 |
| H | -3.269526 | 2.619771 | 2.719729 |
| H | 0.389667 | 7.294581 | -1.660669 |
| H | 0.270168 | 3.906097 | 2.028465 |
| C | -0.558454 | 6.652652 | 0.167399 |
| C | -0.586023 | 5.684385 | 1.202505 |
| H | -1.312620 | 5.819459 | 1.994287 |
| O | -7.507342 | -0.375966 | -2.760691 |
| O | -1.479281 | 7.641517 | 0.305790 |
| O | -4.803177 | 1.389067 | 4.248872 |
| C | -1.510107 | 8.652377 | -0.674599 |
| C | -8.428272 | -1.432995 | -2.632201 |
| C | -5.621459 | 0.637340 | 5.112705 |
| H | -5.206434 | 0.597348 | 6.127095 |
| H | -6.583379 | 1.146579 | 5.139164 |
| H | -5.764302 | -0.385284 | 4.742315 |
| H | -8.295553 | -1.969180 | -1.684516 |
| H | -9.416982 | -0.977708 | -2.650543 |
| H | -8.342654 | -2.144100 | -3.462681 |
| H | -1.735247 | 8.242756 | -1.666723 |
| H | -2.304755 | 9.335479 | -0.380060 |
| H | -0.560646 | 9.199337 | -0.717052 |
| C | -0.564613 | -1.803790 | 0.826088 |
| C | -1.072401 | -0.656928 | 0.226764 |
| C | -2.446313 | -0.441818 | 0.163953 |
| C | -3.312828 | -1.374127 | 0.711787 |
| C | -2.806653 | -2.522611 | 1.308070 |
| C | -1.435180 | -2.740992 | 1.359336 |
| H | 0.506229 | -1.957941 | 0.866254 |
| H | -2.836209 | 0.454192 | -0.302004 |
| H | -3.484561 | -3.253137 | 1.734029 |
| C | -0.121769 | 0.348854 | -0.301935 |
| O | -0.497552 | 0.807041 | -1.503332 |
| O | 0.879600 | 0.721589 | 0.269649 |
| C | 0.328756 | 1.810481 | -2.108700 |
| H | 0.016072 | 1.859539 | -3.149273 |
| H | 0.167936 | 2.772523 | -1.620902 |
| H | 1.375487 | 1.520312 | -2.034707 |
| H | 2.203647 | 2.654210 | 0.333940 |
| H | -1.042491 | -3.636351 | 1.825568 |
| H | -4.381489 | -1.204628 | 0.663666 |

Total energy: -3264.220885 Hartree

**trihydroxysumanene** (endo-OH, optimized structure, function/basis set: M06-2X/6-311++G(d,p))

| Symbol | X | Y | Z |
| --- | --- | --- | --- |
| O | -1.803532 | 3.455051 | 1.208311 |
| O | 3.845191 | -0.098048 | 1.218707 |
| C | 0.074099 | 1.404247 | -0.976272 |
| O | -2.100993 | -3.284669 | 1.210097 |
| C | -2.149323 | 1.514834 | -0.308181 |
| C | -1.201460 | 0.740448 | -0.976852 |
| C | 1.137565 | 3.117590 | 0.283350 |
| C | -2.274459 | -1.322321 | -0.307347 |
| C | 2.375179 | 1.096439 | -0.298305 |
| C | 3.274120 | -0.136532 | -0.066097 |
| C | -3.281977 | -0.569762 | 0.276646 |
| C | 1.171363 | -0.772585 | -0.972642 |
| C | -0.016191 | 2.624021 | -0.306389 |
| C | 0.858200 | -3.207933 | 0.284563 |
| C | -0.247554 | -2.614944 | -0.304872 |
| C | 2.272125 | -1.302519 | -0.299306 |
| C | 2.338148 | 2.350473 | 0.288674 |
| C | -0.049763 | -1.407927 | -0.974621 |
| C | -3.219034 | 0.854060 | 0.276219 |
| C | 1.234043 | 0.663609 | -0.972749 |
| C | 2.121271 | -2.548377 | 0.287844 |
| C | -1.771636 | -2.769047 | -0.052820 |
| C | -1.262226 | -0.634765 | -0.976536 |
| C | -1.520715 | 2.911862 | -0.054209 |
| H | -1.469515 | 2.839388 | 1.865161 |
| H | 4.614451 | -0.667084 | 1.225935 |
| H | -1.719723 | -2.696591 | 1.866493 |
| H | 1.104133 | 4.028775 | 0.871097 |
| H | 4.058601 | -0.165710 | -0.836693 |
| H | -4.058081 | -1.053516 | 0.860168 |
| H | 0.745362 | -4.112602 | 0.872494 |
| H | 3.173432 | 2.699652 | 0.884869 |
| H | -3.949291 | 1.404632 | 0.859722 |
| H | 2.917953 | -2.979213 | 0.885426 |
| H | -2.213209 | -3.462263 | -0.775204 |
| H | -1.900089 | 3.640647 | -0.776976 |

Total energy: -1032.946368 Hartree

**trihydroxysumanene** (exo-OH, optimized structure, function/basis set: M06-2X/6-311++G(d,p))

| Symbol | X | Y | Z |
| --- | --- | --- | --- |
| C | 0.876873 | -1.099843 | -0.612154 |
| C | 2.621201 | 0.301927 | 0.021779 |
| C | 1.385338 | 0.244438 | -0.612159 |
| C | 1.233542 | -3.135186 | 0.544808 |
| C | 0.815599 | 2.509198 | 0.022040 |
| C | -1.049057 | -2.420811 | 0.021983 |
| C | -2.545313 | -2.081997 | 0.298660 |
| C | 2.098538 | 2.635784 | 0.545047 |
| C | -1.391007 | -0.209432 | -0.612235 |
| C | 1.765317 | -1.960870 | 0.021751 |
| C | -2.828747 | 1.830628 | 0.544200 |
| C | -1.572086 | 2.118982 | 0.021629 |
| C | -2.580976 | -0.548299 | 0.021508 |
| C | -0.171065 | -3.364728 | 0.544973 |
| C | -0.904256 | 1.077540 | -0.612241 |
| C | 2.999753 | 1.534181 | 0.544867 |
| C | -0.481040 | -1.321818 | -0.612095 |
| C | -3.332269 | 0.499463 | 0.544116 |
| C | -0.530483 | 3.245247 | 0.298757 |
| C | 0.514145 | 1.309430 | -0.612160 |
| C | 3.075878 | -1.163328 | 0.298309 |
| H | 1.865156 | -3.837921 | 1.078265 |
| H | 2.391294 | 3.534031 | 1.078709 |
| H | -3.392391 | 2.588982 | 1.077651 |
| H | -0.546028 | -4.231940 | 1.078559 |
| H | 3.938256 | 1.642938 | 1.078481 |
| H | -4.256707 | 0.303913 | 1.077551 |
| H | 3.348529 | -1.266474 | 1.350920 |
| H | -2.770725 | -2.266171 | 1.351404 |
| H | -0.577643 | 3.532712 | 1.351421 |
| O | -3.480627 | -2.847387 | -0.423739 |
| H | -3.309053 | -2.707568 | -1.357234 |
| O | 4.206010 | -1.590766 | -0.424618 |
| O | -0.725274 | 4.437841 | -0.423972 |
| H | -0.689462 | 4.219115 | -1.357392 |
| H | 3.998592 | -1.512175 | -1.357995 |

Total energy: -460.068581 Hartree

1. (a) Sakurai, H., Daiko, T. & Hirao, T. *Science* **301**, 1878 (2003). (b) Dhotre, B. K., Raut, V. B., Patharia, M. A. & Pathan, M. A. *Russ*. *J. Org. Chem*. **57**, 1135–1140 (2021). (c) Wilson, B. H., Scott, H. S., Qazvini, O. T., Telfer, S. G., Mathonière, C., Clérac, R. & Kruger, P. E. *Chem. Commun.* **54**, 13391–13394 (2018). (d) Frank, A., Bernet, A., Kreger, K. & Schmidt, H.-W. *Soft Matter* **16**, 4564–4568 (2020). (e) Chu, C-W., Srtricker, L., Kirse, T. M., Hayduk, M. & Ravoo, B. J. *Chem. Eur. J.* **25**, 6131–6140 (2019). (f) Ando, T., Kohno, Y., Nakamura, N. & Ohno, H. *Chem. Commun.* **49**, 10248–10250 (2013). [↑](#footnote-ref-2)
2. Lottner, C., Bart, K.-C., Bernhardt, G. & Brunner, H. *J. Med. Chem.* **45**, 2079–2089 (2002). [↑](#footnote-ref-3)
3. Adepu, R., Prasad, B., Ashfaq, M. A., Ehtesham, N. Z. & Pal, M. *RSC Adv.* **4**, 49324–49328 (2014). [↑](#footnote-ref-4)
4. Amaya, T., Hifumi, M., Okada, Y., Shimizu, Y., Moriuchi, T., Segawa, K., Ando, Y. & Hirao, T. *J. Org. Chem.* **76**, 8049–8052 (2011). [↑](#footnote-ref-5)
5. (a) Felorzabihi, N., Froimowicz, P., Haley, J. C., Bardajee, G. R., Li, B., Bovero, E., van Veggel, F. C. J. M. & Winnik, M. A. *J. Phys. Chem. B* **113**, 2262–2272 (2009). (b) Sangghaleh, F., Sychugov, I., Yang, Z., Veinot, J. G. C. & Linnros, J. *ACS Nano* **9**, 7097–7104 (2015). (c) Nakasha, K. & Fukuhara, G. *J. Photochem. Photobiol. A* **426**, 113736 (2022). [↑](#footnote-ref-6)
6. Mizuno, H., Nakazawa, H., Harada, M., Yakiyama, Y., Sakurai, H. & Fukuhara, G. *Chem. Commun.* **59**, 9595–9598 (2023). [↑](#footnote-ref-7)
7. (a) Denkova, P. S., van Lokeren, L., Verbruggen, I. & Willem, R. *J. Phys. Chem. B* **112**, 10935–10941 (2008). (b) Allouche, L., Marquis, A. & Lehn, J.-M. *Chem. Eur. J.* **12**, 7520–7525 (2006). [↑](#footnote-ref-8)
8. Yaws, C. L. *Handbook of viscosity*; vol. 1. (Gulf Publishing Co., Houston, 1995). [↑](#footnote-ref-9)
9. Frisch, M. J., Trucks, G. W., Schlegel, H. B., Scuseria, G. E., Robb, M. A., Cheeseman, J. R., Scalmani, G., Barone, V., Petersson, G. A., Nakatsuji, H., Li, X., Caricato, M., Marenich, A. V., Bloino, J., Janesko, B. G., Gomperts, R., Mennucci, B., Hratchian, H. P., Ortiz, J. V., Izmaylov, A. F., Sonnenberg, J. L., Williams-Young, D., Ding, F., Lipparini, F., Egidi, F., Goings, J., Peng, B., Petrone, A., Henderson, T., Ranasinghe, D., Zakrzewski, V. G., Gao, J., Rega, N., Zheng, G., Liang, W., Hada, M., Ehara, M., Toyota, K., Fukuda, R., Hasegawa, J., Ishida, M., Nakajima, T., Honda, Y., Kitao, O., Nakai, H., Vreven, T., Throssell, K., Montgomery, J. A. Jr., Peralta, J. E., Ogliaro, F., Bearpark, M. J., Heyd, J. J., Brothers, E. N., Kudin, K. N., Staroverov, V. N., Keith, T. A., Kobayashi, R., Normand, J., Raghavachari, K., Rendell, A. P., Burant, J. C., Iyengar, S. S., Tomasi, J., Cossi, M., Millam, J. M., Klene, M., Adamo, C., Cammi, R., Ochterski, J. W., Martin, R. L., Morokuma, K., Farkas, O., Foresman, J. B., Fox, D. J. Gaussian, Inc., Wallingford CT (2016). [↑](#footnote-ref-10)
